# Investigation into the Use of Encorafenib to Develop Potential PROTACs Directed against BRAF^V600E^ Protein

**DOI:** 10.3390/molecules27238513

**Published:** 2022-12-03

**Authors:** Elisabetta Marini, Marco Marino, Giulia Gionfriddo, Federica Maione, Marta Pandini, Daniele Oddo, Marta Giorgis, Barbara Rolando, Federica Blua, Simone Gastaldi, Serena Marchiò, Sandra Kovachka, Francesca Spyrakis, Eleonora Gianquinto, Federica Di Nicolantonio, Massimo Bertinaria

**Affiliations:** 1 Department of Drug Science and Technology, University of Turin, 10125 Torino, Italy; 2 Candiolo Cancer Institute, FPO-IRCCS (Fondazione del Piemonte per l’Oncologia-Istituti di Ricovero e Cura a Carattere Scientifico), 10060 Candiolo, Italy; 3 Department of Oncology, University of Torino, 10060 Torino, Italy; 4 Centre National de la Recherche Scientifique, Institut de Chimie de Nice, Université Côte d’Azur, CEDEX 2, 06108 Nice, France

**Keywords:** BRAF kinase, PROTACs, encorafenib, pomalidomide, molecular dynamics

## Abstract

BRAF is a serine/threonine kinase frequently mutated in human cancers. BRAF^V600E^ mutated protein is targeted through the use of kinase inhibitors which are approved for the treatment of melanoma; however, their long-term efficacy is hampered by resistance mechanisms. The PROTAC-induced degradation of BRAF^V600E^ has been proposed as an alternative strategy to avoid the onset of resistance. In this study, we designed a series of compounds where the BRAF kinase inhibitor encorafenib was conjugated to pomalidomide through different linkers. The synthesized compounds maintained their ability to inhibit the kinase activity of mutated BRAF with IC_50_ values in the 40–88 nM range. Selected compounds inhibited BRAF^V600E^ signaling and cellular proliferation of A375 and Colo205 tumor cell lines. Compounds **10** and **11**, the most active of the series, were not able to induce degradation of mutated BRAF. Docking and molecular dynamic studies, conducted in comparison with the efficient BRAF degrader P5B, suggest that a different orientation of the linker bearing the pomalidomide substructure, together with a decreased mobility of the solvent-exposed part of the conjugates, could explain this behavior.

## 1. Introduction

The RAS/RAF/MEK/ERK signaling pathway controls cellular growth, differentiation, and survival [1,2]. The RAF family of protein kinases (ARAF, BRAF, and CRAF) are critical effectors of this pathway [3], which is normally activated by receptor tyrosine kinase signaling that stimulates binding of GTP to RAS at the plasma membrane [4,5]. RAF proteins are subsequently recruited at the membrane through interaction with the active GTP-bound RAS, where they are activated by dephosphorylation and phosphorylation events [2]. Recruitment of RAFs leads to a cascade of downstream events (ERK-signaling) that involve phosphorylation of MEK1 and MEK2 by RAFs, which in turn phosphorylate and activate ERK1 and ERK2 [6]. Aberrant activation of ERK signaling is a hallmark of many cancers, and is most commonly due to mutations of RAS and BRAF genes [7,8]. A key step in the activation of RAF proteins is the formation of homo- and heterodimers, as RAF monomers are inactive due to autoinhibition by the N-terminal domain [6,9]. While RAF proteins activate ERK signaling as homo- and heterodimers in the presence of active RAS, mutant BRAF^V600E^ can activate ERK signaling independently of RAS as an active monomer [10,11], and promote insensitivity to ERK negative feedback mechanisms [12].

Over 45 oncogenic mutations have been described for BRAF, with the V600E mutation accounting for ∼90% of them [3]. BRAF mutants are present in >50% of melanoma patients as well as in colorectal (5−10%), thyroid carcinomas (25−45%), hairy cell leukemia (∼100%) and less commonly in ovarian and lung malignancies. In colorectal cancer (CRC), the missense mutation V600E in BRAF genes occurs in a mutually exclusive fashion with RAS genes alterations, and often indicates a poor outcome in the metastatic setting [13,14,15,16,17].

Therapeutic efforts to mitigate BRAF^V600E^ enjoyed initial success with the discovery of small-molecule BRAF kinase inhibitors, with the approval of vemurafenib [18], dabrafenib [19], and encorafenib [20] for the treatment of melanoma patients carrying BRAF^V600E^ mutations. Indeed, acquisition of a BRAF mutation in melanoma cells leads to constitutive signaling through the MAPK pathway, which in turn contributes to immune escape through the recruitment of regulatory T cells, decreased antigen presentation (via downregulation of MHC class I) and the inhibition of IFNγ, IL2, and TNFα release. Inhibition of BRAF in BRAF-mutant melanoma cells reverses these processes and can restore tumor–immune recognition [21].

The clinical benefits of currently approved BRAF kinase inhibitors are greatly restrained by the rapid emergence of acquired resistance. The prevailing explanation for this phenomenon invokes the transactivation model, wherein inhibitor occupation of one protomer within the RAF dimer induces kinase activation of the other protomer. Even when BRAF^V600E^ is bound by an inhibitor and can no longer phosphorylate its substrates, the oncogenic protein can still activate CRAF, another isozyme in the RAF family, through dimerization, to transduce proliferative signals through the ERK pathway [22]. Combination with MEK inhibitors neutralizes the paradoxical activation of the ERK pathway induced by BRAF inhibitors, modestly improving response rate and extending tumor control [23]. However, resistance and tumor recurrence remain inevitable. For the development of more efficacious therapeutics for BRAF-mutant tumors, a blockade of not only BRAF’s catalytic activity, but also its non-catalytic functions, is needed.

Hijacking the ubiquitin proteasome system via heterobifunctional small molecule compounds is an emerging pharmaceutical strategy aiming to selectively remove disease-causing proteins in affected cells. These small molecule degraders, also known as proteolysis-targeting chimeras (PROTACs), consist of a chemical tag that binds to a target protein of interest, connected by a linker to a second tag that binds to a cellular E3 ubiquitin ligase. By simultaneously binding to a target and an E3 ligase, the PROTAC stimulates the ubiquitination and subsequent degradation of the target by the proteasome. Targeted degradation of the protein of interest not only compromises the catalytic activities of these proteins but also removes their scaffolding and other non-catalytic functions, providing unique advantages over inhibitors [24,25].

A growing list of disease-relevant proteins, mostly implicated in oncology, has been targeted by PROTAC molecules [26,27,28,29,30,31].

**Figure 1:**
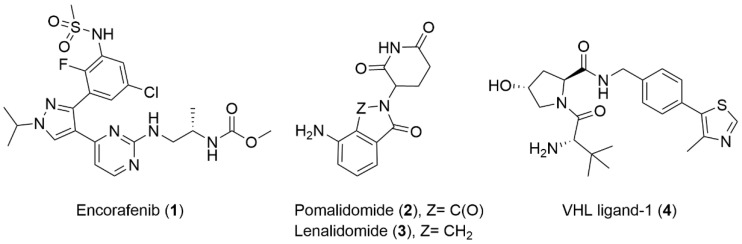
Chemical structures of encorafenib, pomalidomide, lenalidomide and VHL ligand-1.

With the aim of inducing BRAF inhibition and degradation, in this study we designed a series of PROTACs built upon the scaffold of the approved drug encorafenib (**1**, Figure 1). The solvent-exposed terminal group in encorafenib was linked to pomalidomide (**2**, Figure 1), a widely employed cereblon (CRBN) binder, through spacers endowed with different lengths and flexibility. Two additional molecules containing encorafenib bound to lenalidomide (**3**, Figure 1), another glutarimide-based CRBN binder, and to the VHL ligand-1 (4*R*)-3-methyl-L-valyl-4-hydroxy-N-[[4-(4-methyl-5-thiazolyl)phenyl]methyl]-L-prolinamide (**4**, Figure 1), were also designed.

In this study, we report the synthesis and in vitro evaluation of BRAF^V600E^ inhibition together with the antiproliferative activity in melanoma and colon cancer cell lines A375 and Colo205 of the synthesized compounds. The ability of selected compounds to induce degradation of the target protein is also discussed. Finally, we illustrate the computational studies performed to explain the lack of target degradation in comparison with compound P5B [24], a recently disclosed BRAF^V600E^ degrader.

## 2. Results and Discussion

### 2.1. Chemistry

**Figure 2:**
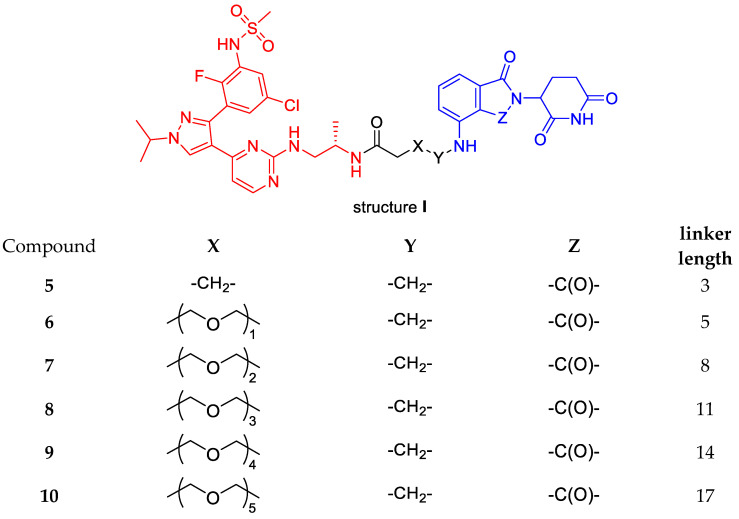

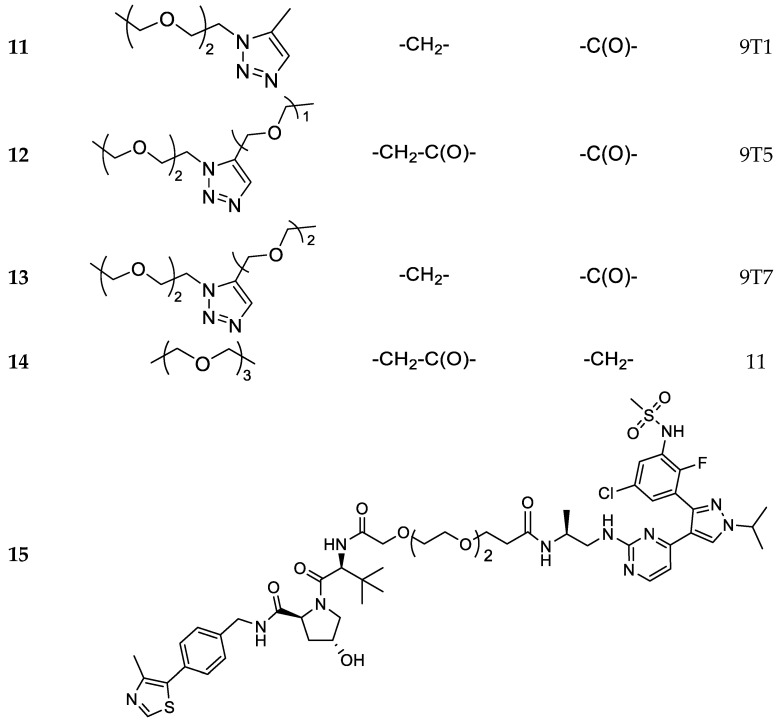
Structure of encorafenib-based PROTACs synthesized.

In order to obtain compounds bearing the BRAF^V600E^ ligand and the CRBN ligand at different spatial positions, the PEG-derived linkers were selected to cover a wide range, spanning from three to seventeen atoms (compounds **5**–**10**, Figure 2). To study the possible influence of the flexibility in the formation of the ternary complex between the protein of interest (BRAF^V600E^) and the ligase, compounds **11**–**13**, bearing a 1,5-disubstituted triazole ring at different points of the linker, were also synthesized. Finally, in compound **8**, the scaffold of pomalidomide was replaced with either lenalidomide (compound **14**) or VHL ligand-1 (compound **15**) to assess the behavior of different ligase-targeting groups.

**Figure 3:**
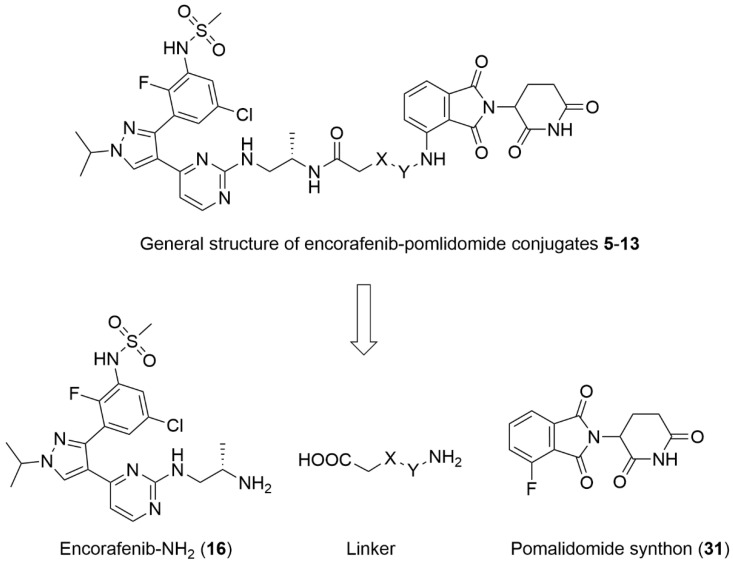
General strategy for the synthesis of encorafenib conjugates.

The potential PROTACs bearing pomalidomide were synthesized following the general strategy, illustrated in Figure 3. According to our approach, in order to insert the desired linkers into the structure of final PROTACs, we needed linker synthons containing a free terminal amino group on one side and a carboxylic acid group on the other side of the linker chain. We envisaged that the amino group could be first inserted into the phthalimide scaffold of pomalidomide through the nucleophilic substitution of the fluorine atom in 2-(2,6-dioxopiperidin-3-yl)-4-fluoroisoindoline-1,3-dione (**31**); then, the carboxylic group could be used to generate an amide bond with **16** to afford the designed compounds **5**–**14**.

**Scheme 1:**
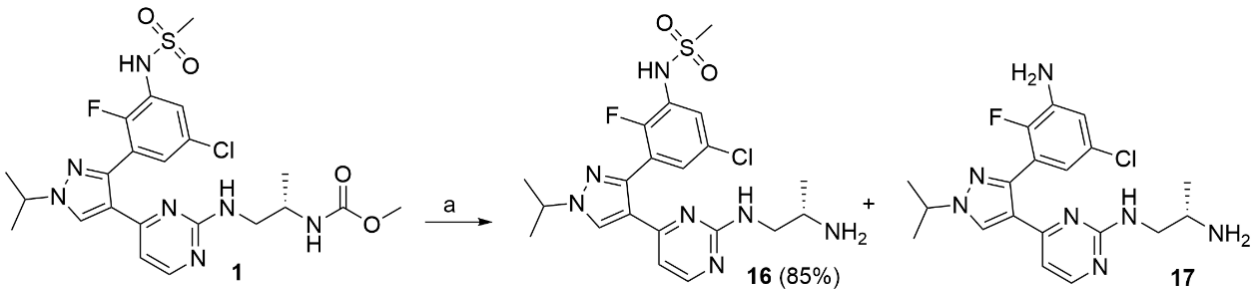
Reagents and conditions: (**a**) KOH 3M, EtOH/H_2_O 1:1, 78 °C, 7 h.

To synthesize **16**, we used encorafenib (**1**) as the starting material. The methyl carbamate in **1** was hydrolyzed to obtain the free amine **16**. Different acidic or basic conditions for the hydrolysis of **1** were tested; in particular, aqueous 3M KOH solution in EtOH (1:1) at 78 °C, aqueous 6M LiOH in 1,4-dioxane (1:1), or HCl 37% in 1,4-dioxane (1:1) at 100 °C were employed. All of the reaction conditions led to the formation of the desired primary amine **16** together with compound **17**, obtained from the concomitant hydrolysis of the methyl sulfonamide moiety. Among the tested conditions, the use of aqueous KOH in EtOH proved the most convenient protocol, affording 85% yield of **16** isolated after flash chromatography (Scheme 1 and Figure S1).

**Scheme 2:**
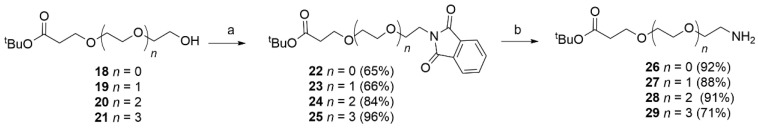
Reagents and conditions: (**a**) PPh_3_, DIAD, Phthalimide, THF, rt, 12 h; (**b**) NH_2_NH_2_•2 H_2_O, EtOH, rt, 12 h.

Having in hand a sufficient amount of the amine **16**, we turned our attention to the synthesis of the different linkers. To obtain the linkers **26**–**29**, we started from *tert*-butyl carboxylates **18**–**21**, produced from commercially available diols according to the literature [32,33,34]. These alcohols were converted into the corresponding phthalimides **22**–**25** using the Mitsunobu procedure. Finally, hydrazine-mediated hydrolysis of the phthalimide portion afforded the desired **26**–**29** in 60–76% yields over two steps (Scheme 2).

**Scheme 3:**
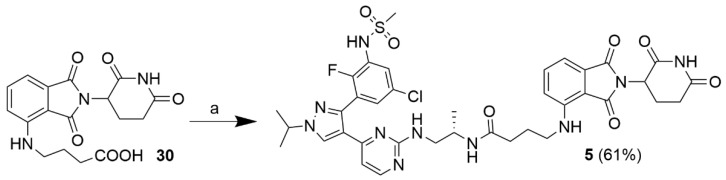
Reagents and conditions: (**a**) HATU, DIPEA, DMF, **16**, rt, 16 h.

To synthesize compound **5**, bearing a four-carbon atom spacer between encorafenib and pomalidomide, 4-((2-(2,6-dioxopiperidin-3-yl)-1,3-dioxoisoindolin-4-yl)amino)butanoic acid (**30**) [35] was coupled with **16**, using 1-[*Bis*(dimethylamino)methylene]-1H-1,2,3-triazolo [4,5-b]pyridinium 3-oxid hexafluorophosphate (HATU) as the activating reagent, DIPEA as the base and DMF as the solvent to afford 61% of the desired **5** after chromatography (Scheme 3).

**Scheme 4:**
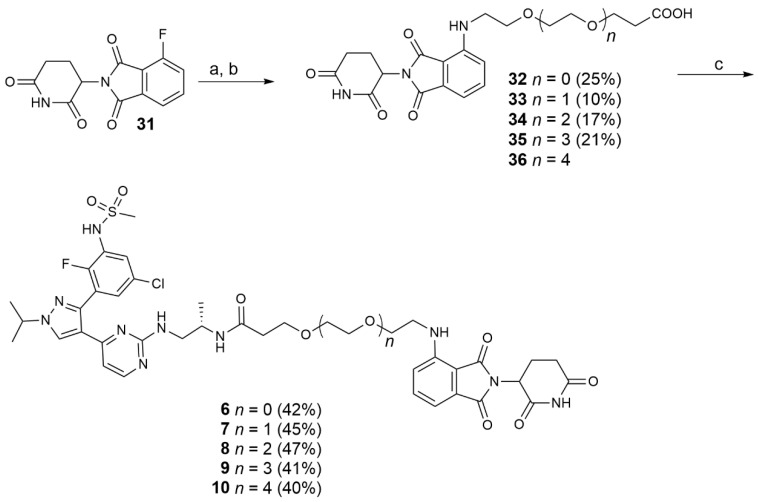
Reagents and conditions: (**a**) DIPEA, DMF, 90 °C, amines **26**–**29**, 16 h; (**b**) TFA 10% in CH_2_Cl_2_, rt, 12 h; (**c**) HOBt, HBTU or HATU, DIPEA, DMF, **16**, rt, 16 h.

The synthesis of the final compounds **6**–**10**, bearing PEG linkers of different lengths, is depicted in Scheme 4. The 2-(2,6-dioxopiperidin-3-yl)-4-fluoroisoindoline-1,3-dione (**31**) [36], used as pomalidomide precursor, was reacted with a stoichiometric amount of the appropriate amines **26**–**29** in DMF, at 90 °C in the presence of DIPEA. Hydrolysis of the *tert*-butyl protected intermediates was performed with 10% TFA in CH_2_Cl_2_ to obtain the carboxylic acids **32**–**35**, while compound **36** was commercially available. Coupling of free acids **32**–**36** with **16** was conducted using Hydroxybenzotriazole (HOBt)/N,N,N′,N′-Tetramethyl-O-(1H-benzotriazol-1-yl)uronium hexafluorophosphate (HBTU) or HATU as the preferred coupling reagent systems, DIPEA as the base and DMF as the solvent. Derivatives **6**–**10** were purified by flash chromatography or semi-preparative RP-HPLC (CH_3_CN:H_2_O 45:55 + 0.1% TFA) to afford the final compounds in >95% purity (HPLC). Compounds **6**–**10** were characterized by MS, ^1^H- and ^13^C-NMR.

**Scheme 5:**
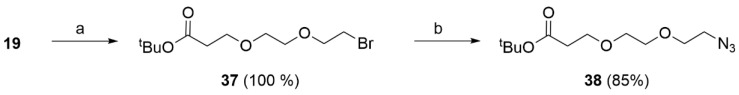
Reagents and conditions: (**a**) PPh_3_, CBr_4_, THF, rt, 12 h; (**b**) NaN_3_, DMF/H_2_O 5:1, 70 °C, 16 h.

To synthesize the triazole-containing final compounds **11**–**13**, the intermediate **38** (Scheme 5), bearing an azido terminal group, was sought. The *tert*-butyl 3-(2-(2-hydroxyethoxy)ethoxy)propanoate (**19**) was identified as a good starting material for the production of **38**. Compound **19** was first converted into the bromide **37** using the PPh_3_ and CBr_4_, then the obtained bromo-derivative was reacted with NaN_3_ to give the desired azide **38** in 85% overall yield (Scheme 5).

**Scheme 6:**
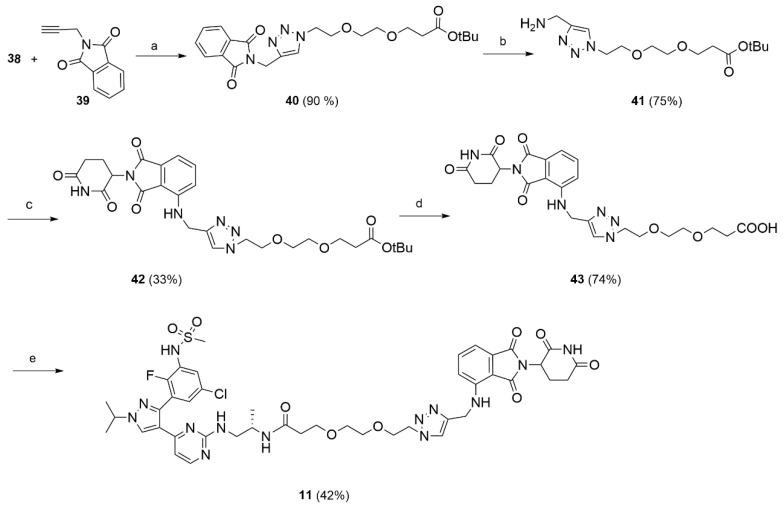
Reagents and conditions: (**a**) CuSO_4_, sodium ascorbate, t-BuOH/H_2_O 1:1, 90 °C, 3 h; (**b**) NH_2_NH_2_•2 H_2_O, EtOH, rt, 12 h; (**c**) **31**, DIPEA, DMF, 90 °C, 16 h; (**d**) TFA 10% in DCM, rt, 12 h; (**e**) HATU, DIPEA, DMF, **16**, rt, 16 h.

**Scheme 7:**
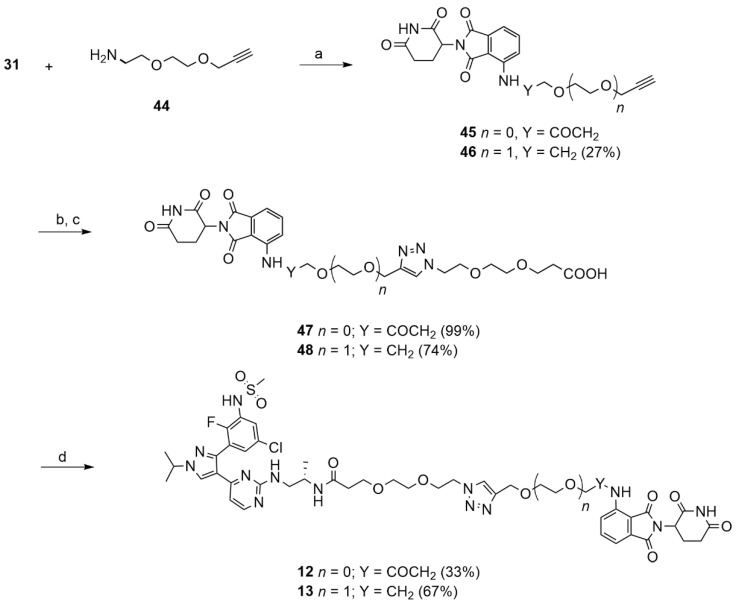
Reagents and conditions: (**a**) DIPEA, DMF, 90 °C, 16 h; (**b**) **38**, CuSO_4_, sodium ascorbate, t-BuOH/H_2_O 1:1, 90 °C, 3 h; (**c**) TFA 10% in DCM, rt, 12 h; (**d**) HATU, DIPEA, DMF, **16**, rt, 16 h.

The synthetic routes employed for the synthesis of compounds **11**–**13**, bearing a triazole moiety in different positions of the linker, are depicted in Scheme 6 and Scheme 7. For the synthesis of compound **11** (Scheme 6), the triazole-containing linker **41** was synthesized using a copper (I)-catalyzed alkyne-azide cycloaddition (CuAAC) reaction between the synthesized azide **38** and the commercially available N-propargyl phthalimide (**39**), followed by treatment with hydrazine hydrate of the intermediate **40**. The obtained free amine **41** was reacted with **31** to afford the *tert*-butyl protected carboxylic acid **42**. This intermediate was then converted to the free acid **43** and coupled to **16** using the procedures reported above, to afford the final compound **11**.

The synthesis of **12** and **13** is illustrated in Scheme 7. The intermediate terminal alkyne N-(2-(2,6-dioxopiperidin-3-yl)-1,3-dioxoisoindolin-4-yl)-3-(prop-2-yn-1-yloxy)propenamide (**45**) was commercially available, while **46** was obtained through the nucleophilic substitution of 2-(2-(prop-2-yn-1-yloxy)ethoxy)ethan-1-amine (**44**) on fluoro-derivative **31** at 90 °C in DMF. The intermediates **45** and **46** were then reacted with the previously synthesized azide **38** in a CuAAC reaction to efficiently furnish, after deprotection in acidic medium, the 1,4-disubstituted-1,2,3-triazole derivatives **47**–**48** bearing a free carboxylic acid group. HATU-mediated coupling of the generated acids with **16** furnished the desired final compounds **12** and **13**.

**Scheme 8:**
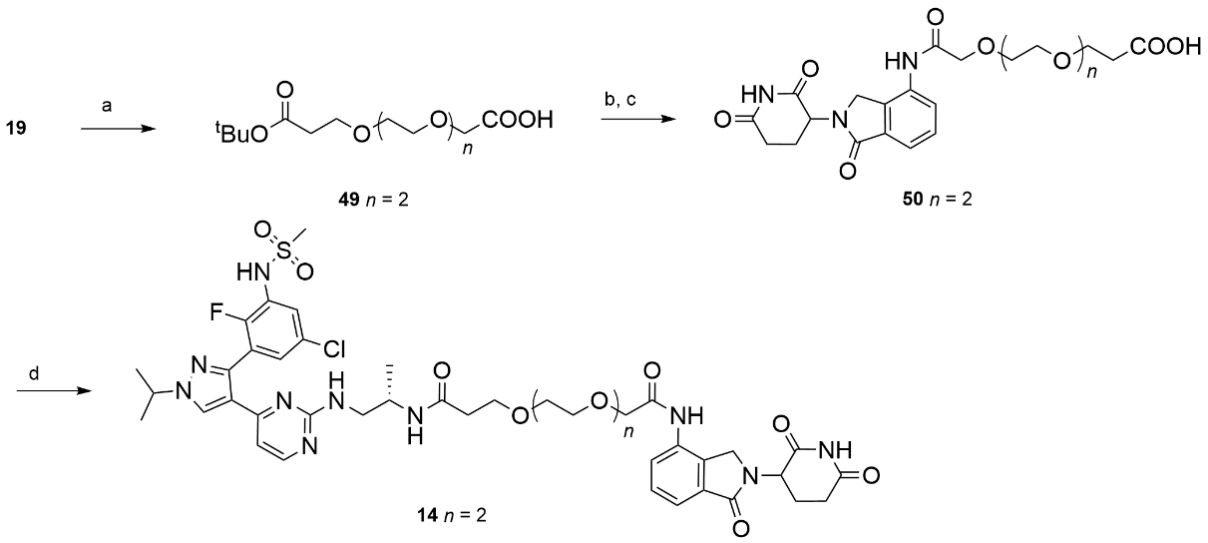
Reagents and conditions: (**a**) BAIB, TEMPO ACN/H_2_O 1:1, 90 °C, 3 h; (**b**) HATU, DIPEA, DMF, **3**, rt, 16 h; (**c**) TFA 10% in DCM, rt, 12 h; (**d**) HATU, DIPEA, DMF, **16**, rt, 16 h.

Compound **14** was obtained by conjugation of **16** to lenalidomide (**3**), as depicted in Scheme 8. In order to synthesize **14**, we used a 12-atom linker (**49**) to join the two selected chemical scaffolds. Linker **49** contains a free carboxyl group at one end and a *tert*-butyl protected carboxylic acid at the other end. The synthesis of **49** was performed by oxidation of the terminal hydroxyl group in **19** by using a (2,2,6,6-Tetramethylpiperidin-1-yl)oxyl (TEMPO)/(Diacetoxyiodo)benzene BAIB oxidating system [37]. The obtained intermediate was then coupled with **3** using HATU to promote the formation of the amide bond. After chromatographic purification, the intermediate *tert*-butyl protected carboxylate was deprotected, and the free acid **50** was coupled to **16** to afford the designed **14**.

**Scheme 9:**
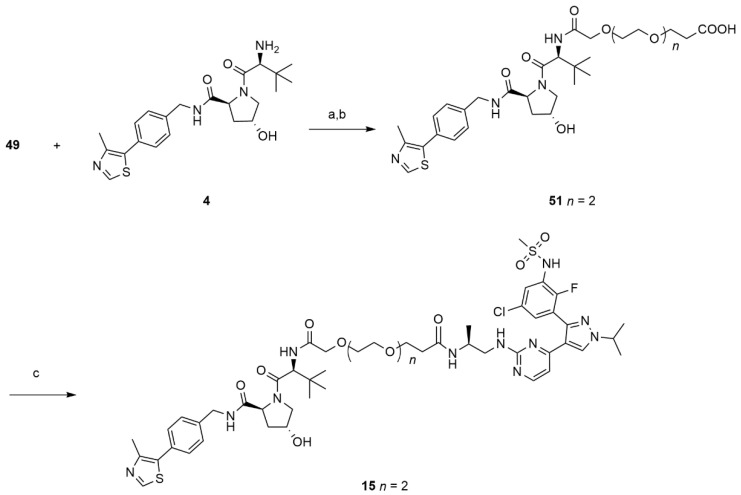
Reagents and conditions: (**a**) HATU, DIPEA, DMF, **4**, rt, 16 h; (**b**) TFA 10% in DCM, rt, 12 h; (**c**) HATU, DIPEA, DMF, **16**, rt, 16 h.

The same strategy was employed for the synthesis of **15**, which bears the VHL-ligand (2S,4R)-1-((S)-2-Amino-3,3-dimethylbutanoyl)-4-hydroxy-N-(4-(4-methylthiazol-5-yl)benzyl)pyrrolidine-2-carboxamide (**4**) as the terminal VHL-engaging portion (Scheme 9).

**Scheme 10:**
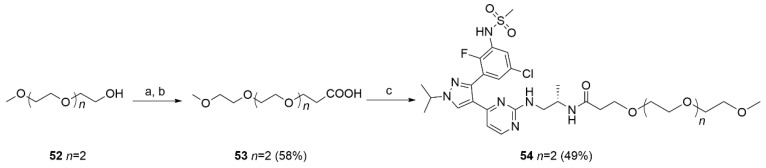
Reagents and conditions: (**a**) NaH, THF, 60 °C, tert-butyl acrylate 48 h; (**b**) TFA 10% in DCM, rt, 12 h; (**c**) HATU, DIPEA, DMF, **16**, rt, 16 h.

Finally, the reference compound **54**, lacking the CRBN ligand, was accomplished according to the route reported in Scheme 10. Encorafenib derivative **54**, bearing a 13-atom PEG-type linker, was obtained through the reaction of 2-(2-(2-methoxyethoxy)ethoxy)ethan-1-ol (**52**) with *tert*-butyl acrylate, followed by hydrolysis of the *tert*-butyl ester in acidic medium (TFA 10%), to furnish the acid **53**. Finally, HATU-mediated formation of the amide bond allowed the coupling with derivative **16**. Using this route, it was possible to obtain the desired **54** in 28% overall yield.

### 2.2. Biological Evaluation

#### 2.2.1. BRAF^V600E^ Kinase Activity

**Figure 4:**
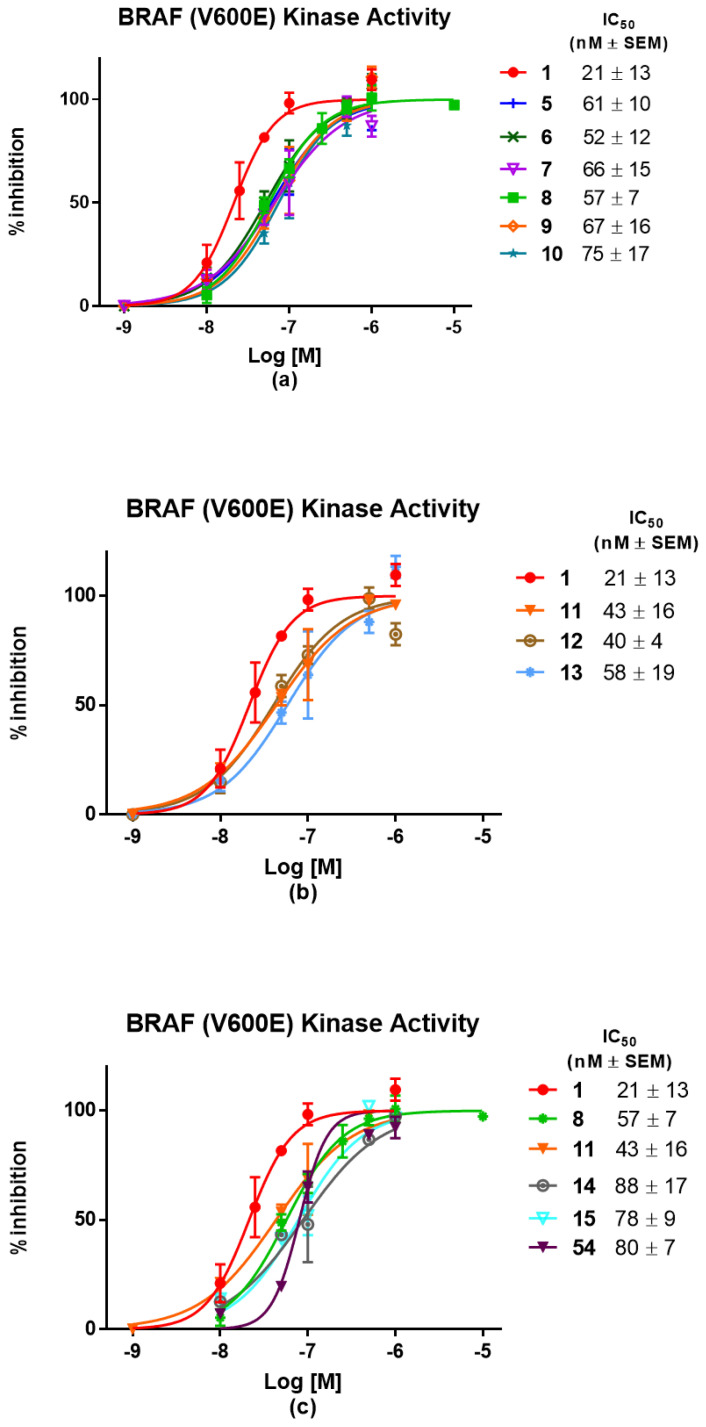
Concentration–response curves for isolated BRAF^V600E^ inhibition for compounds **5**–**15**, **54** and encorafenib (**1**). (**a**) Comparison of compounds with flexible PEG-type linkers of different length; (**b**) Comparison of compounds with triazole ring-type linker of different length; (**c**) comparison of compounds with different E3 ligase ligands of similar length or lacking the E3-ligase binder (**54**). Data are the results of three different experiments run in duplicate.

The synthesized compounds **5**–**15** and the reference compound **54** were characterized to verify their ability to inhibit the activity of isolated BRAF^V600E^ using a fluorescent assay. The obtained results are reported in Figure 4. All the synthesized compounds maintained the ability to inhibit the kinase activity of mutated BRAF with IC_50_ values in the 40–88 nM range, showing no statistical difference in their activity. In the same assay, encorafenib (**1**) was the most active compound, showing an IC_50_ of 21 ± 13 nM. The synthesized conjugates were 2–4-fold less active than the parent compound **1**. The examination of the concentration–response curves (Figure 4a–c) shows that all the compounds behave quite similarly toward BRAF^V600E^ inhibition. The length of the PEG-type linker used (compounds **5**–**10**, Figure 4a) does not have an influence on the ability of compounds to inhibit BRAF^V600E^; the same applies for compounds containing a triazole ring at different position of the linker (compounds **11**–**13**, Figure 4b). Finally, the comparison between compounds obtained by joining encorafenib (**1**) with a simple PEG-type linker (compound **54**) or the different E3-ligase binders, in particular pomalidomide (compound **11**), lenalidomide (compound **14**) or VHL-ligand-1 (compound **15**), showed that compounds are endowed with similar potencies (Figure 4c). This observation underlines that the encorafenib substructure is still able to bind the target protein when the solvent-exposed moiety is modulated through the introduction of large molecular portions of different chemical composition.

#### 2.2.2. Stability Assays

**Figure 5:**
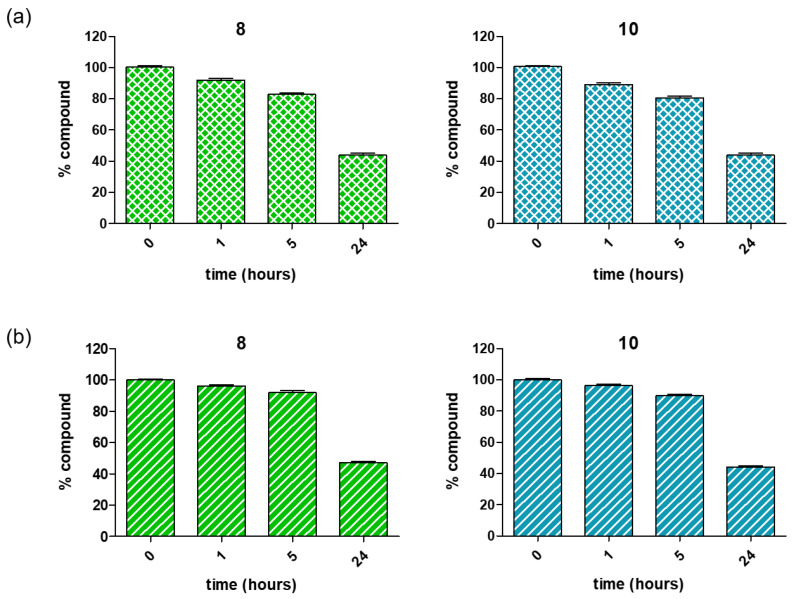
Stability of compounds **8** and **10** over 24 h: (**a**) in RPMI medium, (**b**) in human serum.

Glutarimide derivatives pomalidomide (**2**) and lenalidomide (**3**) are widely used in the generation of PROTACs, owing to their ability to bind to CRBN. As it is known, their intrinsic hydrolytic instability could limit their biological activity [38]. Therefore, the stability of selected compounds in cell medium (RPMI) and human serum was checked before performing antiproliferative assays in tumor cell lines. Compounds **8** and **10** were chosen as representative examples of the series of synthesized encorafenib conjugates. In our experiments, compounds **8** and **10** showed satisfactory stability with a half-life of 21 h and 19 h in RPMI, and of 24 h and 23 h in human serum, respectively (Figure 5). We also checked the cell permeability of compound **8**, used as a model compound, in Colo205 cells, demonstrating its ability to reach the cytosolic compartment, as expected (Table S1).

#### 2.2.3. Antiproliferative Activity

Based on the previous preliminary tests, we sought to investigate the ability of synthesized compounds **5**–**15** and reference **1** in inhibiting the proliferation of BRAF^V600E^ mutant cancer cells. A first screening was conducted in A375 melanoma cell lines (Table 1, Figure S2). Encorafenib was employed for comparison. We found that all the synthesized compounds were able to inhibit the proliferation of A375 cells, even with a lower potency compared with encorafenib (**1**).

**Figure 6:**
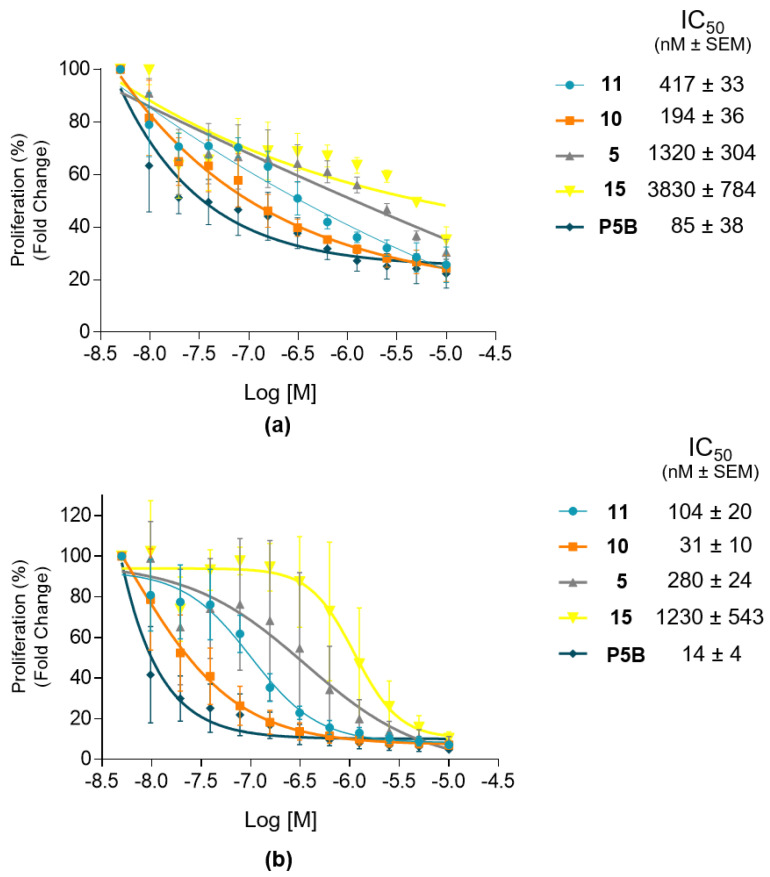
Concentration–response curves for antiproliferative activity of compounds **5**, **10**, **11**, **15** and reference P5B. (**a**) Antiproliferative activity and IC_50_ values of selected compounds in A375 cell line; (**b**) Antiproliferative activity and IC_50_ values of selected compounds in Colo205 cell line. Data are the mean ± SEM of three independent experiments run in triplicate.

The ability of compounds **5**, **10**, **11**, **15**–selected based on their chemical diversity–to inhibit cell proliferation was then tested both in A375 and in Colo205 cancer cell lines. In this new set of experiments, we decided to compare the antiproliferative effects of our compounds with that of a recently published compound, named P5B (Figure S3), which is highly efficient in inducing BRAF^V600E^ degradation in A375 cells [24]. P5B was obtained by joining the BRAF inhibitor BI 882370 to pomalidomide through a five PEG unit spacer (Figure S3). In compound P5B, the linker length was of 17 atoms, the same length of the spacer used to generate our compound **10**. A375 and Colo205 cells were treated for 72 h with compounds **5**, **10**, **11**, **15**, and P5B, and the antiproliferative activity was measured by a luminescent ATP-based assay, which determines the number of viable cells based on their metabolic activity. The concentration–response curves obtained, and IC_50_ values are reported in Figure 6. Among the synthesized compounds, **10** and **11** were the most potent in inhibiting the proliferation of BRAF mutant cell lines, with IC_50s_ in the nanomolar range; meanwhile, compound **15**, bearing the VHL ligand-1 at one end, was active only at micromolar concentration. However, P5B was more efficient in inhibiting tumor cell viability in comparison with the other selected compounds.

**Figure 7:**
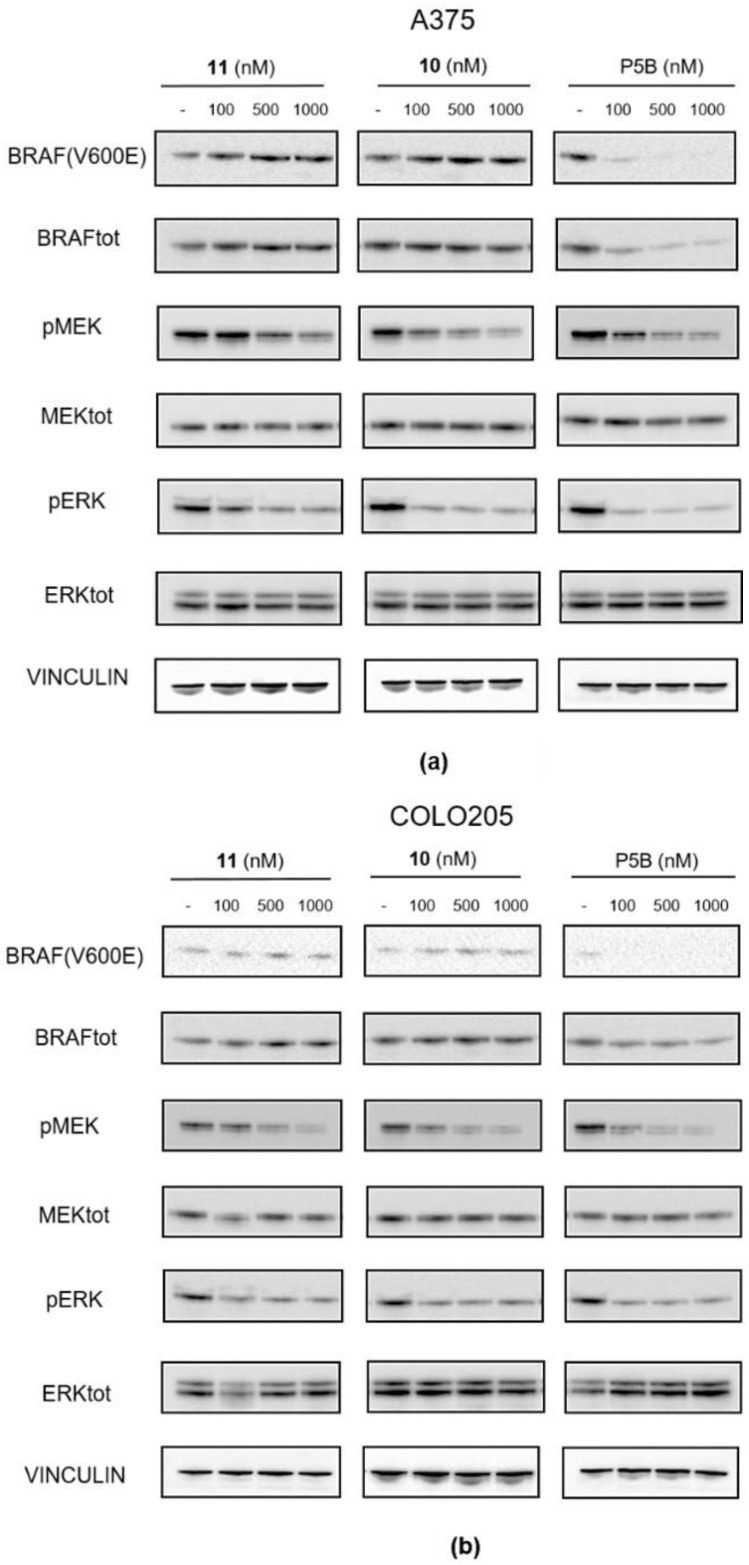
Western blot (WB) analysis of BRAF signaling in (**a**) A375 and (**b**) Colo205 cell lines treated with different concentrations of compounds **10**, **11** and P5B. The reported WB is one representative analysis of three different experiments.

Finally, we investigated the capability of the most antiproliferative compounds in interfering with BRAF pathway, favoring target degradation. To this end, Western blot analysis was conducted 24 h after incubation with **10** and **11** in both A375 and Colo205 cell lines. Compounds **10** and **11** proved able to decrease phosphorylation of MEK and ERK in both A375 and Colo205 cell lines, similarly to the reference compound P5B (Figure 7a,b). This observation was consistent with their ability to inhibit BRAF^V600E^. Surprisingly, after 24 h treatment of either A375 or Colo205 cells with encorafenib-pomalidomide conjugates **10** and **11** at 100–500 nM, both the synthesized compounds were not able to induce BRAF^V600E^ degradation. On the contrary, P5B efficiently induced BRAF^V600E^ degradation in the same range of concentration in the tested tumor cell lines (Figure 7a,b). When **10** and **11** were tested at higher concentration (up to 10 µM), no degradation of the target protein was in any way observed (Figure S4).

We investigated the possible reasons underlying the failure of our prototype compounds in inducing BRAF^V600E^ degradation. We reasoned that the synthesized compounds: (i) inhibit isolated BRAF^V600E^ kinase activity; (ii) penetrate the cell and inhibit the signaling of BRAF^V600E^; (iii) are stable enough in the cell environment to bring about the formation of the ternary complex between the protein target and the E3-ligase. Taking into account the above observations and the structural similarity of compound **10** and P5B, a possible explanation for the lack of target protein degradation could be that the compound, once inside the cell, can bind to the kinase target but is not able to engage the E3-ligase. To investigate this possibility, we performed in-depth molecular modeling studies of compound **10** compared with P5B in BRAF^V600E^.

#### 2.2.4. Computational Studies

##### Molecular Docking

**Figure 8:**
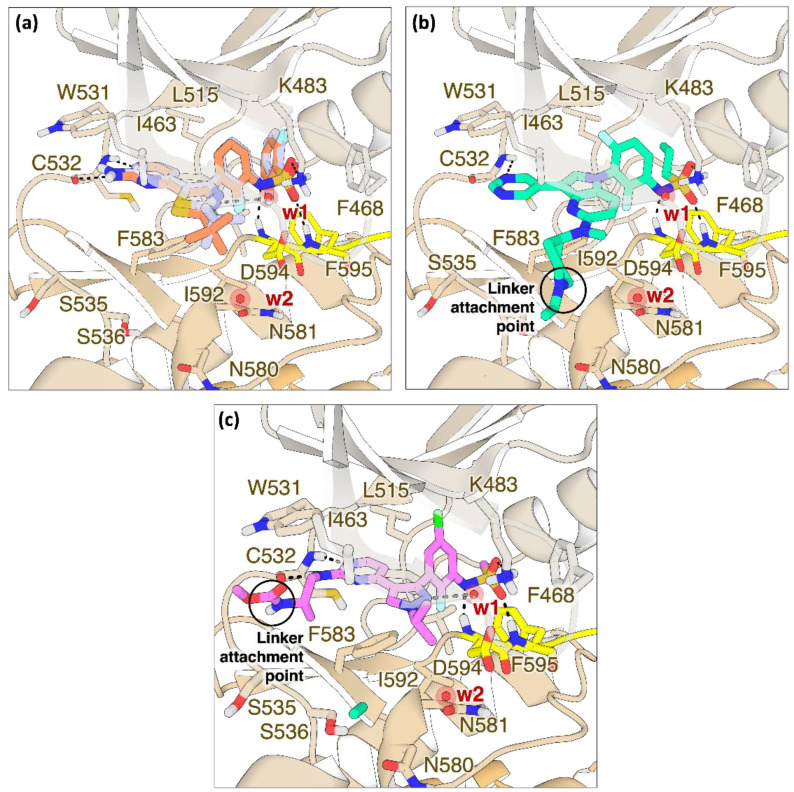
Self-docking of dabrafenib superposed to dabrafenib crystal pose (**a**) and docking poses of (**b**) BI 882730 and (**c**) encorafenib in BRAF^V600E^. In (**a**), the dabrafenib docking pose is colored lilac, while X-ray binding pose (PDB ID: 5CSW) is colored orange. In (**b**), BI 882730 (green) and in (**c**) encorafenib (pink) ligands are depicted as sticks; the linker attachment point for obtaining P5B and **10** is encircled and labelled. Waters retained in docking are shown as red spheres and labelled as ‘w1’ and ‘w2’, hydrogen bonds are shown as dashed black lines and non-polar hydrogens are omitted for clarity. Protein is represented as cartoon and colored in beige, binding site residues are shown as sticks and labelled with one letter code and the DFG loop is highlighted in yellow.

Docking studies were first performed to investigate the binding mode of warheads BI 882370 and encorafenib in BRAF^V600E^, as the kinase inhibitor moiety is the main discriminant between P5B and conjugate **10**. Both BI 882370 and encorafenib are type I½ RAF kinase inhibitors, and stabilize a DFG_in_ and αC-helix_out_ conformation of BRAF [3]. While no experimental structure of encorafenib in BRAF is available, two X-ray structures of BI 882370 in BRAF can be found in the PDB (IDs: 5CSX, 6UUO). The structure of BI 882370-bound BRAF in PDB ID 5CSX reports an αC-helix_out,_ but also DFG_out_ conformation, with the DFG_out_ being the result of a crystallization artifact, as already discussed elsewhere [24]. In contrast, PDB ID 6UUO shows a DFG_in_ and an αC-helix_out_ conformation of BRAF, but with a low resolution (3.29 Å), and P-loop and activation loop (A-loop) disordered. As a consequence, PDB IDs 5CSX and 6UUO were discarded for modelling BRAF^V600E^ in complex with either P5B or **10**, while BRAF in complex with dabrafenib, another type of I½ BRAF inhibitor (PDB ID: 5CSW, resolution 2.66 Å) was selected (see Methods section). The resulting BRAF^V600E^ model showed a DFG_in_ loop, an αC-helix_out_ and an unfolded A-loop, in accordance with the conformation stabilized by type I½ RAF kinase inhibitors BI 882370 and encorafenib [3]. After self-docking of dabrafenib in BRAF^V600E^ (RMSD_heavy_ = 0.563 Å), docking of BI 882370 and encorafenib was performed (Figure 8). Docking poses of BI 882370 and encorafenib trace the polar interactions of dabrafenib observed in 5CSW X-ray structure, and involve hydrogen bonds between residues Asp594 and Phe595 (DFG segment), Lys483 and the sulfonylamide moiety of the inhibitors. Another key hydrogen bond involves the nitrogen of Cys532 backbone, and the pyrimidinyl substituent of both BI 882370 and encorafenib. Noticeably, two main differences can be spotted between BI 882370 and encorafenib at the docking pose level; (i) the 2-aminopyrimidin-4-yl moiety in encorafenib can establish a typical 1–3 hydrogen bond with the backbone of Cys532 in the hinge region, and (ii) the linker attachment point is oriented differently with respect to the overall binding mode.

##### Molecular Dynamics

Next, we planned to investigate the stability of P5B and **10**, bearing BI 882730 and encorafenib as the kinase ligands, respectively. Three replicas of unbiased molecular dynamics simulations (500 ns each) in explicit solvent were set up (see Methods section) for two systems: (i) BRAF^V600E^ in complex with P5B, and (ii) BRAF^V600E^ in complex with **10**, hereafter referred to as **BRAF:P5B** and **BRAF:10**, respectively. We monitored the behavior of the P5B and **10** conjugates during simulations, looking for differences at the binary complex level that might explain the lack of degradation activity shown by **10**. Intramolecular hydrogen bonds established along the MD production by P5B and **10** were monitored; however, no relevant discrepancies were recorded. Furthermore, the solvent exposure of the P5B and **10** conjugates was evaluated during the simulations as the solvent-accessible surface area (SASA); however, no significant difference was found across replicas (Figure S5).

**Figure 9:**
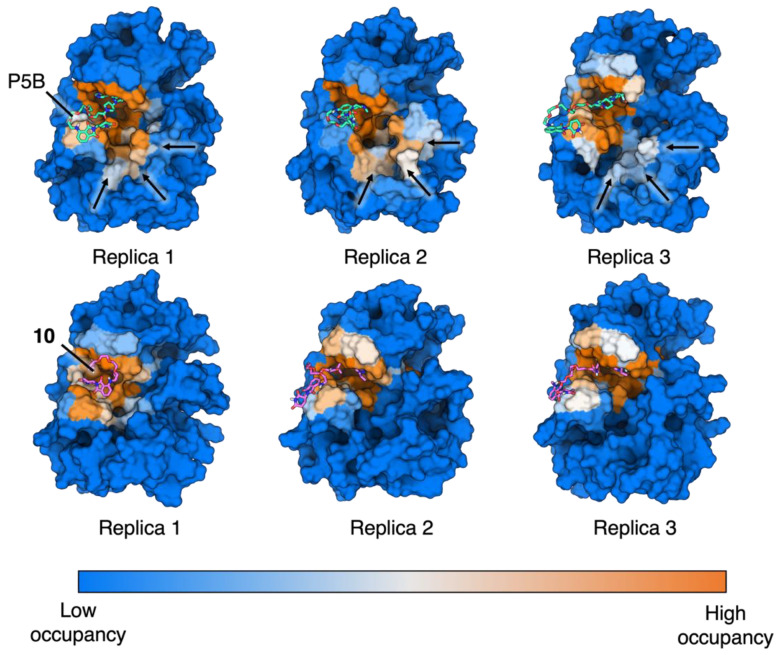
Occupancy of ligand–protein contacts along MD productions, mapped on the surface of BRAF. Occupancy is defined as the normalized number of MD frames in which atoms of the ligand are at a distance equal or lower than 4 Å from protein atoms. Occupancy is shown as a range of colors spanning from blue (low occupancy) to orange (high occupancy) areas. Black arrows highlight areas of BRAF^V600E^ which were accessed by P5B but not by **10**. Protein is represented as surface, ligand is depicted as sticks and labelled.

Through visual inspection of the MD trajectories (see Movies 1–6 in Supporting Info), a different dynamic behavior and interaction pattern of the linker and pomalidomide segment in P5B and **10** was observed. Notably, in P5B, the linker and pomalidomide moiety explored a larger surface of BRAF^V600E^, while the same segment in **10** was restricted to a narrower area of the binding cavity. Such differences in protein–ligand interactions were characterized by mapping the generic ligand–protein contacts (protein atoms distant 4 Å or less from ligand atoms) along MD replicas, and weighted according to their occupancy, i.e., persistence on the total MD frames (Figure 9). Black arrows in Figure 9 highlight a surface area of the carboxyterminal lobe of BRAF^V600E^ which was accessed by P5B but not by **10** in MD replicas. This area is lined by residues Lys578, Leu613, Ser614, Leu618, Trp619 and Leu654 (Figure S6).

Furthermore, the hydrogen bond pattern of P5B and **10** along MD simulations was analyzed, searching for possible polar contacts that may affect the linker conformation (Tables S2 and S3); overall, P5B showed a richer and more variable network of hydrogen bonds with respect to **10**. In **BRAF:P5B**, the linker orientation was stabilized by a persistent hydrogen bond between the 1-oxo-piperidinyl moiety and Asn580 in all replicas, while in **BRAF:10** the linker attachment point was constrained in its position by the firm hydrogen bond between the pyrimidinyl ring of **10** and Cys532.

In this section, we analyzed differences between P5B and **10** at the binary complex level that may explain their efficacy or inefficacy as BRAF^V600E^ degraders. By observing MD simulations and analyses, we gathered no data supporting unfavorable interactions between **10** and BRAF^V600E^ that could hinder the recruitment of the E3 ligase CRBN with respect to P5B. A relevant feature emerging from our MD replicas regards the differences in the protein area of BRAF^V600E^ accessed by the linker and pomalidomide segments in P5B and **10** conjugates. Indeed, the linker and pomalidomide moiety in **10** are constrained near the binding site of BRAF^V600E^ by the linker attachment point on encorafenib and by the canonical hydrogen bond between the amino-pyrimidinyl moiety and the backbone of Cys532. On the contrary, the linker attachment point in P5B led to more degrees of freedom and could extend to an area of BRAF which is inaccessible to **10**. By comparing our results with other BRAF ligands reported in the literature for designing BRAF degraders, we noticed that effective BRAF degraders (BI 882730-, vemurafenib- or PLX-based conjugates) orient the linker attachment point towards one exit vector, while in weakly active or inactive BRAF degraders containing dabrafenib (or encorafenib, in this work) the exit vector for the linker is directed differently [24,25,39,40]. Considering this agreement with BRAF degraders reported by the literature, we speculate that the inactivity of **10**, and of other ineffective BRAF degraders exploiting the same exit vector, might not be explained by focusing only on the formation of the binary or ternary complex, but by taking into account several aspects of protein target degradation.

A key factor to be addressed when rationalizing the activity of PROTACs regards the productivity of the ternary complex, i.e., the effective ubiquitination of the target protein. In this context, it has been largely demonstrated that the formation of an energetically stable ternary complex does not correlate with efficient protein target degradation, while an efficient ubiquitination typically leads to successful protein target degradation [41]. Overall, we may argue that in the case of both P5B and **10**, a ternary complex could be formed; however, while P5B might lead to the formation of a productive ternary complex, **10** might lead to an unproductive ternary complex. Furthermore, the productive protein–protein interactions (PPIs) may be formed at the level of the BRAF^V600E^ surface explored by P5B in our MD simulations, which, however, could not be reached by **10**. Further work would be required to test such a hypothesis, eventually clarifying the structure of productive BRAF^V600E^/inhibitor/E3 ligase ternary complexes by coupling different experimental and in silico methods. Indeed, a recent paper by Eron et al. reports that PPIs stabilized by PROTACs can still be very dynamic, and X-ray structures of ternary complexes can also not correspond to a biologically relevant conformation, as they only represent a ‘crystallization snapshot’ of a highly dynamic interaction [42]. Demonstrating the efficacy of combining in silico methods with experimental data for studying the dynamics of PPIs in PROTACs design, a freshly published work by Dixon et al. was able to eventually predict the structural basis of targeted protein degradation [43]. In this work, the authors showed that productive ternary complexes, modelled in the context of the full Cullin-RING ligase, are characterized by the proximity of lysine residues of the target protein to the E2 ligase.

## 3. Materials and Methods

### 3.1. Chemistry

All the reactions were monitored by thin-layer chromatography (TLC) on Merck 60 F254 (0.25 mm) plates, which were visualized by UV inspection (254 nm) and/or by spraying KMnO_4_ (0.5 g in 100 mL 0.1 N NaOH). Na_2_SO_4_ was used as drying agent for the organic phases. Flash chromatography (FC) purifications were performed using silica gel Merck with 60 mesh particles. Unless otherwise specified, all reagents were used as received without further purification. Dichloromethane was dried over P_2_O_5_ and freshly distilled under nitrogen prior to use. DMF was stored over 3Å molecular sieves. Anhydrous THF was freshly distilled under nitrogen from Na/benzophenone ketyl. ^1^H and ^13^C-NMR spectra were registered on JEOL ECZR600 spectrometer, at 600 and 151 MHz. Coupling constants (J) are given in Hertz (Hz) and chemical shifts (δ) are given in ppm, calibrated to solvent signal as internal standard. The following abbreviations are used to describe multiplicities: s = singlet, d = doublet, t = triplet, q = quadruplet, m = multiplet and br = broad signal. The following abbreviation is used to identify the exact proton: ArH = aromatic proton. ESI-mass spectra were recorded on a Waters Micromass Quattro Micro equipped with an ESI source. Semi-preparative HPLC purifications were carried out on a Varian Pro-Star 210 chromatograph equipped with a variable wavelength detector (Prostar 325). The chromatography was performed using a 5 μM particle size Hibar LiChrosper C18 endcapped prepacked column (250 × 25 mm), with a flow rate of 20 mL/min; UV detection was performed at 226 and 240 nm. Samples of the mixture were dissolved (ca 0.5%) in mobile phase. The purity of target compounds was checked by RP-HPLC. Analyses were performed on an HP1100 chromatograph system (Agilent Technologies, Palo Alto, CA, USA) equipped with a quaternary pump (G1311A), a membrane degasser (G1379A) and a diode-array detector (DAD) (G1315B) integrated in the HP1100 system. Data analyses were processed by HP ChemStation system (Agilent Technologies). The analytical column was a LiChrospher^®^ 100 C18-e (250 × 4.6 mm, 5 µm) (Merck KGaA, 64271 Darmstadt, Germany) eluted with acetonitrile/0.1% TFA in a ratio depending on the characteristics of the compound. All compounds were dissolved in the mobile phase at a concentration of about 0.1 mg/mL and injected through a 20 μL loop. HPLC retention times (t_R_) were obtained at flow rates of 1.0 mL min^−1^, and the column effluent was monitored at 226, 254 and 300 nm, referenced against 800 nm. The purity of the compounds was evaluated as a percentage ratio between the areas of the main peak and of possible impurities at the three wavelengths, and also using DAD purity analysis of the chromatographic peak. The purity of all the target compounds was found to be ≥95% (Table S4 and Figure S7). Compound P5B was a kind gift of Prof. Frank Sicheri (Center for Molecular, Cell and Systems Biology, Lunenfeld-Tanenbaum Research Institute, Sinai Health System, Toronto, Ontario, Canada).

*Synthesis of target compounds (s)-n-(3-(4-(2-((2-aminopropyl)amino)pyrimidin-4-yl)-1-isopropyl-1H-pyrazol-3-yl)-5-chloro-2-fluorophenyl)methanesulfonamide* (**16**).

Encorafenib (**1**; 0.350 g; 6.49 mmol) was dissolved in ethanol (10 mL), and KOH 6M aqueous solution (10 mL) was added. The mixture was stirred at 80 °C for 7 h. The reaction mixture was neutralized with HCl 6M to pH 7 and the organic solvent evaporated under reduced pressure. The obtained precipitate was filtered, and the filtrate was extracted with THF (3 × 15 mL). The organic phases and the collected precipitate were combined and purified by flash chromatography using DCM/MeOH 9/1+ 0.1% NH_3_ as the eluent. Compound **16** was obtained in 85% yield as a white solid. ^1^H NMR (CD_3_OD) δ: 8.35 (s, 1H), 8.14 (d, *J =* 5.3 Hz, 1H), 7.51 (dd, *J =* 6.5, 2.5 Hz, 1H), 7.17 (s, 1H), 6.74 (s, 1H), 4.62–4.58 (m, 1H), 3.32 (s, 1H), 3.28 (dd, *J =* 3.1, 1.5 Hz, 1H), 2.96 (s, 3H), 1.55 (d, *J =* 6.7 Hz, 6H), 1.15 (s, 3H). ^13^C NMR (CD_3_OD) δ: 162.27 (s), 160.51 (s), 157.64 (s), 151.79 (d, *J =* 245.9 Hz), 143.27 (s), 129.61 (s), 128.57 (d, *J =* 2.7 Hz), 124.44 (d, *J =* 16.1 Hz), 123.63 (d, *J =* 12.6 Hz), 119.58 (s), 107.41 (s), 54.68 (s), 44.57 (s), 39.27 (s), 21.70 (s), 15.34 (s). MS (ESI^−^): *m*/*z* 480/482 [M-H]^−^; MS (ESI^+^): *m*/*z* 482/484 [MH^+^].

**General procedure for the synthesis of compounds 22–25.**

Phthalimide (1.4 eq), the appropriate alcohol (18–21) (1 eq) and triphenylphosphine (1.4 eq) were dissolved in dry THF (20 mL). After 15 min, DIAD (1.4 eq) was added dropwise at rt. After 12 h under stirring, EtOH (1 mL) was added to quench the reaction. The solvent was removed and the obtained residue purified by flash chromatography (PE/EtOAc 7.5/2.5 to 1/1) to afford the desired product. The obtained compounds were characterized by MS and used directly in the next step.

*Tert-butyl 3-(2-(1,3-dioxoisoindolin-2-yl)ethoxy)propanoate* (**22**).

Alcohol **18** (1.39 g, 7.30 mmol) was reacted with phthalimide (1.5 g, 0.01 mmol), PPh_3_ (2.68 g, 0.01 mol) and DIAD (2.06 g, 0.01 mol, 2 mL) in dry THF (25 mL) using the general procedure described above to obtain **22** (1.53 g, 4.79 mmol, 65%) as a white solid. MS (ESI^+^): *m*/*z* 320 [MH^+^].

*Tert-butyl 3-(2-(2-(1,3-dioxoisoindolin-2-yl)ethoxy)ethoxy)propanoate* (**23**).

Alcohol **19** (1.5 g, 6.40 mmol) was reacted with phthalimide (1.14 g, 7.80 mmol), PPh_3_ (2.04 g, 7.8 mmol) and DIAD (1.5 g, 7.8 mmol, 1.46 mL) in dry THF (25 mL) using the general procedure described above to obtain **23** (1.98 g, 5.46 mmol, 70%) as a white solid. MS (ESI^+^): *m*/*z* 364 [MH^+^].

*Tert-butyl 3-(2-(2-(2-(1,3-dioxoisoindolin-2-yl)ethoxy)ethoxy)ethoxy)propanoate* (**24**).

Alcohol **20** (0.694 g, 2.49 mmol) was reacted with phthalimide (0.513 g, 3.49 mmol) PPh_3_ (0.915 g, 3.49 mmol) and DIAD (0.706 g, 3.49 mmol, 0.687 mL) in dry THF (10 mL) using the general procedure reported above to obtained **24** (0.095 g, 9.54 mmol, 94%) as a white solid. MS (ESI^+^): *m*/*z* 408 [MH^+^].

*Tert-butyl 1-(1,3-dioxoisoindolin-2-yl)-3,6,9,12-tetraoxapentadecan-15-oate* (**25**).

Alcohol **21** (1.43 g, 4.44 mmol) was reacted with phthalimide (0.914 g, 6.21 mmol) PPh_3_ (2.25 g, 6.21 mmol) and DIAD (1.25 g, 6.21 mmol, 1.25 mL) in dry THF (20 mL) using the general procedure reported above to give **25** (1.92 g, 4.25 mmol, 96%) as a white solid. MS (ESI^+^): *m*/*z* 452 [MH^+^].

**General procedure for the synthesis of compounds 26–29 and 41.**

Phthalimide derivatives **22**–**25** and **40** were dissolved in EtOH and NH_2_NH_2_·2H_2_O was added at rt. The reaction mixture was stirred overnight. The solvent was removed and chloroform was added to the residue and stirred for 15 min. The formed precipitate was filtered off. The filtrate was concentrated under reduced pressure and the crude product was purified using flash chromatography (DCM/MeOH 95/5 to 90/10 + 0.1% NH_3_).

*Tert-butyl 3-(2-aminoethoxy)propanoate* (**26**).

Compound **22** (0.50 g, 1.55 mmol) was treated with NH_2_NH_2_·2H_2_O (0.39 g, 7.82 mmol) in EtOH (15 mL) using the general procedure reported above to obtain **26** (0.27 g, 1.44 mmol, 92%) as a colorless oil. MS (ESI^+^): *m*/*z* 452 [MH^+^]. ^1^H NMR (600 MHz, CDCl_3_) *δ* 3.69 (t, *J* = 6.5 Hz, 2H), 3.44 (t, *J* = 5.2 Hz, 2H), 2.83 (t, *J* = 5.2 Hz, 2H), 2.48 (t, *J* = 6.5 Hz, 2H), 1.44 (s, 9H), in agreement with that reported in the literature [32].

*Tert-butyl 3-(2-(2-aminoethoxy)ethoxy)propanoate* (**27**).

Compound **23** (0.70 g, 1.92 mmol) was treated with NH_2_NH_2_·2H_2_O (0.48 g, 9.63 mmol) in EtOH (15 mL) using the general procedure described above to obtain **27** (0.41 g, 1.78 mmol, 93%) as a colorless oil. MS (ESI^+^): *m*/*z* [MH^+^] 234. ^1^H NMR (CDCl_3_) δ: 3.71–3.66 (m, 2H), 3.64–3.57 (m, 4H), 3.55–3.49 (m, 2H), 2.87 (t, *J* = 4.9 Hz, 2H), 2.47 (dd, *J* = 12.2, 5.9 Hz, 2H), 2.37 (s, 2H), 1.41 (s, 9H). ^13^C NMR (151 MHz, CDCl_3_) δ 170.9, 80.7, 73.6, 70.5, 70.3, 66.9, 41.6, 36.3, 28.2; in agreement with the reported data [32].

*Tert-butyl 3-(2-(2-(2-aminoethoxy)ethoxy)ethoxy)propanoate* (**28**).

Compound **24** (0.93 g, 2.28 mmol) was treated with NH_2_NH_2_·2H_2_O (1.14 g, 0.022 mol) in EtOH (15 mL) using the general procedure described above to obtain **28** (0.578 g, 2.07 mmol, 91%) as a colorless oil. ^1^H NMR (CDCl_3_) δ: 3.71–3.66 (m, 2H), 3.64–3.57 (m, 8H), 3.55–3.49 (m, 2H), 2.87 (t, *J =* 4.9 Hz, 2H), 2.47 (dd, *J =* 12.2, 5.9 Hz, 2H), 2.37 (s, 2H), 1.41 (d, *J =* 4.8 Hz, 9H). ^13^C NMR (151 MHz, CDCl_3_) δ 170.8, 80.7, 72.8, 70.5 (multiple overlapping peaks), 70.3, 67.0, 41.6, 36.3, 28.2. MS (ESI^+^): *m*/*z* 279 [MH^+^].

*Tert-butyl 1-amino-3,6,9,12-tetraoxapentadecan-15-oate* (**29**).

Compound **25** (0.959 g, 2.12 mmol) was treated with NH_2_NH_2_·2H_2_O (0.53 g, 0.01 mol) in EtOH (15 mL) using the general procedure described above to obtain **29** (0.49 g, 1.52 mmol, 71%) as a colorless oil. MS (ESI^+^): *m*/*z* 322 [MH^+^]. ^1^H NMR (400 MHz, CDCl3) *δ* 3.70 (t, *J* = 6.5 Hz, 2H), 3.66−3.57 (m, 8H), 3.50 (t, *J* = 5.2 Hz, 2H), 2.86 (t, *J* = 5.2 Hz, 2H), 2.49 (t, *J* = 6.5 Hz, 2H), 1.43 (s, 9H); in agreement with that reported in the literature [32].

*Tert-butyl 3-(2-(2-(4-(aminomethyl)-1h-1,2,3-triazol-1-yl)ethoxy)ethoxy)propanoate* (**41**).

Compound **40** (0.235 g, 0.528 mmol) was treated with NH_2_NH_2_·2H_2_O (0.264 g, 5.28 mol) in EtOH (4 mL) using the general procedure described above to obtain **41** (0.125 g, 0.398 mmol, 75%) as a colorless oil. MS (ESI^+^): *m*/*z* 315 [MH^+^]. ^1^H NMR (400 MHz, CDCl3) *δ* 7.66 (s, 1H), 4.44 (m, 2H), 3.96 (s, 2H), 3.78 (m, 2H), 3.62 (m, 2H), 3.52 (m, 4H), 3.35 (m, 2H), 2.42 (t, *J* = 6.4 Hz, 2H), 1.37 (s, 9H). ^13^C NMR (151 MHz, CDCl_3_) δ 170.9, 148.0, 122.5, 80.7, 70.5, 70.3, 69.5, 66.9, 50.2, 37.2, 36.3, 28.1.

**General procedure for the synthesis of compounds 32–35.**

Amines **26**–**29** and **41**(1 eq) were dissolved in DMF, DIPEA (2 eq) and **31** (1 eq) were added and the reaction mixture was stirred at 90 °C for 16 h. When the reaction was complete (TLC), the solvent was removed and the obtained residue treated with water (5 mL), extracted with DCM (3 × 20 mL) and the combined organic phases washed with brine (20 mL). After evaporation of the solvent, the protected intermediate was purified by flash chromatography (PE/EtOAc 1/1) to afford the tert-butyl protected acids. The purified protected intermediates were dissolved in a solution of TFA 10% in DCM and stirred overnight at rt. The solvent was evaporated and the precipitate washed several times with DCM. The product was purified by flash chromatography (DCM/MeOH 98/2 to 95/5) to afford the desired acids **32**–**35**.

3-(2-((2-(2,6-dioxopiperidin-3-yl)-1,3-dioxoisoindolin-4-yl)amino)ethoxy)propanoic acid (**32**).

Compound **31** (0.357 g, 1.44 mmol) was reacted with **26** (0.273 g, 1.44 mmol) and DIPEA (0.558 g; 4.32 mmol) in DMF (3 mL) using the general procedure reported above to give, after purification by flash chromatography (PE/EtOAc 1/1), the desired protected acid as a yellow oil. This intermediate was hydrolyzed with a 10% TFA in DCM solution (10 mL). The crude product was purified by flash chromatography (DCM/MeOH 98/2) to give **32** (0.141 g, 0.363 mmol, 25% yield) as a yellow solid. ^1^H NMR (CDCl_3_) δ: 9.03 (s, 1H), 7.44 (dd, *J* = 8.5, 7.1 Hz, 1H), 7.05 (d, *J* = 7.1 Hz, 1H), 6.87 (d, *J* = 8.6 Hz, 1H), 6.44 (s, 1H), 5.00–4.85 (m, 1H), 3.73 (t, *J* = 6.0 Hz, 2H), 3.67 (t, *J* = 5.3 Hz, 2H), 3.42 (s, 2H), 2.88–2.66 (m, 3H), 2.58 (t, *J* = 6.0 Hz, 2H), 2.12–2.01 (m, 1H). ^13^C NMR (CDCl_3_) δ: 175.8, 172.2, 169.4, 167.79 (s), 146.92 (s), 136.09 (s), 132.51 (s), 117.05 (s), 111.77 (s), 110.35 (s), 69.30 (s), 66.31 (s), 48.90 (s), 42.26 (s), 34.76 (s), 31.40 (s), 22.78 (s). MS (ESI^−^): *m*/*z* 388 [M-H].

3-(2-(2-((2-(2,6-dioxopiperidin-3-yl)-1,3-dioxoisoindolin-4-yl)amino)ethoxy)ethoxy)propanoic acid (**33**).

Compound **31** (0.389 g, 1.41 mmol) was reacted with **27** (0.329 g, 1.41 mmol) and DIPEA (0.364 g, 2,82 mmol) in DMF (3 mL) using the general procedure reported above to give, after purification by flash chromatography (PE/EtOAc 1/1), the desired protected acid as a yellow oil. This intermediate was hydrolyzed with 10% TFA in DCM solution (10 mL). The crude product was purified by flash chromatography (DCM/MeOH 98/2) to give **33** (0.06 g, 0.138 mmol, 10% yield) as a yellow solid. ^1^H NMR (CD_3_CN) δ: 9.06 (s, 1H), 7.56–7.44 (m, 1H), 7.05–6.93 (m, 2H), 6.45 (s, 1H), 4.98–4.84 (m, 1H), 3.65–3.59 (m, 5H), 3.54 (td, *J* = 5.6, 2.8 Hz, 5H), 3.42 (t, *J* = 4.5 Hz, 3H), 2.80–2.57 (m, 4H), 2.51–2.41 (m, 2H), 2.06 (dd, *J* = 8.6, 3.7 Hz, 1H), 1.91 (dd, *J* = 4.8, 2.4 Hz, 1H). ^13^C NMR (CD_3_CN) δ: 172.6, 172.3, 169.8, 169.5, 167.7, 146.9, 136.2, 132.6, 117.3, 110.9, 109.9, 70.1, 70.0, 69.1, 66.4, 48.9, 42.0, 34.4, 31.1, 22.4. MS (ESI^−^): *m*/*z* 432 [M-H]^−^.

3-(2-(2-(2-((2-(2,6-dioxopiperidin-3-yl)-1,3-dioxoisoindolin-4-yl)amino)ethoxy)ethoxy)ethoxy)propanoic acid (**34**).

Compound **31** (0.337 g, 1.22 mmol) was reacted with **28** (0.339 g, 1.22 mmol) and DIPEA (0.315 g, 2.44 mmol) in DMF (3 mL) using the general procedure reported above to give, after purification by flash chromatography (PE/EtOAc 1/1), the desired protected acid as a yellow oil. This intermediate was hydrolyzed with 10% TFA in DCM solution (10 mL). The crude product was purified by flash chromatography (DCM/MeOH 98/2) to give **34** (0.101 g, 0.211 mmol, 17% yield) as a yellow solid. ^1^H NMR (CDCl_3_) δ: 8.94 (s, 1H), 7.46 (dd, *J* = 8.5, 7.1 Hz, 1H), 7.07 (d, *J* = 7.1 Hz, 1H), 6.89 (d, *J* = 8.5 Hz, 1H), 4.92 (dd, *J* = 12.3, 5.4 Hz, 1H), 3.78–3.58 (m, 13H), 3.49–3.40 (m, 2H), 2.76–2.60 (m, 3H), 2.59 (t, *J* = 6.2 Hz, 2H), 2.10 (m, 1H). ^13^C NMR (CDCl_3_) δ: 175.5, 172, 169.4, 169.1, 167.7, 146.9, 136.2, 132.6, 116.9, 111.7, 110.3, 70.8, 70.7, 70.5, 69.5, 66.4, 48.9, 42.4, 34.8, 31.4, 22.9. MS (ESI^−^): *m*/*z* 476 [M−H]^−^.

1-((2-(2,6-dioxopiperidin-3-yl)-1,3-dioxoisoindolin-4-yl)amino)-3,6,9,12-tetraoxapentadecan-15-oic acid (**35**).

Compound **31** (0.429 g, 1.55 mmol) was reacted with **29** (0.500 g, 1.55 mmol) and DIPEA (0.400 g, 3.1 mmol) in DMF (3 mL) using the general procedure reported above to afford, after purification by flash chromatography (PE/EtOAc 1/1), the desired protected acid as a yellow oil. This intermediate was hydrolyzed with 10% TFA in DCM solution (10 mL). The crude product was purified by flash chromatography (DCM/MeOH 98/2) to give **35** (0.178 g, 0.341 mmol, 21% yield) as a yellow oil. ^1^H NMR (DMSO-D6) δ: 11.07 (s, 1H), 7.54 (dd, *J* = 8.6, 7.1 Hz, 1H), 7.11 (d, *J* = 8.6 Hz, 1H), 7.00 (d, *J* = 7.1 Hz, 1H), 6.56 (m, 1H), 5.02 (dd, *J* = 12.9, 5.4 Hz, 1H), 3.77–3.17 (m, 18H), 2.84 (m, 1H), 2.60–2.44 (m, 2H), 2.37 (m, 2H), 2.03–1.93 (m, 1H). ^13^C NMR (DMSO-D6) δ: 173.3, 170.9, 170.6, 169.4, 167.8, 146.9, 136.8, 132.6, 118, 111.2, 109.7, 72.9, 70.3, 69.4, 66.7, 60.7, 49.1, 42.2, 36.3, 31.5, 22.7. MS (ESI^−^): *m*/*z* 520 [M−H]^−^.

*Tert-butyl 3-(2-(2-bromoethoxy)ethoxy)propanoate* (**37**).

Compound **19** (1.10 g, 4.69 mmol, 1 eq) was dissolved in THF under nitrogen atmosphere. CBr_4_ (3.08 g, 9.39 mmol, 2 eq) and PPh_3_ (2.46 g, 9.39 mmol, 2 eq) were successively added and the reaction mixture was stirred at rt for 4 h. The formed white precipitate was filtered off, the solvent removed under reduced pressure and the residue purified by flash chromatography (PE/EtoAc 8/2) to afford **37** (1.38 g, 4.64 mmol, 100%) as a white oil. The compound was characterized by MS and used in the next step without further characterization. MS (ESI^+^): *m*/*z* 297/299 [MH^+^].

*Tert-butyl 3-(2-(2-azidoethoxy)ethoxy)propanoate* (**38**).

Compound **37** (0.42 g, 1.41 mmol, 1 eq) was dissolved in DMF (2 mL). NaN_3_ (0.46 g, 7.06 mmol, 5 eq) was added, followed by few drops of water in order to allow complete dissolution of the reagents. The reaction mixture was stirred at 70 °C for 16 h. When the reaction was complete (TLC), the mixture was saturated with NaCl and extracted with DCM (3 × 20 mL). The solvent was removed under reduced pressure to give **38** (0.38 g, 1.46 mmol, 85% yield) as a white oil. The obtained product was used in the following steps without further purification. ^1^H NMR (CDCl_3_) δ: 3.70 (t, *J* = 6.5 Hz, 2H), 3.67–3.58 (m, 5H), 3.41–3.34 (m, 2H), 2.49 (t, *J* = 6.5 Hz, 2H), 1.43 (s, 9H). MS (ESI): *m*/*z* 282 [MNa+].

*Tert-butyl 3-(2-(2-(4-((1,3-dioxoisoindolin-2-yl)methyl)-1H-1,2,3-triazol-1-yl)ethoxy)ethoxy)propanoate* (**40**).

N-propargyl phthalimide (**39**, 0.109 g, 0.0593 mmol) and the previously synthesized azide **38** (0.154 g, 0.0593 mmol) were dissolved in *t*-BuOH/H_2_O 1/1 (10 mL) at rt. To the obtained mixture, CuSO_4_ (0.009 g, 0.0059 mmol) and sodium ascorbate (0.023 g; 0.0118 mmol) were added, and the reaction was stirred at 90 °C for 3 h. After cooling to rt, the mixture was treated with an aqueous solution of EDTA (5 mL). The aqueous phase was extracted with DCM (3 × 20 mL), the organic phase was washed with brine (5 mL), dried, and the solvent removed under reduced pressure. The crude product was purified by flash chromatography (DCM/MeOH 98/2) to afford **40** (0.261 g; 0. 587 mmol; 90% yield) as a white solid. ^1^H NMR (CDCl_3_) δ: 7.82 (dd, *J* = 5.4, 3.0 Hz, 2H), 7.74 (s, 1H), 7.68 (dd, *J* = 5.4, 3.0 Hz, 2H), 4.96 (s, 2H), 4.45 (t, *J* = 5.1 Hz, 2H), 3.83–3.74 (m, 2H), 3.65 (t, *J* = 6.4 Hz, 2H), 3.53 (s, 4H), 2.45 (d, *J* = 6.4 Hz, 2H), 1.40 (s, 9H). ^13^C NMR (CDCl_3_) δ: 170.9, 167.7, 142.7, 134.1, 132.2, 124.0, 123.5, 80.6, 70.6, 70.3, 69.5, 67.0, 50.3, 36.3, 33.2, 28.2. MS (ESI^+^): *m*/*z* 244 [MH^+^].

**General procedure for the synthesis of compounds 42 and 46.**

The appropriate amine **41** or **44** (1 eq) was dissolved in DMF (3 mL) then DIPEA (2 eq) and **31** (1 eq) were added. The reaction mixture was stirred for 16 h at 90 °C. When the reaction was complete (TLC), the solvent was removed and the residue dissolved in water and extracted with DCM (3 × 15 mL). The organic phase was washed with brine, dried and evaporated under reduced pressure. The product was isolated by flash chromatography (PE/EtOac 1/1).

*Tert-butyl 3-(2-(2-(4-(((2-(2,6-dioxopiperidin-3-yl)-1,3-dioxoisoindolin-4-yl)amino)methyl)-1H-1,2,3-triazol-1-yl)ethoxy)ethoxy)propanoate* (**42**).

Compound **31** (0.109 g, 0.397 mmol) was reacted with amine **41** (0.125 g, 0.397 mmol) and DIPEA (0,102 g, 0.795 mmol) using the general procedure described above to give **42** (0.075 g, 0.131 mmol, 33% yield) as a yellow oil. ^1^H NMR (CDCl_3_) δ: 8.91 (s, 1H), 8.45 (s, 1H), 7.69 (s, 1H), 7.48–7.44 (m, 1H), 7.12–7.07 (m, 1H), 7.02 (d, *J* = 8.5 Hz, 1H), 6.70 (t, *J* = 5.9 Hz, 1H), 4.95–4.83 (m, 1H), 4.61 (d, *J* = 5.9 Hz, 2H), 4.51–4.46 (m, 2H), 3.83–3.79 (m, 2H), 3.64 (m, 2H), 3.58–3.49 (m, 4H), 2.87–2.67 (m, 3H), 2.50–2.42 (m, 2H), 2.15–2.06 (m, 1H), 1.41 (s, 9H). ^13^C NMR (CDCl_3_) δ: 171.2, 171.0, 169.4, 168.5, 167.6, 146.3, 144.9, 136.2, 132.5, 123.1, 117.3, 112.2, 110.8, 80.7, 70.7, 70.5, 70.4, 70.3, 69.4, 67.0, 50.4, 49.0, 38.7, 36.3, 31.5, 28.2, 22.8, 21.0. MS (ESI^−^): *m*/*z* 569 [M−H]^−^.

*Tert-butyl 3-(2-(2-(4-(((2-(2,6-dioxopiperidin-3-yl)-1,3-dioxoisoindolin-4-yl)amino)methyl)-1H-1,2,3-triazol-1 yl)ethoxy)ethoxy)propanoate* (**46**).

Compound **31** (0.136 g, 0.492 mmol) was reacted with amine **44** (0.07 g, 0.492 mmol) and DIPEA (0.187 g, 1.44 mmol) using the general procedure described above to give **46** (0.04 g, 0.100 mmol, 20% yield) as a yellow oil. ^1^H NMR (CDCl_3_) δ 8.59 (s, 1H), 7.45 (m, 1H), 7.06 (d, *J* = 6.9 Hz, 1H), 6.89 (d, *J* = 8.5 Hz, 1H), 6.45 (m, 1H), 4.94–4.85 (m, 1H), 4.17 (s, 2H), 3.73–3.62 (m, 6H), 3.45 (m, 2H), 2.85–2.73 (m, 3H), 2.42 (s, 2H), 2.11–2.03 (m, 1H). ^13^C NMR (CDCl_3_) δ 171.6, 169.4, 168.7, 167.7, 146.9, 136.1, 132.6, 116.9, 111.7, 110.3, 79.7, 74.7, 70.5, 69.7, 69.2, 58.5, 48.9, 42.4, 31.5, 22.8. MS (ESI^−^): *m*/*z* 398 [M−H]^−^.

3-(2-(2-(4-(((2-(2,6-dioxopiperidin-3-yl)-1,3-dioxoisoindolin-4-yl)amino)methyl)-1H-1,2,3-triazol-1-yl)ethoxy)ethoxy)propanoic acid (**43**).

Compound **42** (0.075 g, 0.122 mmol) was treated with a solution of TFA 10% in DCM (10 mL) and the reaction mixture was stirred at rt for 16 h. The solvent was evaporated and the obtained residue purified by flash chromatography (DCM/MeOH 95/5) to give **43** (0.05 g; 0.097 mmol; 74%) as a yellow solid. ^1^H NMR (CDCl_3_) δ: 9.16–9.13 (m, 1H), 7.78 (s, 1H), 7.43 (t, *J* = 7.9 Hz, 1H), 7.06–7.03 (m, 2H), 6.80 (s, br, 1H), 4.93–4.90 (m, 1H), 4.61 (s, 2H), 4.47 (t, *J* = 4.7 Hz, 2H), 3.79 (t, *J* = 4.8 Hz, 2H), 3.67 (t, *J* = 6.0 Hz, 2H), 3.55–3.49 (m, 4H), 2.86–2.65 (m, 3H), 2.55 (t, *J* = 6.0 Hz, 2H), 2.11–2.05 (m, 1H). ^13^C NMR (CDCl_3_) δ: 174.7, 172.0, 169.6, 169.2, 167.6, 146.3, 145.2, 136.3, 132.4, 123.7, 117.6, 112.2, 110.4, 70.3, 70.2, 69.2, 66.5, 50.4, 49.0, 38.4, 34.8, 31.5, 22.8. MS (ESI^−^): *m*/*z* 513 [M−H]^−^.

**General procedure for the synthesis of compounds 47 and 48.**

The appropriate alkyne **45** or **46** (1 eq) and the azide **38** (1 eq) were dissolved in t-BuOH/H_2_O 1/1. To the obtained solution, CuSO_4_ (0.1 eq) and sodium ascorbate (0.2 eq) were successively added. The mixture was stirred at 90 °C for 3 h. After cooling to rt, the mixture was treated with an aqueous solution of EDTA (5 mL). The aqueous phase was extracted with DCM (3 × 20 mL). The organic phase was washed with brine (5 mL), dried, and the solvent removed under reduced pressure. The crude product was purified by flash chromatography (DCM/MeOH 98/2) to afford the desired protected acid as a colorless oil. This intermediate was dissolved in a solution of TFA 10% in DCM and stirred for 12 h. The solvent was evaporated under reduced pressure and the crude product was purified by flash chromatography (DCM/MeOH 95:5).

3-(2-(2-(4-((3-((2-(2,6-dioxopiperidin-3-yl)-1,3-dioxoisoindolin-4-yl)amino)-3-oxopropoxy)methyl)-1H-1,2,3-triazol-1-yl)ethoxy)ethoxy)propanoic acid (**47**).

Compound **38** (0.03 g, 0.08 mmol) was reacted with **45** (0.03 g, 0.08 mmol), CuSO_4_ 10% mmol and sodium ascorbate 20% mmol in t-ButOH/H_2_O 1/1 (4 mL) using the general procedure described above to obtain, after flash chromatography, a colorless oil. The protected acid was hydrolyzed with a solution of TFA 10% in DCM (10 mL) as described above. The crude product was purified by flash chromatography (DCM/MeOH 95:5) to give **47** (0.05 g; 0.085 mmol, 99%) as a white solid. ^1^H NMR (CDCl_3_) δ: 9.95 (s, 1H), 9.47 (s, 1H), 8.79 (d, *J* = 8.4 Hz, 1H), 7.77 (s, 1H), 7.66 (dd, *J* = 8.4, 7.5 Hz, 1H), 7.52–7.47 (m, 1H), 4.98–4.96 (m, 1H), 4.80 -4.73 (m, 2H), 4.55–4.43 (m, 2H), 3.89–3.82 (m, 4H), 3.74 -3.72 (m, 2H), 3.69–3.52 (m, 2H), 3.37–3.33 (m, 2H), 2.78–2.70 (m, 3H), 2.61 (t, *J* = 6.3 Hz, 2H), 2.53 (t, *J* = 6.0 Hz, 2H), 2.16–2.09 (m, 1H). ^13^C-NMR (CDCl_3_) δ: 174.7, 173.8, 173.7, 169.4, 168.8, 167.7, 145.9, 144.7, 136.0, 132.6, 124.2, 116.8, 111.4, 110.3, 70.4, 70.3, 69.7, 69.5, 67.0, 64.9, 50.3, 48.9, 38.4, 34.8, 31.5, 22.8. MS (ESI^−^): *m*/*z* 585 [M−H]^−^.

3-(2-(2-(4-((2-(2-((2-(2,6-dioxopiperidin-3-yl)-1,3-dioxoisoindolin-4-yl)amino)ethoxy)ethoxy)methyl)-1H-1,2,3-triazol-1-yl)ethoxy)ethoxy)propanoic acid (**48**).

Compound **46** (0.064 g, 0.17 mmol) was reacted with **38** (0.035 g, 0.16 mmol), CuSO_4_ 10% mmol and sodium ascorbate 20% mmol in t-ButOH/H_2_O 1/1 (5 mL) using the general procedure describe above to obtain, after flash chromatography, a yellow oil. The protected acid was hydrolyzed with a solution of TFA 10% in DCM (10 mL) as described above. follow the general procedure. The crude product was purified by flash chromatography (DCM/MeOH 95:5) to give **48** (0.134 g; 0.222 mmol, 74% yield) as a bright yellow solid. ^1^H NMR (CDCl_3_) δ: 8.84 (s, 1H), 7.70 (s, 1H), 7.48–7.44 (m, 1H), 7.04 (d, *J* =7.0 Hz, 1 H), 6.87 (d, *J* =8.6 Hz, 1H), 6.45 (t, *J* = 5.6 Hz, 1H), 4.90–4.87 (m, 1H), 4.65 (s, 2H), 4.48–4.46 (m, 2H), 3.83–3.64 (m, 2H), 3.67–3.63 (m, 8H), 3.53–3.52 (m, 4H), 3.42–3.41 (m, 2H), 2.87–2.67 (m, 3H), 2.44 (t, *J* = 6.5 Hz, 2H), 2.06 (m, 1H). ^13^C NMR (CDCl_3_) δ: 174.6, 170.9, 169.4, 168.8, 167.7, 146.8, 144.8, 136.1, 132.6, 124.0, 116.8, 111.6, 110.3, 70.5, 70.4 (overlapping peaks), 70.2, 69.7, 69.5, 66.9, 64.6, 50.2, 48.9, 42.4, 36.2, 28.2, 22.8. MS (ESI^−^): *m*/*z* 601 [M−H]^−^.

**General procedure for the synthesis of final compounds 5–11.**

To a solution of the appropriate acids **30**, **32–36** and **43** in dry DMF, HATU (1.2 eq) or HOBt (0.15 eq)/HBTU (1.5 eq) and DIPEA (2 eq) were added and the reaction mixture was stirred at rt. After 2 h, **16** (1 eq) was added and the reaction was stirred at rt for further 16 h. The solvent was evaporated under reduced pressure and the residue treated with an aqueous solution of NaHCO_3_ 10%. The water phase was extracted with DCM (3 × 20 mL) and the organic phase was washed with brine (5 mL). The organic phase was dried and the solvent evaporated under reduced pressure to leave a crude product which was purified by flash chromatography (DCM/MeOH 95/5).

N-((S)-1-((4-(3-(5-chloro-2-fluoro-3-(methylsulfonamido)phenyl)-1-isopropyl-1H-pyrazol-4-yl)pyrimidin-2-yl)amino)propan-2-yl)-4-((2-(2,6-dioxopiperidin-3-yl)-1,3-dioxoisoindolin-4-yl)amino)butanamide (**5**).

Compound **30** (0.053 g, 0.14 mmol) was reacted with HATU (0.067 g, 0.17 mmol), DIPEA (0.038 g, 0.29 mmol) and **16** (0.085 g, 0.17 mmol) in dry DMF (3 mL) using the general procedure reported above to give **5** (0.075 g, 0.091 mmol, 61%) as a bright yellow solid. ^1^H NMR (CDCl_3_) δ: 9.57 (s, 1H), 8.81 (d, *J* = 16.8 Hz, 1H), 8.20 (s, br, 1H), 8.16 (s, 1H), 7.87 (s, 1H), 7.61 (d, *J* = 6.1 Hz, 1H), 7.45–7.35 (m, 2H), 7.00 (d, *J* = 6.7 Hz, 1H), 6.89–6.82 (m, 1H), 6.69 (m, 2H), 4.91 (s, 1H), 4.62–4.55 (m, 1H), 3.95 (m, 1H), 3.41–3.17 (m, 4H), 3.04 (s, 3H), 2.86–2.63 (m, 3H), 2.28 (m, 2H), 2.08 (m, 1H), 1.88 (m, 2H), 1.60 (d, *J* = 6.6 Hz, 6H), 0.95 (s, 3H). ^13^C NMR (CDCl_3_) δ: 174.3, 171.9, 169.5, 169.2, 167.8, 167.0, 162.1, 154.8, 150.6 (d, *J* = 249 Hz), 146.8, 145.4, 144.3, 136.3, 132.4, 131.2, 130.0, 127.1, 126.4, 124.3, 123.6, 118.2, 116.9, 111.6, 109.9, 105.8, 55.5, 48.9, 45.6, 44.3, 41.9, 40.4, 33.4, 31.4, 24.9, 22.8, 22.7, 17.5. MS (ESI^−^): *m*/*z* 821/823 [M−H]^−^.

N-((S)-1-((4-(3-(5-chloro-2-fluoro-3-(methylsulfonamido)phenyl)-1-isopropyl-1H-pyrazol-4-yl)pyrimidin-2-yl)amino)propan-2-yl)-3-(2-((2-(2,6-dioxopiperidin-3-yl)-1,3-dioxoisoindolin-4-yl)amino)ethoxy)propanamide (**6**).

Compound **32** (0.14 g; 0.36 mmol) was reacted with HATU (0.164 g; 0.43 mmol), DIPEA (0.139 g; 1.08 mol) and **16** (0.173 g; 0.36 mmol) in dry DMF (3 mL) using the general procedure described above to give **6** (0.128 g; 0.15 mmol; 42% yield) as a bright yellow solid. ^1^H NMR (CDCl_3_) δ: 8.12 (d, *J* = 5.3 Hz, 1H), 8.05 (d, *J* = 4.6 Hz, 1H), 7.94 (s, 1H), 7.54–7.50 (m, 1H), 7.47–7.41 (m, 1H), 7.38–7.36 (m, 1H), 7.05 (m, 2H), 6.85 (m 2H), 6.49 –6.44 (m, 2H), 6.41 (s, 1H), 4.93–4.83 (m, 1H), 4.54 (m, *1*H), 3.61 (m, *5*H), 3.35 (m, 2H), 3.12 (m, 1H), 2.94 (s, 3H), 2.82–2.59 (m, 4H), 2.38–2.24 (m, 2H), 2.12–1.99 (m, 1H), 1.65–1.47 (m, 6H), 0.91 (s, 3H). ^13^C NMR (DMSO-d*_6_*) δ: 173.4, 170.5, 170.1, 169.3, 167.9, 162.4, 158.8, 158.6, 151.7 (d, *J* = 250 Hz), 146.9, 142.1, 136.7, 132.6, 130.5, 130.4, 128.1, 127.4, 125.9, 124.5, 119.8, 117.9, 111.2, 109.8, 106.1, 69.5, 67.4, 54.3, 49.1, 46.0, 44.7, 42.1, 40.8, 36.8, 31.5, 22.9, 22.6, 18.3. MS (ESI^−^): *m*/*z* 852/854 [M−H]^−^.

N-((S)-1-((4-(3-(5-chloro-2-fluoro-3-(methylsulfonamido)phenyl)-1-isopropyl-1H-pyrazol-4-yl)pyrimidin-2-yl)amino)propan-2-yl)-3-(2-(2-((2-(2,6-dioxopiperidin-3-yl)-1,3-dioxoisoindolin-4-yl)amino)ethoxy)ethoxy)propanamide (**7**).

Compound **33** (0.066 g; 0.152 mmol) was reacted with HOBt (0.003 g; 0,002 mmol), HBTU (0.086 g, 0.228 mmol), DIPEA (0.039 g, 0.304 mmol) and **16** (0.065 g; 0.136 mmol) in dry DMF (3 mL) using the general procedure described above to give **7** (0.054 g, 0.60 mml, 45%) as a bright yellow solid. ^1^H NMR (DMSO-d*_6_*) δ: 11.08 (s, 1H), 9.86 (s, 1H), 8.57 (s, br, 1H), 8.07 (s, 1H), 7.54–7.49 (m, 2H), 7.45 (s, 1H), 7.29 (s, 1H), 7.07 (d, *J* = 8.6 Hz, 1H), 6.99 (m, 1H), 6.69 (s, br, 1H), 6.55 (m, 2H), 5.02 (dd, *J* = 12.8, 5.4 Hz, 1H), 4.54 (dt, *J* = 13.2, 6.6 Hz, 1H), 3.78 (m, 1H), 3.55–3.39 (m, 12H), 3.01 (s, 3H), 2.84–2.52 (m, 3H), 2.21 (m,2H), 2.03–1.93 (m, 1H), 1.45 (d, *J* = 6.6 Hz, 6H), 0.83 (s, 3H). ^13^C NMR (DMSO-d*_6_*) δ: 173.4, 170.6, 170.2, 169.4, 167.8, 162.6, 158.8, 158.7, 151.9 (d, *J* = 249 Hz), 146.9, 142.2, 136.7, 132.6, 130.5, 130.4, 128.2, 127.3, 125.8, 124.6, 119.9, 117.9, 111.2, 109.8, 106.1, 70.2, 70.1, 69.4, 67.4, 54.4, 49.1, 46.1, 44.7, 42.2, 40.8, 36.8, 31.5, 23.0, 22.6, 18.4. MS (ESI^−^): *m*/*z* 895/897 [M−H]^−^.

N-((S)-1-((4-(3-(5-chloro-2-fluoro-3-(methylsulfonamido)phenyl)-1-isopropyl-1H-pyrazol-4-yl)pyrimidin-2-yl)amino)propan-2-yl)-3-(2-(2-(2-((2-(2,6-dioxopiperidin-3-yl)-1,3-dioxoisoindolin-4-yl)amino)ethoxy)ethoxy)ethoxy)propanamide (**8**).

Compound **34** (0.043 g, 0.09 mmol) was reacted with HOBt (0.002 g, 0,00135 mmol), HBTU (0.051 g, 0.13 mmol), DIPEA (0.013 g, 0.10 mmol) and **16** (0.043 g, 0.09 mmol) in dry DMF (3 mL) using the general procedure described above to give **8** (0.04 g, 0.042 mmol, 47%) as a bright yellow solid. ^1^H NMR (DMSO-d*_6_*) δ: 11.07 (s, 1H), 9.89 (s, 1H), 8.83 (s, br 1H), 8.20 (s, br, 1H), 7.60 (s, br 1H), 7.55–7.51 (m, 2H), 7.48 (d, *J* = 6.1 Hz, 1H), 7.32 (d, *J* = 4.5 Hz, 1H), 7.09 (d, *J* = 8.6 Hz, 1H), 6.99 (d, *J* = 7.0 Hz, 1H), 6.91 (s, br, 1H), 6.55 (s, 1H), 5.01 (dd, *J* = 12.9, 5.4 Hz, 1H), 4.64–4.50 (m, 1H), 3.76 (s, 1H), 3.57–3.38 (m, 16H), 3.04 (s, 3H), 2.84–2.52 (m, 3H), 2.22 (m,, 2H), 2.01–1.95 (m, 1H), 1.47 (d, *J* = 6.5 Hz, 7H), 0.81 (s, 3H). ^13^C NMR (CD_3_CN) δ: 172.2, 171.8, 169.8, 169.5, 167.6, 166.6, 154.6, 151.2 (d, *J* = 255 Hz), 146.8, 145.7, 143.4, 136.2, 132.7, 132.6, 128.9, 127.0, 126.9, 124.4, 124.3, 117.2, 115.4, 111.0, 109.9, 105.7, 70.1–69.0 (three signals, multiple overlapping peaks), 66.8, 55.1, 48.9, 45.8, 43.8, 42.0, 40.1, 36.4, 31.50, 22.4, 21.8, 17.0. MS (ESI^−^): *m*/*z* 939/941 [M−H]^−^.

N-((S)-1-((4-(3-(5-chloro-2-fluoro-3-(methylsulfonamido)phenyl)-1-isopropyl-1H-pyrazol-4-yl)pyrimidin-2-yl)amino)propan-2-yl)-1-((2-(2,6-dioxopiperidin-3-yl)-1,3-dioxoisoindolin-4-yl)amino)-3,6,9,12-tetraoxapentadecan-15-amide (**9**).

Compound **35** (0.071 g, 0.13 mmol) was reacted with HOBt (0.001 g, 0,00186 mmol), HBTU (0.07 g, 0.186 mmol), DIPEA (0.032 g, 0.24 mmol) and **16** (0.06 g, 0.12 mmol) in dry DMF (3 mL) using the general procedure described above to give **9** (0.05 g, 0.05 mmol, 41%) as a bright yellow solid. ^1^H NMR (CD_3_CN) δ: 9.15 (s, br, 1H), 9.05 (s, br, 1H), 8.47 (s, 1H), 8.16 (s, 1H), 7.98–7.87 (m, 1H), 7.58 (m, 1H), 7.51 (m, 1H), 7.36 (m, 1H), 7.01 (m, 3H), 6.87–6.85 (m, 1H), 6.64–6.83 (m, 1H), 4.92 (m, 1H), 4.61 (m, 1H), 3.88 (m, 1H), 3.66–3.42 (m, 21H), 3.12–3.09 (m, 2 H), 3.03 (s, 3H), 2.89–2.63 (m, 3H), 2.30 (m, 1H), 2.03–1.93 (m, 1H), 1.54 (d, *J* = 6.5 Hz, 6H), 0.93 (s, 3H). ^13^C NMR (CD_3_CN) δ: 173.3, 172.6, 170.8, 170.5, 168.7, 168.6, 167.3, 161.4, 156.0, 152.2 (d, *J* = 247 Hz), 147.8, 147.3, 144.8, 138.8, 137.2, 133.5, 129.9, 128.0, 125.3, 122.5, 119.0, 111.9, 110.9, 106.7, 71.1–70.9 (four signals, multiple overlapping peaks), 70.7, 70.1, 67.8, 56.1, 50.0, 45.9, 44.7, 42.4, 40.9, 36.8, 31.5, 22.9, 22.5, 18.3. MS (ESI^−^): *m*/*z* 983/985 [M−H]^−^.

N-((S)-1-((4-(3-(5-chloro-2-fluoro-3-(methylsulfonamido)phenyl)-1-isopropyl-1H-pyrazol-4-yl)pyrimidin-2-yl)amino)propan-2-yl)-1-((2-(2,6-dioxopiperidin-3-yl)-1,3-dioxoisoindolin-4-yl)amino)-3,6,9,12,15-pentaoxaoctadecan-18-amide (**10**).

Compound **36** (0.03 g, 0.05 mmol) was reacted with HATU (0.024 g, 0.06 mmol), DIPEA (0.14 g, 0.10 mmol) and **16** (0.035 g, 0.07 mmol) in dry DMF (3 mL) using the general procedure reported above to give **10** (0.027 g, 0.026 mmol, 40%) as a bright yellow solid. ^1^H NMR (CD_3_CN) δ: 8.19 (s, br, 1H), 8.10–8.09 (m, 1H), 7.52–7.49 (m, 2H), 7.37 (m, 1H), 7.01–7.00 (m, 2H), 6.64–6.63 (s, br, 1H), 6.45 (m, 1H), 4.90 (m, 1H), 4.55 (m, 1H), 3.64–3.63 (m, 1H), 3.53- 3.40 (m, 23H), 2.95 (s, 3H), 2.71–2.62 (m, 4H), 2.07–2.04 (m, 2H), 1.51 (d, *J* = 6.5 Hz, 6H), 0.86 (s, 3H). ^13^C NMR (DMSO-d*_6_*) δ: 174.0, 173.3, 171.4, 170.5, 168.7, 165.9, 161.4, 157.9, 154.8, 152.6 (d, *J* = 230 Hz), 147.9, 143.4, 137.2, 133.6, 130.8 (two overlapping peaks), 129.5, 128.3, 127.6, 125.4, 125.2, 122.2, 111.9, 111.0, 106.5, 71.2–71.1 (four overlapping signals), 71.0, 70.9, 70.1, 67.8, 55.6, 50.0, 43.0, 40.9, 37.6, 32.1, 23.4, 23.0 (two overlapping signals), 18.4. MS (ESI^+^): *m*/*z* 1030/1032 [MH^+^].

N-((S)-1-((4-(3-(5-chloro-2-fluoro-3-(methylsulfonamido)phenyl)-1-isopropyl-1H-pyrazol-4-yl)pyrimidin-2-yl)amino)propan-2-yl)-3-(2-(2-(4-(((2-(2,6-dioxopiperidin-3-yl)-1,3-dioxoisoindolin-4-yl)amino)methyl)-1H-1,2,3-triazol-1-yl)ethoxy)ethoxy)propanamide (**11**).

Compound **43** (0.05 g, 0.09 mmol) was reacted with HATU (0.04 g, 0.11 mmol), DIPEA (0.025 g, 0.19 mmol) and **16** (0.046 g, 0.09 mmol) in dry DMF (3 mL) using the general procedure described above to give **11** (0.04 g, 0.04 mmol, 42%) as a bright yellow solid. ^1^H NMR (CD_3_CN) δ: 8.19 (s, br 1H), 8.10 (m, 1H), 7.79–7.78 (m, 1H), 7.59–7.53 (m, 2H), 7.38 (m, 1H), 7.10 (dd, *J =* 8.6, 1.4 Hz, 1H), 7.04 (d, *J =* 7.2 Hz, 1H), 6.80 (m, 1H), 6.63 (s, br, 1H), 4.93- 4.90 (m, 1H), 4.59–4.55 (m, 3H), 4.43 (t, *J* = 5.0 Hz, 2H), 3.77 (m, 3H), 3.44–3.42 (m, 4H), 3.44–3.42 (m, 3H), 3.36 (m, 2h), 2,97 (m, 3H), 2.75–2.63 (m, 4H), 2.17–2.15 (m, 2H), 2.10–2.04 (m, 2H), 1.52–1.51 (d, *J* = 6.1 Hz, 6H), 1.30–1.32 (m, 1H), 0.90 (s, 3H). ^13^C NMR (CD_3_CN) δ: 173.4, 170.4, 168.7, 161.2, 158.6, 152.1 (d, *J* = 231 Hz), 147.3, 145.9, 143.3, 137.2, 133.6, 130.7, 128.4, 127.6, 127.5, 124.2, 120.5, 119.0, 112.3, 111,5, 107.5, 71.0, 70.9, 70.8, 69.9 (two overlapping peaks), 67.8, 55.6, 51.0, 50.0, 40.9, 38.9, 37.7, 32.1, 23.4, 23.0 (two overlapping peaks), 18.4. MS (ESI-): *m*/*z* 976/978 [M−H]^−^.

**General procedure for the synthesis of the final compounds 12 and 13.**

To a stirred solution of the appropriate acid **47** or **48** (1.0 eq) in dry DMF, HATU (1.2 eq) or HOBt (0.15 eq)/HBTU (1.5 eq) and DIPEA (2 eq) were added and the mixture was stirred at rt. After 2 h, **16** (1 eq) was added and the reaction was stirred at rt for further 16 h. The solvent was evaporated under reduced pressure and the residue treated with an aqueous solution of NaHCO_3_ 10%. The water phase was extracted with DCM (3 × 20 mL) and the organic phase was washed with brine (5 mL). The organic phase was dried and the solvent evaporated under reduced pressure to leave a crude product which was purified by flash chromatography (DCM/MeOH 95/5).

N-((S)-1-((4-(3-(5-chloro-2-fluoro-3-(methylsulfonamido)phenyl)-1-isopropyl-1H-pyrazol-4-yl)pyrimidin-2-yl)amino)propan-2-yl)-3-(2-(2-(4-((3-((2-(2,6-dioxopiperidin-3-yl)-1,3-dioxoisoindolin-4-yl)amino)-3-oxopropoxy)methyl)-1H-1,2,3-triazol-1-yl)ethoxy)ethoxy)propanamide (**12**).

Compound **12** was obtained by starting from **16** (0.035 g, 0.085 mmol), **47** (0.05 g, 0.085 mmol), HATU (0.038 g, 0.0102 mmol) and DIPEA (0.033 g, 0.25 mmol) in DMF (2 mL), using the general procedure described above to give a yellow solid (0.30 g, 0.028 mmol, 33%). ^1^H NMR (600 MHz, CDCl_3_) δ 10.2 (s, br, 1H), 10.04 (s, 1H), 8.83 (m, 1H), 8.11 (dd, *J* = 5.2, 1.4 Hz, 1H), 7.91 (d, *J* = 3.0 Hz, 1H), 7.74–7.69 (m, 2H), 7.57–7.53 (m, 2H), 7.43 (s, 1H), 6.49–6.42 (m, 2H), 4.96 (dd, *J* = 12.3, 6.0 Hz, 1H), 4.79–4.72 (m, 2H), 4.56 (dt, *J* = 13.4, 6.7 Hz, 1H), 4.54–4.46 (m, *2*H), 3.87–3.81 (m, 4H), 3.64–3.42 (m, 7H), 3.24 (m, 1H), 2.96 (s, 3H), 2.88–2.74 (m, 5H), 2.30–2.28 (m, 2H), 2.19–2.09 (m, 1H), 1.57 (d, *J* = 6.7 Hz, 6H), 0.97 (m, 3H). ^13^C NMR (151 MHz, CDCl_3_) δ 172.4, 171.0, 169.5, 168.7, 166.9, 162.0, 160.3, 157.3, 150.9 (d, *J* = 232 Hz), 144.8, 142.3. 137.6, 136.3, 131.4, 129.5, 128.6, 128.0, 126.0, 125.9, 124.7, 124.6, 120.0, 118.5, 115.8, 111,0, 109.9, 106.7, 70.2, 70.1, 69.4, 67.1, 65.7, 64.7, 55.5, 54.8, 50.1, 49.3, 43.5, 40.2, 38.6, 37.0, 31.5, 22.9, 22.8, 18.2. MS (ESI^−^): *m*/*z* 1049/1051 [M−H]^−^.

N-((S)-1-((4-(3-(5-chloro-2-fluoro-3-(methylsulfonamido)phenyl)-1-isopropyl-1H-pyrazol-4-yl)pyrimidin-2-yl)amino)propan-2-yl)-2-(2-(2-(4-((2-(2-((2-(2,6-dioxopiperidin-3-yl)-1,3-dioxoisoindolin-4-yl)amino)ethoxy)ethoxy)methyl)-1H-1,2,3-triazol-1-yl)ethoxy)ethoxy)acetamide (**13**).

Compound **48** (0.135 g, 0.22 mmol) was reacted with HATU (0.102 g, 0.26 mmol), DIPEA (0.034 g, 0.26 mmol) and **16** (0.135 g, 0.22 mmol) in dry DMF (3 mL) using the general procedure reported above to give **13** (0.16 g, 0.15 mmol, 67%) as a bright yellow solid. ^1^H NMR (DMSO-d*_6_*) δ: 11.06 (s, 1H), 9.86 (s, 1H), 8.56 (s, br, 1H), 8.08 (s, 1H), 7.97 (s, 1H), 7.53–7.51 (m, 2H), 7.45 (m, 1H), 7.29 (s, 1H), 7.09 (d, *J* = 8.6 Hz, 1H), 6.99 (d, *J* = 7.0 Hz, 1H), 6.67 (s, 1H), 6.56 (s, 2H), 5.01 (dd, *J* = 12.8, 5.3 Hz, 1H), 4.58–4.51 (m, 1H), 4.47–4.42 (m, 4H), 3.73 (m, 3H), 3.62–3.37 (m, 15H), 3.00 (s, 3H), 2.89–2.52 (m, 3H), 2.20 (m, 2H), 2.02–1.94 (m, 1H), 1.45 (d, *J* = 6.5 Hz, 6H), 0.83 (s, 3H). ^13^C NMR (CDCl_3_) δ: 172.6, 172.2, 170.0, 169.4, 167.8, 162.2, 160.3, 157.4, 151.0 (d, *J* = 246 Hz), 146.8, 144.8, 142.7, 136.2, 132.5, 129.4, 128.6, 128.0, 125.9 (d, *J* = 14.7 Hz), 125.3, 124.8, 124.2, 119.9, 117.0, 111.7, 110.2, 106.7, 70.8–68.9 (six partially overlapping signals), 67.2, 64.6, 54.8, 50.0, 48.9, 45.7, 43.8, 42.4, 40.2, 36.8, 31.5, 22.9, 22.8, 18.1. MS (ESI^+^): *m*/*z* 1067/1069 [MH^+^].

>14,14-dimethyl-12-oxo-3,6,9,13-tetraoxapentadecanoic acid (**49**).

BAIB (1.74 g, 5.422 mmol) and TEMPO (0.084 g, 0.54 mmol) were added to a solution of ACN/H_2_O 1:1 (5 mL) containing compound **19** (0.68 g, 2.46 mmol) The resulting mixture was stirred at room temperature until complete conversion of the starting material was observed by TLC (DCM/MeOH 9:1). The crude was purified using flash chromatography (DCM/MeOH 98:2) to give **49** (0.26 g, 0.89 mmol, 36%) as a colorless oil. ^1^H NMR (600 MHz, CDCl_3_) δ: 4.16–4.05 (m, 2H), 3.75–3.53 (m, 10H), 2.50–2.40 (m, 2H), 1.40 (dd, *J* = 3.4, 2.3 Hz, 9H). ^13^C NMR (151 MHz, CDCl_3_) δ: 173.9, 171.2, 80.8, 71.1, 70.6, 70.4, 70.3, 68.7, 66.9, 36.6, 27.9. MS (ESI^−^): *m*/*z* 291 [M−H]^−^.

2-(2-(2-(3-((2-(2,6-dioxopiperidin-3-yl)-1-oxoisoindolin-4-yl)amino)-3-oxopropoxy)ethoxy)ethoxy)acetic acid (**50**).

Compound **49** (0.078 g, 0.103 mmol) was reacted with HATU (0.12 g, 0.32 mmol), DIPEA (0.041 g, 0.32 mmol) and lenalidomide (0.07 g, 0.27 mmol) in dry DMF (2 mL) using the general procedure reported above to afford the desired protected acid as a colorless oil. This intermediate was dissolved in a solution of TFA 10% in DCM and stirred for 12 h. The solvent was evaporated under reduced pressure and the crude product was purified by flash chromatography (DCM/MeOH 95:5) to give **50** (0.095 g, 0.198 mmol, 80%) as a white solid. ^1^H NMR (600 MHz, CDCl_3_) δ 9.29 (s, br, 1H), 9.12 (s, 1H), 7.87–7.55 (m, br 1H), 7.69 (d, *J* = 7.4 Hz, 1H), 7.55 (d, *J* = 7.9 Hz, 1H), 7.44 (m, 1H), 5.16 (dd, *J* = 13.2, 4.9 Hz, 1H), 4.42 (s, 2H), 4.17 (m, 2H), 3.82–3.46 (m, 11H), 2.79–2.67 (m, 2H), 2.39–2.37 (m, 2H), 2.30–2.29 (m, 1H), 2.12–2.09 (m, 1H). MS (ESI^−^): *m*/*z* 476 [M−H]^−^.

N-((S)-1-((4-(3-(5-chloro-2-fluoro-3-(methylsulfonamido)phenyl)-1-isopropyl-1H-pyrazol-4-yl)pyrimidin-2-yl)amino)propan-2-yl)-3-(2-(2-(2-((2-(2,6-dioxopiperidin-3-yl)-1-oxoisoindolin-4-yl)amino)-2-oxoethoxy)ethoxy)ethoxy)propenamide (**14**).

Compound **50** (0.075 g, 0.16 mmol) was reacted with HATU (0.071 g, 0.17 mmol), DIPEA (0.061 g, 0.47 mmol) and **16** (0.075 g, 0.16 mmol) in dry DMF (2 mL) using the general procedure described for compounds **5–11** to give **14** (0.065g, 0.069 mmol, 45%) as a bright white solid. ^1^H NMR (600 MHz, CDCl_3_) δ 11.02 (s, 1H), 9.89 (s, br, 1H), 9.69 (s, 1H), 8.60 (s, br, 1H), 8.12 (d, *J* = 5.5 Hz, 1H), 7.73 (d, *J =* 7.6 Hz, 1H), 7.56–7.48 (m, 4H), 7.34 (s, 1H), 6.42–6.81 (m, 2H), 5.14 (dd, *J =* 13.4, 5.2 Hz, 1H), 4.58 (m, 1H), 4.37 (m, 2H), 4.12 (s, 2H), 3.74–3.92 (m, 1H), 3.67–3.44 (m, 10H), 3.13 (m, 1H), 3.05 (s, 3H), 2.89 (m, 2H), 2.66–2.55 (m, 1H), 2.43–2.30 (m, 1H), 2.24–2.23 (m, 2H), 2.04–1.95 (m, 1H), 1.49 (d, *J =* 6.9 Hz, 6H), 1.26–1.23 (m, 3H). ^13^C-NMR (151 MHz, DMSO-D6) δ 173.4, 171.6, 170.2, 168.9, 168.3, 162.7, 158.7, 151.1, 142.2, 135.4, 133.4, 133.3, 130.5, 130.4, 129.2, 128.2, 127.3 (two overlapping peaks), 126.9, 120.3, 119.9, 11.7, 70.9, 70.5, 70.2, 70.1, 70.0, 67.3, 54.4, 54.1, 52.1, 47.0, 42.3, 41.0, 36.8, 31.7, 23.1, 23.0, 18.4. MS (ESI^+^): *m*/*z* 941/943 [MH^+^].

*(S)-3-((2S,4R)-4-hydroxy-2-((4-(4-methylthiazol-5-yl)benzyl)carbamoyl)pyrrolidine-1-carbonyl)-2,2-dimethyl-5-oxo-7,10,13-trioxa-4-azahexadecan-16-oic acid* (**51**).

Compound **49** (0.030 g, 0.103 mmol) was reacted with HATU (0.046 g, 0.12 mmol), DIPEA (0.039 g, 0.31 mmol) and **4** (0.044 g, 0.103 mmol) in dry DMF (2 mL) using the same procedure described for **50** to afford the desired protected acid as a colorless oil. This intermediate was dissolved in a solution of TFA 10% in DCM and stirred for 12 h. The solvent was evaporated under reduced pressure and the crude product was purified by flash chromatography (DCM/MeOH 95:5) to give **51** (0.041 g, 0.063 mmol, 45%) as a white solid. ^1^H NMR (600 MHz, CDCl_3_) δ 8.92 (s, 1H), 7.48 (s, br, 1H), 7.35–7.32 (m, 4H), 4.74–4.41 (m, 3H), 4.32 (m, 1H), 4.11–3.97 (m, 3H), 3.76–3.50 (m, 12H), 2.53–2.52 (m, 5H), 2.33 (m, 1H), 2.18 (m, 1H), 0.98 (s, 9H). MS (ESI^−^): *m*/*z* 647 [M−H]^−^.

*(2S,4R)-1-((2S,17S)-2-(tert-butyl)-18-((4-(3-(5-chloro-2-fluoro-3-(methylsulfonamido)phenyl)-1-isopropyl-1H-pyrazol-4-yl)pyrimidin-2-yl)amino)-17-methyl-4,15-dioxo-6,9,12-trioxa-3,16-diazaoctadecanoyl)-4-hydroxy-N-(4-(4-methylthiazol-5-yl)benzyl)pyrrolidine-2-carboxamide* (**15**).

Compound **51** (0.041 g, 0.631 mmol, 1 eq) was reacted with HATU (0.030 g, 0.076 mmol, 1.2 eq), DIPEA (0.024 g, 0.19 mmol, 3 eq) and **16** (0.041 g, 0.063 mmol, 1 eq) in dry DMF (2 mL) using the general procedure described for compounds **5–11** to give **15** (0.031 g, 0.028 mmol, 44%) as a bright white solid. ^1^H-NMR (600 MHz, DMSO-D6) δ 9.9 (s, br, 1H), 8.98 (m, 1H), 8.60 (t, *J =* 5.9 Hz, 2H), 8.11 (d, *J =* 4.8 Hz, 1H), 7.49–7.4.7 (s, br, 1H), 7.45–7.43 (m, 1H), 7.42 -7.33 (m, 6H), 6.89–6.44 (m, 2H), 5.16 (d, *J =* 3.4 Hz, 1H), 4.59–4.55 (m, 2H), 4.48–4.35 (m, 3H), 4.27–4.23 (m, 1H), 3.96–3.94 (m, 2H), 3.67–3.44 (m, 15H), 3.04 (s, 3H), 2.45–2.44 (m, 3H), 2.27–2.22 (m, 2H), 1.49 (d, *J =* 6.9 Hz, 6H), 1.26–1.23 (m, 3H), 0.94–0.92 (s, 9H). ^13^C-NMR (CD_3_CN) δ 175.2, 173.0, 172.7, 171.4, 170.6, 160.9, 159.1, 151.8, 149.3, 143.2, 140.3, 132.5, 131.4, 130.5, 130.1, 129.5, 129.0, 128.8, 128.4, 127.5, 120.6, 107.5, 71.8, 71.7, 71.0–70.8 (four overlapping peaks), 67.8, 67.6, 60.3, 57.8, 57.4, 55.4, 43.3, 40.9, 37.7, 36.9, 36.7, 28.8, 23.0, 18.4, 16.4, 12.7. MS (ESI^+^): *m*/*z* 1112/1114 [MH^+^].

2,5,8,11-tetraoxatetradecan-14-oic acid (**53**).

To a stirred solution of **52** (1.14 g, 6.94 mmol) in dry THF (20 mL), NaH 60% in mineral oil (0.03 g, 0.07 mmol) was added at 0 °C. The suspension was stirred for 10 min at 50 °C. The reaction mixture was cooled to rt and *tert*-butyl acrylate (0.89 g, 6.94 mmol, 1.01 mL) was added dropwise. After 18 h of stirring at rt, the reaction was quenched by the addition of few drops of acetic acid, and the solvent evaporated under reduced pressure. The residue was treated with H_2_O (5 mL) and extracted with EtOAc (3 × 20 mL). The combined organic phases were dried, the solvent evaporated and the crude product purified by flash chromatography PE/EtOAc 1:1 to give the protected acid as a colorless oil. This intermediate was dissolved in a solution of TFA 10% in DCM (10mL) and left stirring at rt for 12 h. The solvent was evaporated and the crude product purified by flash chromatography (DCM/MeOH 95:5) to afford **53** (0.254 g; 1.07 mmol; 92%) as a bright yellow solid. ^1^H NMR (CDCl_3_) δ: 9.2 (s, br, 1H), 3.76–3.74 (m, 2H), 3.66–3.62 (m, 10H), 3.57–3.56 (m, 2H), 3.37 (dd, *J =* 4.0, 1.5 Hz, 3H), 2.62–2.60 (m, 2H). ^13^C-NMR (151 MHz, CDCl_3_) δ: 175.8, 71.9, 70.6, 70.6, 70.5, 70.3 (two overlapping peaks), 66.5, 58.9, 35.0. MS (ESI^−^): *m*/*z* 235 [M−H]^−^.

*(S)-N-(1-((4-(3-(5-chloro-2-fluoro-3-(methylsulfonamido)phenyl)-1-isopropyl-1H-pyrazol-4-yl)pyrimidin-2-yl)amino)propan-2-yl)-2,5,8,11-tetraoxatetradecan-14-amide* (**54**).

To a stirred solution of **53** (0.02 g, 0.08 mmol) in dry DMF (2 mL), HATU (0.04 g, 0.10 mmol) and DIPEA (0.02 g, 0.17 mmol) were added and the mixture left stirring at rt. After 2 h, **16** (0.05 g, 0.10 mmol) was added, and the reaction stirred at rt for a further 16 h. The solvent was evaporated under reduced pressure, the residue was treated with a 10% NaHCO_3_ solution (5 mL) and extracted with DCM (3 × 20 mL). The organic layer was washed with brine (5 mL), dried and evaporated. The crude product was purified by flash chromatography (DCM/MeOH 95/5) to give **54** (0.029 g, 0.041 mmol, 49%) as a bright yellow solid. ^1^H NMR (CDCl_3_) δ: 8.08 (s, 1H), 7.92 (s, 1H), 7.68 (s, 1H), 7.55 (dd, *J* = 6.3, 2.7 Hz, 1H), 7.45 (d, *J* = 2.4 Hz, 1H), 6.64 (s, 1H), 6.50 (d, *J* = 5.2 Hz, 1H), 4.55 (dt, *J* = 13.4, 6.7 Hz, 1H), 3.68–3.46 (m, 23H), 3.37–3.29 (m, 5H), 2.94 (s, 4H), 2.45–2.26 (m, 3H), 1.57 (dd, *J* = 9.4, 6.8 Hz, 8H), 1.17–0.88 (m, 4H). MS (ESI^−^): *m*/*z* 699/701 [M−H]^−^.

### 3.2. Biological Evaluation

#### 3.2.1. Stability Assays

Stability of compounds was determined in RPMI medium and in human serum. DMSO stock solutions of compounds (10 mM) was added to RPMI (Sigma–Aldrich, Darmstadt, Germany) competed with 10% fetal bovine serum (FBS) plus 2 mM glutamine, penicillin (100 IU/mL), and streptomycin (100 μγ/mL; Sigma–Aldrich, Darmstadt, Germany) or to human serum (sterile-filtered from human male AB plasma, Sigma–Aldrich, Darmstadt, Germany), to obtain the 100 μM final concentration with 1% of DMSO. The resulting solutions were incubated at 37 °C for 48 h. At appropriate time intervals (0, 1, 5, 24 and 48 h), 300 µL of the reaction mixture was withdrawn and added to 300 µL of acetonitrile 0.1% TFA. The samples were vortexed, sonicated for 3 min and then centrifuged for 5 min at 2500× *g*. The clear supernatant was filtered through a PTFE 0.45 μm filter (VWR) and analyzed by RP-HPLC. Each experiment was independently repeated at least three times, and results are expressed as a percentage of unmodified compound over time. The half-life was determined by fitting the data with a one phase exponential decay equation (Graph Pad, Prism software vr. 5, GraphPad Software Inc., San Diego, CA, USA).

HPLC analyses for stability assays were performed with a HP 1200 chromatograph system (Agilent Technologies, Palo Alto, CA, USA) equipped with a quaternary pump (model G1311A), a membrane degasser (G1322A) and a multiple wavelength UV detector (MWD, model G1365D) integrated in the HP1200 system. The samples were eluted on a Aquasil C18 column (200 × 4.6 mm, 5 μm), Thermo (Waltham, MA, USA). The injection volume was 20 μL (Rheodyne, Cotati, CA, USA). The mobile phase consisted of acetonitrile 0.1% TFA (solvent A) and 0.1% TFA (solvent B) at flow rate = 1.0 mL/min in gradient mode: 35% A until 5 min, from 35 to 45% A between 5 and 8 min, 45% A between 8 and 14 min, and from 45 to 35% A between 14 and 16 min.

The column effluent was monitored at 226 and 300 nm, referenced against a 800 nm wavelength. Analytical data were acquired by HP ChemStation system (Agilent Technologies). Quantitation of compounds was obtained by calibration curves (linearity determined in a concentration range of 1–100 µM, r^2^ > 0.99).

#### 3.2.2. BRAF^V600E^ Inhibition Assay

Inhibition of B-RAF (V600E) Kinase activity by synthesized compounds was measured with a B-RAF (V600E) Kinase Assay Kit purchased from BPS Bioscience using Kinase-Glo MAX^®^ (Promega Corporation, Madison, Wisconsin, USA) as detection reagent. In a 96-well plate, the different inhibitor solutions in DMSO were mixed with Buffer, ATP (10 μM) and Raf substrate. The reaction was started by adding B-RAF (V600E) enzyme (0.8 ng/μL), and the mixture was incubated at 30 °C for 45 min. After a 15 min delay to equilibrate plate temperature, 50 μL of Kinase-Glo Max was added in each well, and the luminescence was measured according to supplier’s instruction. The results, reported as IC_50_ ± S.E.M., were calculated with GrapPad Prism 7 and derive from three different experiments run in duplicate.

#### 3.2.3. Antiproliferative Activity

Cell viability was evaluated by means of CellTiter-Glo Luminescent Assay (Promega), which allows users to measure the ATP in the cell supernatant. The assay was performed according to manufacturer’s instructions. In brief, 4.10^3^ A375 or Colo205 were seeded into 96-well plates and allowed to adhere at the wall overnight. Cells were treated for 72 h with BRAF inhibitor compounds. Then, 90 µL of the cell culture medium was transferred in a white 96-well plate, to which 10 µL of CellTiter-Glo was added before measurement of luminescence. Luminescence was measured by the SPARK M10 (Tecan, Männedorf, Switzerland) plate reader.

#### 3.2.4. Western Blot Analysis

To measure the capability of our compounds to interfere with BRAF pathway, pre-treated A375 and Colo205 cells were lysed. We then performed protein analysis through Western blot by using the following primary antibodies: anti-phospho ERK 1/2 (clone E10; dilution 1:1000; Cell Signaling Technology, Danvers, MA, USA) and anti-ERK (clone 137F5; dilution 1:1000; Cell Signaling Technology); anti-phospho MEK (clone S217/221; dilution 1:1000; Cell Signaling Technology); anti-MEK1,2 (clone 9122; dilution 1:1000; Cell Signaling Technology); anti-BRAF v600e (dilution 1:1000; Abcam, Cambridge, UK); and anti-total BRAF (dilution 1:1000; ThermoFisher, Waltham, MA, USA). Vinculin (clone E1E9B; dilution 1:1000; Cell Signaling Technology) was used as a loading control. The total protein amount was determined using the bicinchoninic acid (BCA) protein assay reagent (Thermo Scientific, Waltham, MA, USA). Equivalent amounts of protein were separated by SDS–PAGE on precast Bolt 4–12% Bis-Tris gel (Invitrogen, Carlsbad, CA, USA). Proteins were then transferred from gel to nitrocellulose with a Trans-Blot Turbo transfer system (Biorad, Hercules, CA, USA), probed with goat anti-rabbit IgG (H+L) secondary antibody, HRP conjugate (Invitrogen) or goat anti-mouse IgG (H+L) secondary antibody, HRP conjugate (Jackson ImmunoResearch Inc., Langcaster, PA, USA), and detected using enhanced chemiluminescence technique (Western Lightning Plus-ECL, Enhanced Chemiluminescence Substrate; Perkin Elmer, catalog # NEL105001EA, Waltham, MA, USA).

### 3.3. Computational Studies

#### 3.3.1. Protein Modelling

BRAF in complex with the second generation BRAF inhibitor dabrafenib (PDBID: 5CSW) was chosen as the template structure for computational studies of P5B and **10**. As in PDBID: 5CSW, BRAF contains 15 mutations (in the C-lobe, and distant from the active site) and lacks an ordered A-loop, WT-BRAF sequence (Uniprot ID: P15056), manually modified with the V600E point mutation, was modelled on 5CSW structure using the SWISSMODEL webserver. The resulting BRAF^V600E^ model (hereon, BRAF^V600E^) shows DFG_in_, αC-helix_out_ and an activated unfolded A-loop. The protein was protonated at pH = 7.4 using the H++ webserver [44].

#### 3.3.2. Docking Studies

Ligands were sketched with Moldraw (Molecular Discovery Ltd., Borehamwood, UK) and converted into mol2 format using Open Babel [45]. Docking studies were performed using the GOLD software version 2021.1 (CCDC, Cambridge, UK) [46]. To verify the software’s capability of reproducing the co-crystallographic inhibitor binding pose, we extracted dabrafenib from PDBID:5CSW and docked it in BRAF^V600E^. Centroid of the docking cavity was defined as atoms at 6 Å from the dabrafenib X-ray pose; two relevant water molecules were retained in the binding site and no constraint was applied. The GOLD standard parameters were used, and the ligand was subjected to 50 genetic algorithm runs. Finally, poses were scored with the CHEMPLP function and ranked accordingly. As a good agreement between dabrafenib crystallographic pose in PDBID: 5CSW and dabrafenib docking pose in BRAF^V600E^ (RMSD_heavy_ = 0.563 Å) was observed, BI 882730 and encorafenib warheads were docked using the same parameters and protocol. Top-ranked docking poses of BI 882730 and encorafenib in BRAF^V600E^ were exported for subsequent MD simulations. Importantly, the linker and pomalidomide moieties were not included in the previous docking studies to avoid artifact interactions that could bias the subsequent MD simulation. The linker and pomalidomide segment of P5B was sketched with Moldraw, minimized and converted to mol2 with OpenBabel. It was then attached to the docked warheads, thereby rebuilding P5B and **10** in the binding site and obtaining **BRAF:P5B** and **BRAF:10** systems.

#### 3.3.3. MD Setup, Simulations and Analysis

Before the MD production, P5B and **10** conjugates were parameterized using the DFT-based protocol implemented in BiKi software suite (http://www.bikitech.com/ accessed on 15 July 2022). **BRAF:P5B** and **BRAF:10** systems were embedded in an octahedral box and solvated in water, with addition of 0.15 M NaCl, using AmberTools22, the Amber14SB forcefield in BiKi, and the GROMACS 2022.1 engine for MD simulations. The LINCS algorithm was employed to fix the bond length between hydrogens and heavy atoms at the equilibrium distance. Periodic boundary conditions (PBC) were set, and long range electrostatic effects were adjusted with the Particle-Mesh Ewald (PME) method; a cut-off value of 12 Å was fixed for both electrostatic and van der Waals interactions. Three replicates were set up for each system, as follows. The **BRAF:P5B** and **BRAF:10** complexes were minimized in two steps, using the steepest descendant and conjugate gradient algorithms; subsequently, six thermalization steps in the isothermal-isochoric ensemble (NVT) were performed for heating the system from 0 to 300 K, with a temperature ramp of 50 K every 0.1 ns, and coordinate constraints for protein backbone and ligands during heating from 0 to 200 K. Two final equilibration steps in the isothermal-isobaric canonical (NPT) ensemble were carried out before MD production, the first imposing position restraints to the protein backbone and ligands, and the second with no restraints. Finally, the unbiased MD production was carried out for 500 ns (timestep = 2 fs). Three independent replicas were obtained for **BRAF:P5B** and three for **BRAF:10** (total simulated time = 3 μs). Analyses on trajectories were carried out using GROMACS built-in functions, pictures and movies were generated with PyMOL, and data were plotted using the Matplotlib library.

## 4. Conclusions

In this work, we synthesized a series of potential encorafenib-based PROTACs targeting the oncogenic BRAF^V600E^ variant using diverse linkers and E3-ligase moieties. Our heterobifunctional compounds showed inhibition of BRAF^V600E^ mutant, even with a two-to-four-fold decrease in potency with respect to encorafenib, with no differences depending on the nature of the linker or of the E3 ligase ligand. Furthermore, we assessed the antiproliferative activity in A375 and Colo205 cell lines, comparing our conjugates with encorafenib and with P5B, an effective BRAF^V600E^ degrader published by Posternak et al. [24]. Overall, our compounds showed a higher IC_50_ with respect to encorafenib; however, compounds **10** and **11** showed an antiproliferative activity comparable to P5B. To assess the protein target degradation efficiency of our best antiproliferative compounds **10** and **11** with respect to P5B, a Western blot analysis was carried out after a 24-h incubation in A375 and Colo205 cell lines. As expected, **10**, **11** and P5B decreased the phosphorylation of MEK and ERK, as a consequence of BRAF^V600E^ inhibition. However, in stark contrast with the results shown by the effective P5B BRAF^V600E^ degrader, our compounds **10** and **11** were not able to induce target protein degradation. To generate hypotheses that might explain such discrepant behavior, we ran molecular docking and molecular dynamics simulations, comparing P5B and **10** (bearing the same linker and E3 ligase ligand as P5B). Our in silico results suggest that the linker attachment point on the kinase inhibitor warhead affects the orientation and degrees of freedom of the linker and pomalidomide moieties. As no significant differences in solvent exposure of P5B and **10** were recorded, we hypothesize that a ternary complex can be stabilized by both PROTACs. However, as the BRAF^V600E^ surface areas accessed by the P5B and **10** during MD simulations are different, we hypothesize that the protein–protein interface might also be different, and productive in the case of P5B, while unproductive in the case of **10**. Our hypothesis is strengthened by the literature data reporting inactive dabrafenib-based BRAF degraders that present an analogous linker attachment point [24]. Further work would be required to clarify the structure of productive BRAF^V600E^/E3 ligase interactions, for better rationalizing the inactivity of encorafenib- and dabrafenib-based PROTACs and for enhancing the design of novel effective BRAF degraders. In a broader context, the problem of predicting protein degradation efficiency is currently being addressed by a growing body of research, and recent works point out the importance of coupling in silico and experimental methodologies for modelling such a complex and multifactorial process.

## Figures and Tables

**Table 1:** Antiproliferative activity of encorafenib (**1**) and synthesized encorafenib conjugates **5–15** in melanoma A375 cell line.

Compound	Antiproliferative Activity ^a^
	IC_50_ ± SEM (nM)
**5**	2744 ± 71
**6**	406.1 ± 2.4
**7**	520.1 ± 80.5
**8**	1756 ± 287
**9**	2003.5 ± 31.8
**10**	132.2 ± 1.95
**11**	389.2 ± 15.1
**12**	732 ± 16
**13**	4095 ± 71.5
**14**	1046 ± 0.75
**15**	3380 ± 171.5
encorafenib (**1**)	<50 ^b^

^a^ After 72 h of treatment, a luminescent assay was performed to determine the number of viable cells on total cells. Mean ± SEM is representative of three independent experiments. ^b^ reported IC_50_ for **1** in BRAF^V600E^ mutant A375 cell line is 4 nM [20].

## Data Availability

Data are available upon request from the authors.

## Supplementary Materials

The following supporting information can be downloaded at: https://www.mdpi.com/article/10.3390/molecules27238513/s1. HPLC trace for hydrolysis of encorafenib (Figure S1). Concentration–response curves for the antiproliferative activity of **1**, **5**–**15** in A375 cells (Figure S2). Structures of P5B and BI 882370 (Figure S3). Western blot analysis of protein expression after treatment of A375 and Colo205 cell lines with compounds **10**, **11** and P5B at 3–10 µM (Figure S4). Cell penetration of encorafenib (**1**) and compound **8** (Table S1). Solvent-accessible surface area (SASA) for P5B and **10** along MD simulation time (Figure S5). High occupancy residues in **P5B:BRAF** complex (Figure S6). Persistence of hydrogen bond pairs between BRAF^V600E^ and **10** (Table S2) and BRAF^V600E^ and P5B (Table S3). HPLC purity of target compounds **5**–**15** (Table S4). HPLC chromatograms of final compounds **5**–**15** (Figure S7). SI-movies.

## Sample Availability

Samples of the compounds are available upon request from the authors.
