# Supplementary material for: Investigation into the Use of Encorafenib to Develop Potential PROTACs Directed against BRAFV600E Protein
Source: Molecules. 2022 Dec 3;27(23):8513. doi: 10.3390/molecules27238513 (PMC9736157; doi:10.3390/molecules27238513)
Supplement: Supplementary file 1 [file molecules-27-08513-s001.zip › molecules-2063849-supplementary.pdf]

molecules

MDPI

Article

Investigation into the use of encorafenib to develop potential PROTACs directed against $BRAF^{V600E}$ protein

Elisabetta Marini [1], Marco Marino [1], Giulia Gionfriddo [2], Federica Maione [2], Marta Pandini [2], Daniele Oddo [2], Marta Giorgis [1], Barbara Rolando [1], Federica Blua [1], Simone Gastaldi [1], Serena Marchiò [2,3], Sandra Kovachka[1,4], Francesca Spyrakis [1], Eleonora Gianquinto [1,*] Federica Di Nicolantonio [2,*] and Massimo Bertinaria [1,*]

[1] Department of Drug Science and Technology, University of Turin, Via Giuria 9, 10125, Torino, Italy; elisabetta.marini@unito.it (E.M.), marino.marco993@gmail.com (M.M.), marta.giorgis@unito.it (M.G.), barbara.rolando@unito.it (B.R.), simone.gastaldi@unito.it (S.G.), federica.blua@unito.it (F.B.), francesca.spyrakis@unito.it (F.S.)
[2] Candiolo Cancer Institute, FPO - IRCCS, Candiolo (TO), Italy;
[3] Department of Oncology, University of Torino, Candiolo (TO), Italy; giu.gionfriddo@gmail.com (G.G.), marta.pandi94@gmail.com (M.P.); federica.maione@unito.it (F.M.); danieleoddo@hotmail.it (D.O.); serena.marchio@unito.it (S.M.)
[4] Université Côte d'Azur, CNRS, ICN, 28 Avenue Valrose, 06108 Nice, CEDEX 2, France; sandra.kovachka@etu.univ-cotedazur.fr (S.K.)
* Correspondence: massimo.bertinaria@unito.it (M.B.); Tel.: +39 011 6707146; eleonora.gianquinto@unito.it (E.G.); Tel: +39 011 6707185; federica.dinicolantonio@unito.it (F.D.N.); Tel: +39 011 9933827

Citation: Lastname, F.; Lastname, F.; Lastname, F. Title. *Molecules* **2022**, *27*, x. https://doi.org/10.3390/xxxxx

Academic Editor: Firstname Lastname

Received: date
Accepted: date
Published: date

Publisher's Note: MDPI stays neutral with regard to jurisdictional claims in published maps and institutional affiliations.

Copyright: © 2022 by the authors. Submitted for possible open access publication under the terms and conditions of the Creative Commons Attribution (CC BY) license (https://creativecommons.org/licenses/by/4.0/).

Supplementary Materials
Content

Figure S1: Hydrolysis of encorafenib under different conditions.

Figure S2: Concentration-response curves for the antiproliferative activity of **1**, **5-15** in A375 cells.

Figure S3: Structures of P5B and BI 882370.

Figure S4. Western blot analysis of protein expression after treatment of A375 and Colo205 cell lines with compounds **10**, **11** and P5B (3-10 μM).

Table S1: Cell penetration of encorafenib (**1**) and compound **8**.

Figure S5. Solvent-accessible surface area (SASA) for P5B and **10** along MD simulation time (ns).

Figure S6. High occupancy residues in **P5B:BRAF** complex.

Table S2. Persistence of hydrogen bond pairs between $BRAF^{V600E}$ and **10**.

Table S3. Persistence of hydrogen bond pairs between $BRAF^{V600E}$ and P5B.

Table S4. HPLC analysis of target compounds **5-15**.

Figure S7. HPLC chromatograms of final compounds **5-15**.

NMR spectra of final compounds **5-15**.

Figure S1. Hydrolysis of encorafenib under different conditions.

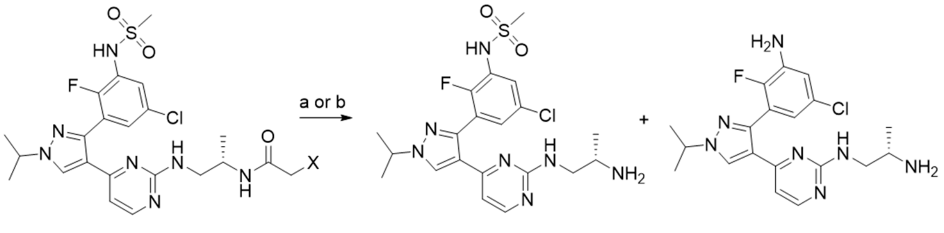

1 **16** **17**

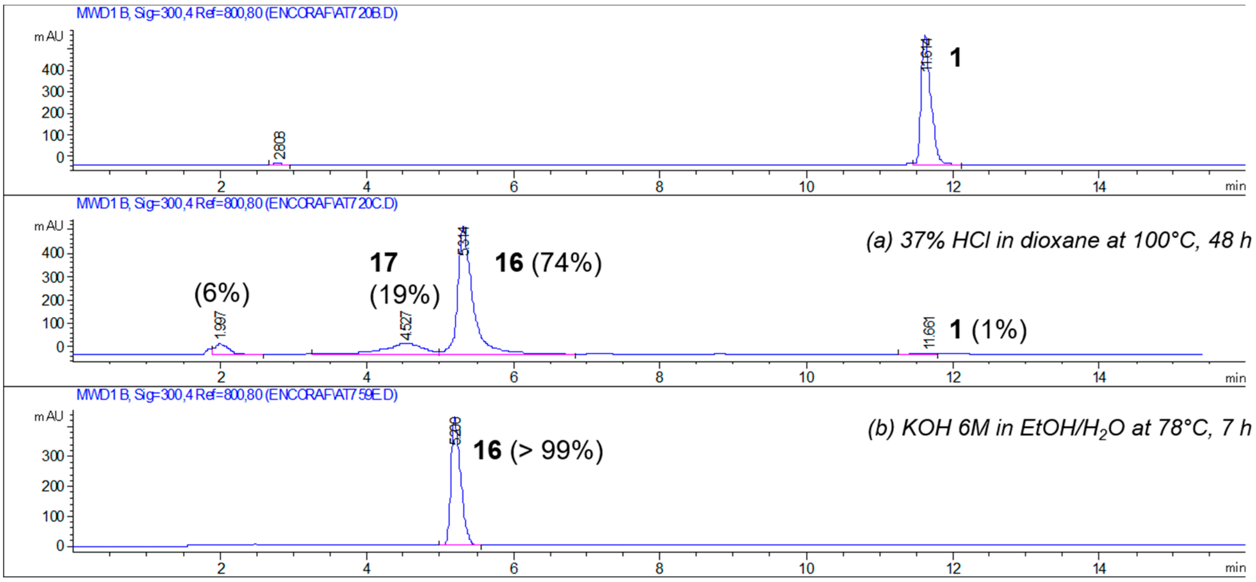

Figure S2. Concentration-response curves fot the antiproliferative activity of **1**, **5-15** in A375 cells.

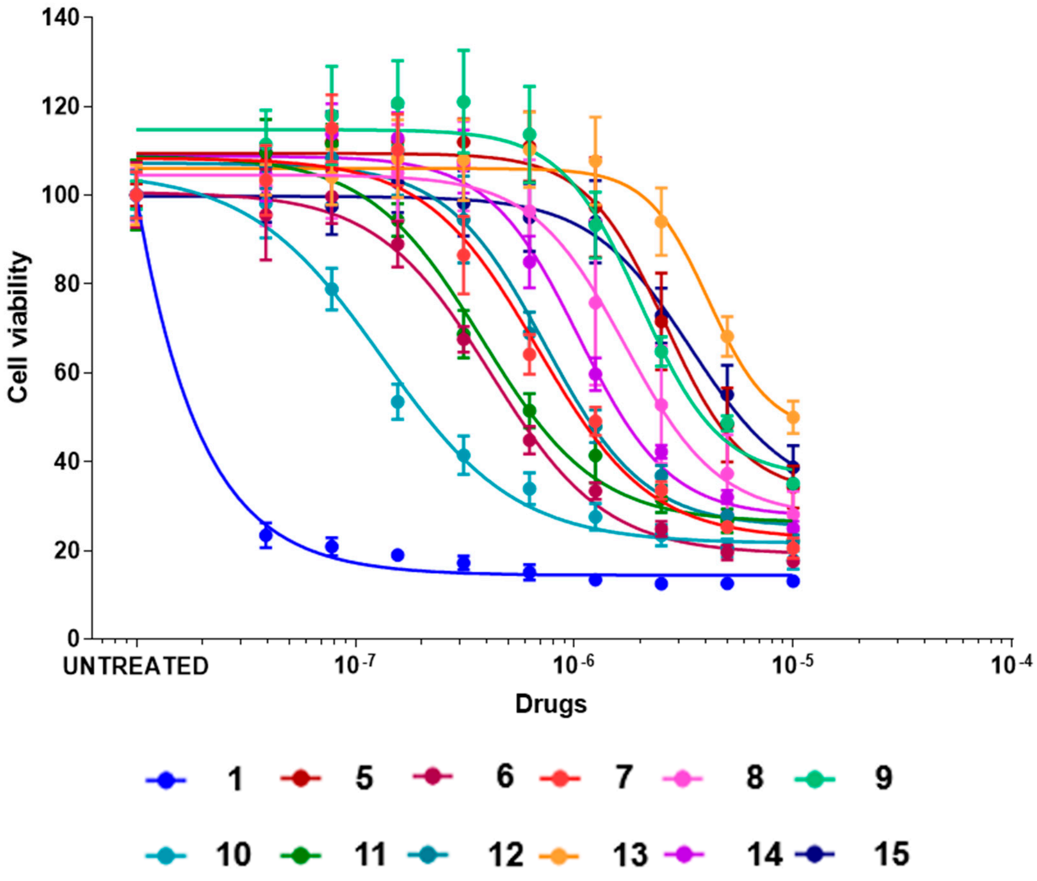

Figure S3. Structures of P5B and BI 882370.

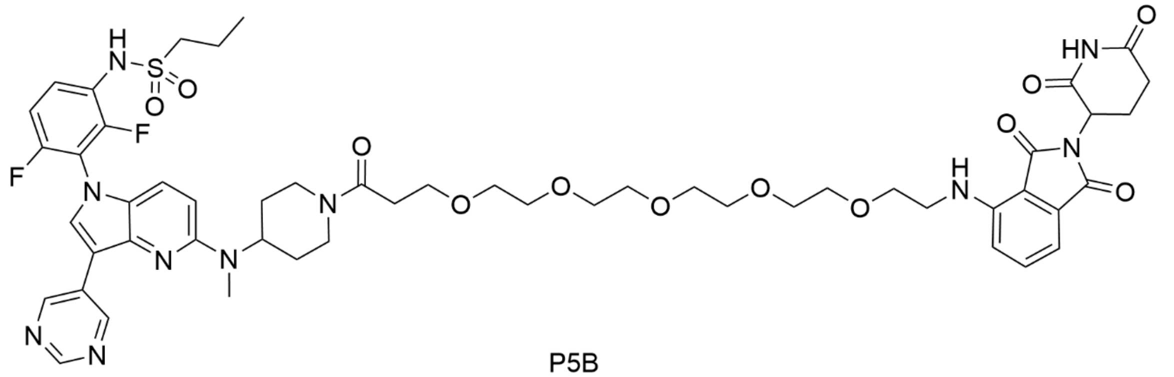

P5B

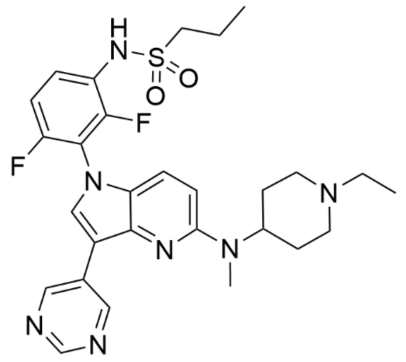

BI 882370

Figure S4. Western blot analysis of protein expression after treatment of A375 and Colo205 cell lines with compounds **10**, **11** and P5B (3-10 µM).

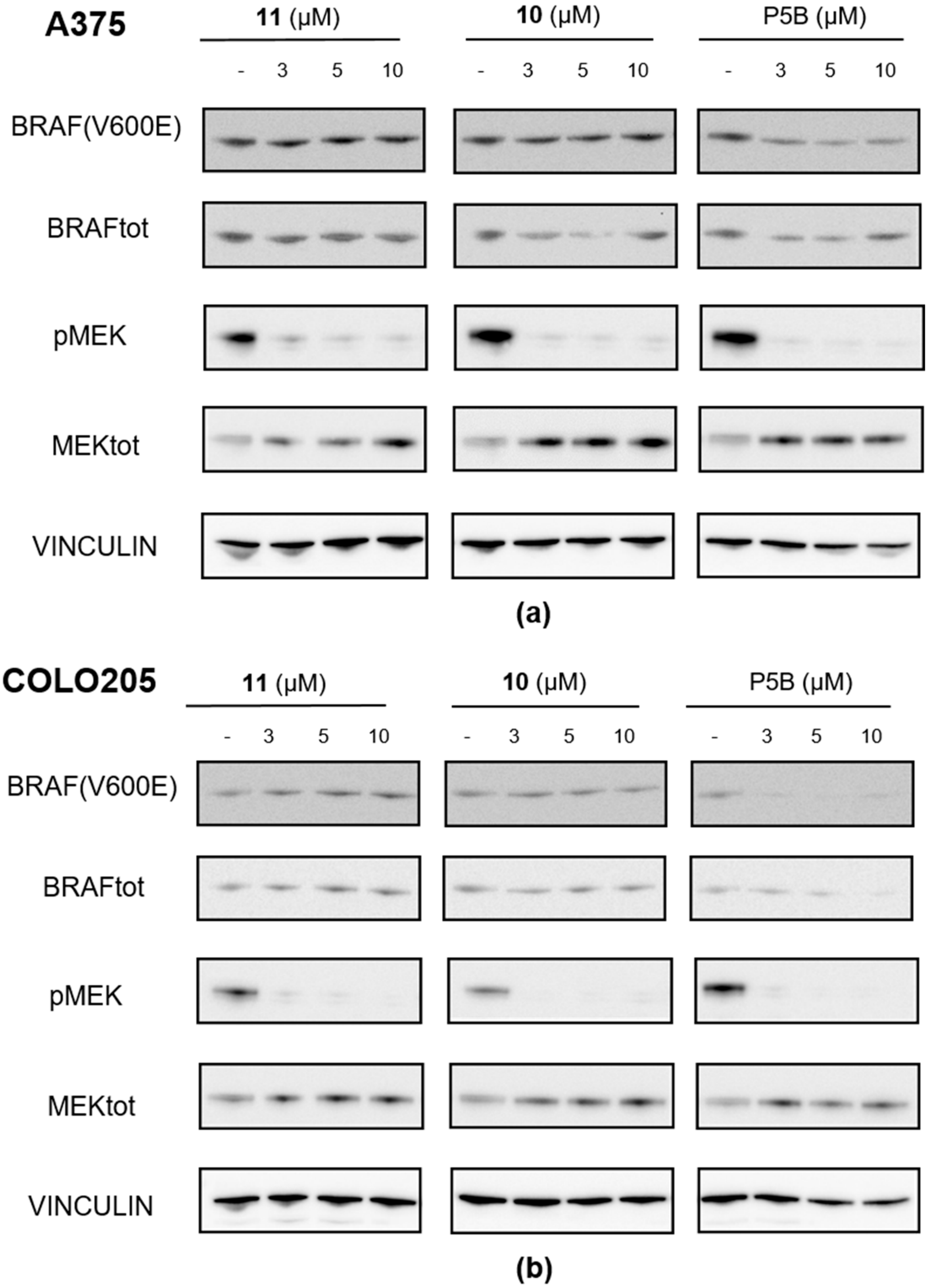

Table S1. Cell penetration of encorafenib (**1**) and compound **8** after 4 h of incubation in Colo205 cell line.

	Encorafenib (**1**) [a]	compound **8** [b]
Medium (μM)	8.6 ± 0.3	36 ± 1
Membrane (nmol/mg protein)	0.093 ± 0.005	0.12 ± 0.01
Citosol (nmol/mg protein)	0.28 ± 0.05	0.37 ± 0.18

[a] incubated at 10 μM concentration; [b] incubated at 50 μM concentration.

The cell penetration of compound **8**, used as representative compound of the synthesized series, and reference encorafenib was assessed by incubating respectively 50 μM and 10 μM solutions of each compound with Colo205 cells. The cytosolic and membrane proteins fractions were separated after 4 h of incubation and the amount of each compound in the different cell compartments and incubation medium was measured by HPLC.

Experimental

Colo205 cells (20x10^6) were grown in 100-mm diameter Petri dishes and incubated for 4 h in fresh medium containing the tested compounds. Membrane proteins and cytosolic cellular fraction were separated by ProteoExtract® Transmembrane Protein Extraction Kit, following the producer's instructions. A 50 μL aliquot of each fraction was used for protein content measurements; the remainder was stored at -80°C until analysis. The quantity of tested compounds in transmembrane protein and cytosolic fractions was measured by HPLC.

HPLC analyses were performed with a HP 1200 chromatograph system (Agilent Technologies, Palo Alto, CA, USA) equipped with a quaternary pump (model G1311A), a membrane degasser (G1322A), a multiple wavelength UV detector (MWD, model G1365D) integrated in the HP1200 system. Data analysis was performed using a HP ChemStation system (Agilent Technologies). The sample was eluted on AQUASIL C18 column (200 × 4.6 mm, 5 μm, Thermo Electron Corporation, UK). The injection volume was 20 μL (Rheodyne, Cotati, CA). The mobile phase consisting of acetonitrile 0.1% TFA (solvent A) and water 0.1% TFA (solvent B) at flow-rate = 1.0 mL/min with gradient conditions: 35% A until 5 min, from 35 to 45% A between 5 and 8 min, 45% A between 8 and 20 min, and from 45 to 35% A between 20 and 25 min. The column effluent was monitored at 226, 270 and 400 mn referenced against a 800 nm wavelength. Data analysis was performed with Agilent ChemStation. Quantitation of compounds was done interpolating the peak area of compounds in a calibration curve obtained using standard solutions in a concentration range of 1.0 μM to 50 μM ($r^2 > 0.995$).

Figure S5. Solvent-accessible surface area (SASA) for P5B and **10** along MD simulation time (ns). The three replicas are plotted with different colors.

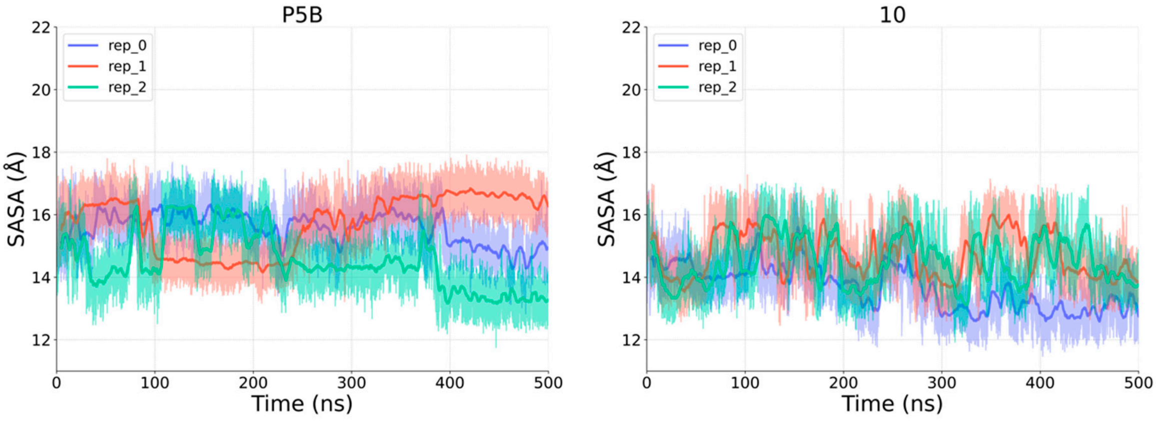

Figure S6. High occupancy residues in **P5B:BRAF** complex. Occupancy is defined as the normalized number of MD frames in which atoms of the ligand are at a distance equal or lower than 4 Å from protein atoms. Occupancy is shown as a range of colors spanning from blue (low occupancy) to orange (high occupancy) areas. A black ellipsoidal shape highlights residues of $BRAF^{V600E}$ which were accessed by P5B but not by **10**. Protein is represented as surface, ligand is depicted as sticks and labelled.

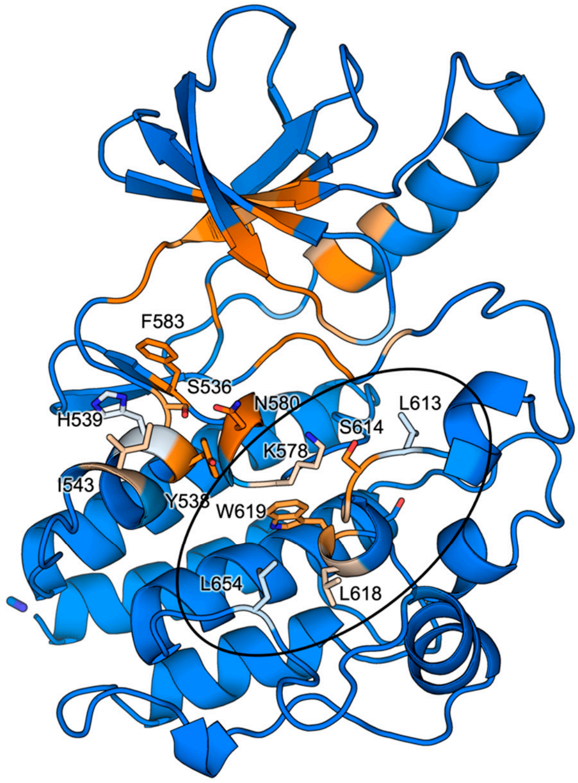

Table S2. Persistence of hydrogen bond pairs between $BRAF^{V600E}$ and **10**.

**$BRAF^{V600E}$-10 H-bond pairs**	**H-bond persistence (%)***		
	**Replica 1**	**Replica 2**	**Replica 3**
532Cys(H) - **10**(N14)	92.2	97.5	96.9
483Lys(HZ1) - **10**(O24)	84.1	95.4	99.0
596Gly(H) - **10**(O25)	-	56.9	-

* Only relevant values (≥20%) have been reported

H-bond persistence is expressed as the percentage of simulation time in which the distance between the donor (D-H) and acceptor (A) is lower than 3.5 Å, and the angle ADH is lower or equal to 30°. Protein residues are numbered and reported with the three-letter code, the acceptor or donor atom names are enclosed in curly brackets.

Table S3. Persistence of hydrogen bond pairs between $BRAF^{V600E}$ and P5B.

**$BRAF^{V600E}$-P5B H-bond pairs**	**H-bond persistence (%)***		
	**Replica 1**	**Replica 2**	**Replica 3**
595Phe(H) - P5B(O28)	86.3	-	-
594Asp(H) - P5B(O28)	45.5	-	-
594Asp(H) - P5B(O26)	12.4	-	81.4
580Asn(D21) - P5B(O41)	43.7	29.2	28.1
532Cys(H) - P5B(N20)	95.6	96.6	30.4
597Leu(H) - P5B(O26)	-	75.5	-
580Asn(D21) - P5B(O43)	-	22.3	-
483Lys(HZ1) - P5B(N24)	-	77.9	64.1
465Ser(H) - P5B(O69)	-	24.9	-

* Only relevant values (≥20%) have been reported

H-bond persistence is expressed as the percentage of simulation time in which the distance between the donor (D-H) and acceptor (A) is lower than 3.5 Å, and the angle ADH is lower or equal to 30°. Protein residues are numbered and reported with the three-letter code, the acceptor or donor atom names are enclosed in curly brackets.

Table S4. HPLC analysis of target compounds **5-15**.

Purity of compounds

The purity of target compounds was checked by RP-HPLC. Analyses were performed on a HP1100 chromatograph system (Agilent Technologies, Palo Alto, CA, USA) equipped with a quaternary pump (G1311A), a membrane degasser (G1379A), a diode-array detector (DAD) (G1315B) integrated in the HP1100 system. Data analysis were processed by HP ChemStation system (Agilent Technologies). The analytical column was a LiChrospher® 100 C18-e (250×4.6mm, 5μm) (Merck KGaA, 64271 Darmstadt, Germany) eluted with acetonitrile/ 0.1% TFA in a ratio depending on the characteristics of the compound. All compounds were dissolved in the mobile phase at a concentration of about 0.1 mg/ml and eluted with a flow rates of 1.0 mL min^{-1}; the column effluent was monitored at 226, 254 and 300 nm referenced against 800 nm. The purity of the compounds was evaluated as a percentage ratio between the areas of the main peak and of possible impurities at the three wavelengths and also using DAD purity analysis of the chromatographic peak.

Purity of compounds evaluated by RP-HPLC (DAD detector): the lowest purity value among those acquired at the different wavelengths is reported.

compound	% purity
**5**	100
**6**	99
**7**	98
**8**	100
**9**	98
**10**	96
**11**	98
**12**	97
**13**	100
**14**	99
**15**	97

Figure S7. HPLC chromatograms of final compounds **5-15**.

Compound **5**

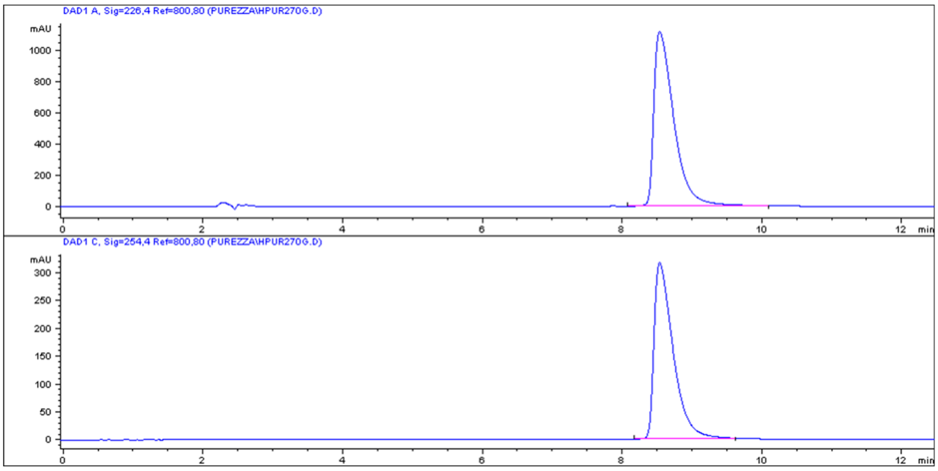

Compound **6**

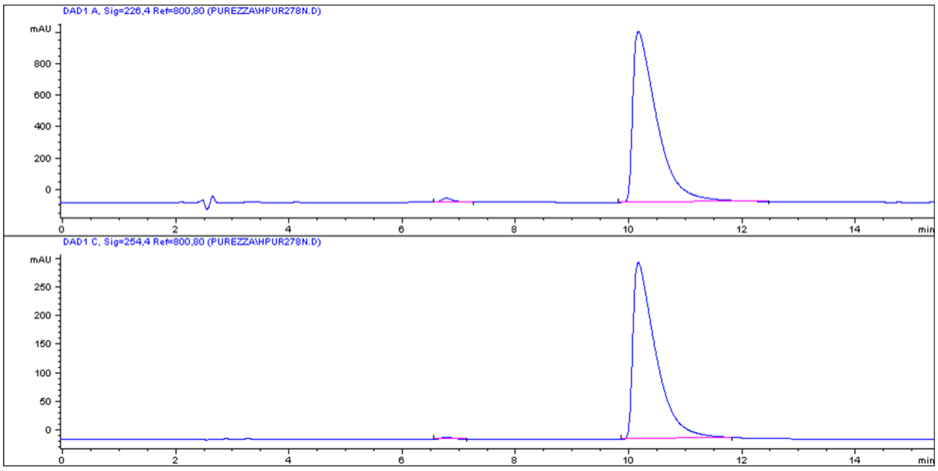

Compound **7**

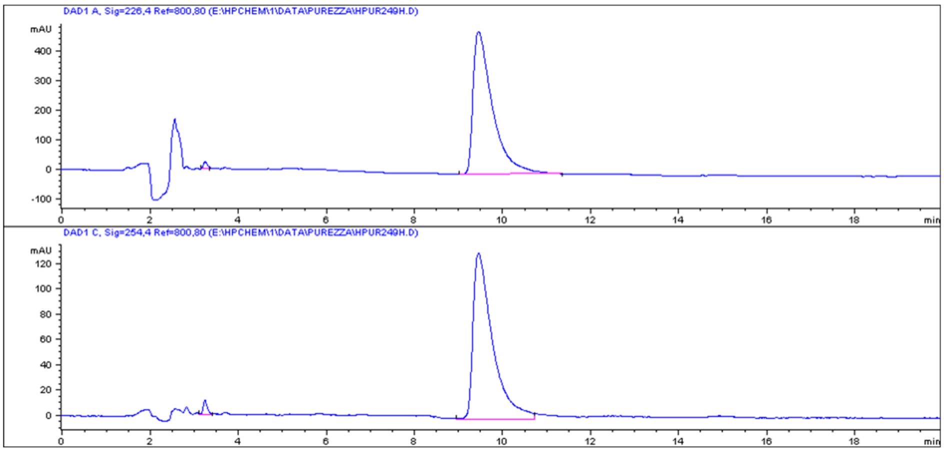

Compound **8**

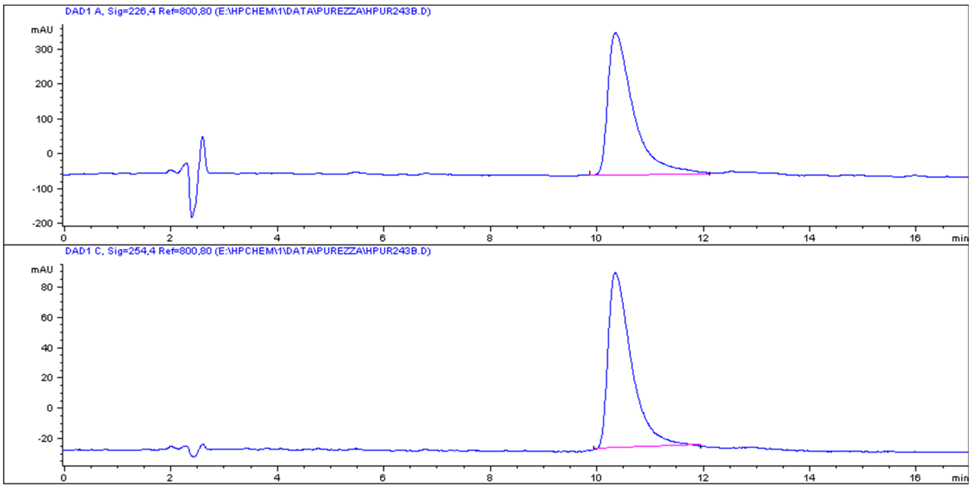

Compound **9**

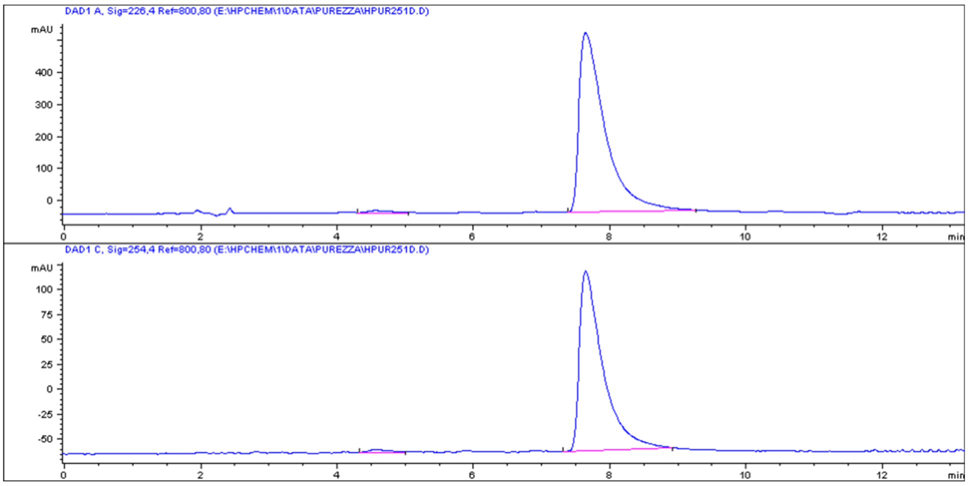

Compound **10**

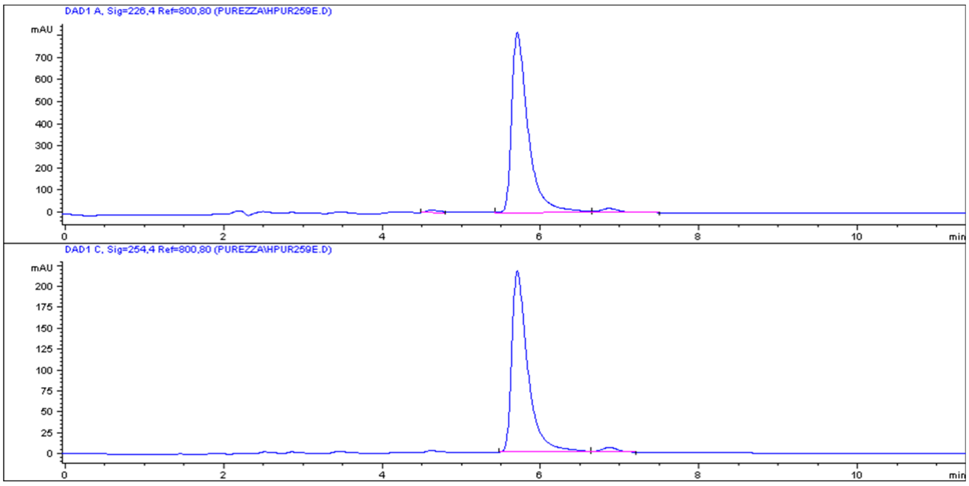

Compound **11**

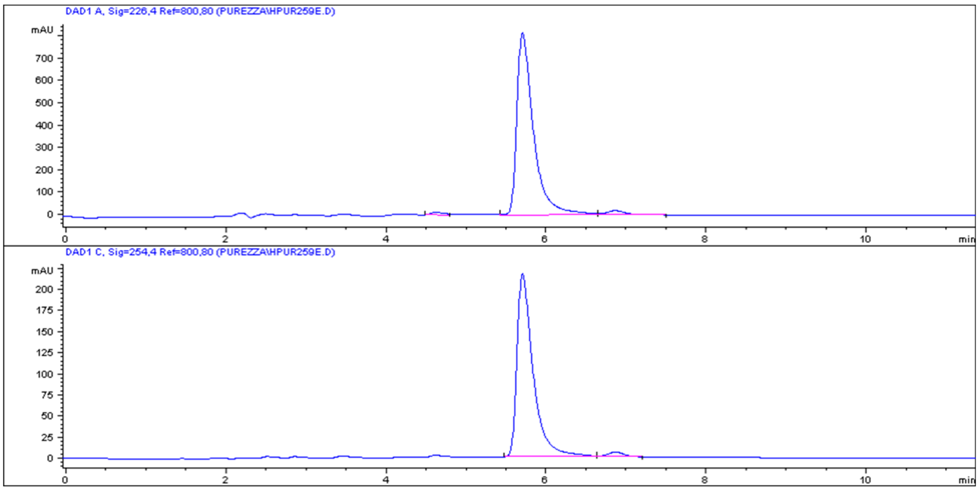

Compound **12**

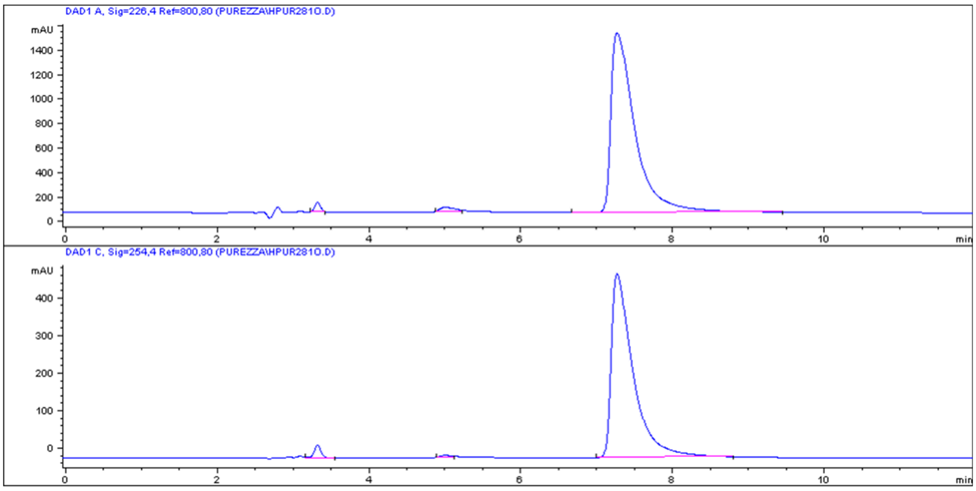

Compound **13**

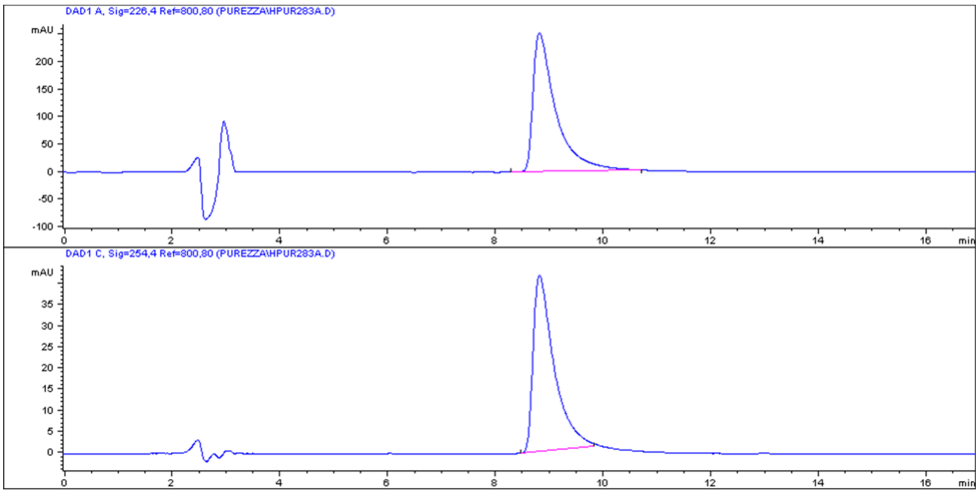

Compound **14**

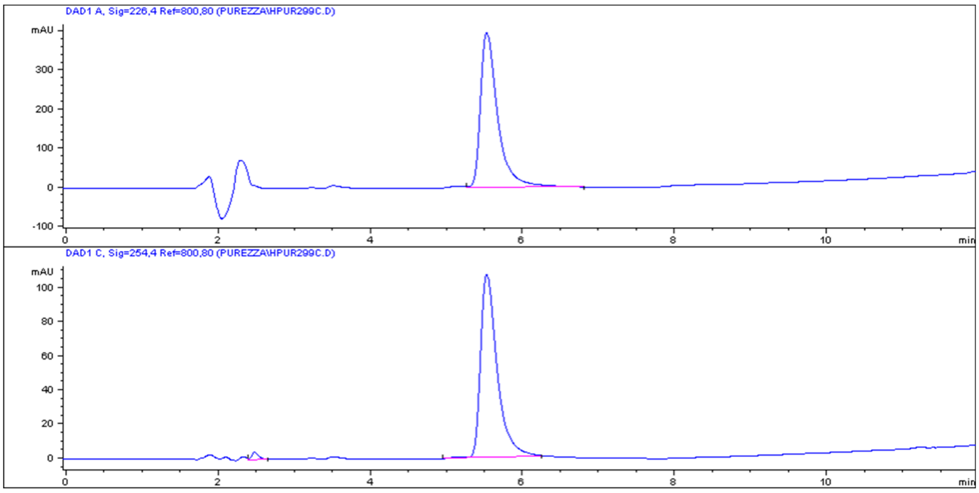

Compound **15**

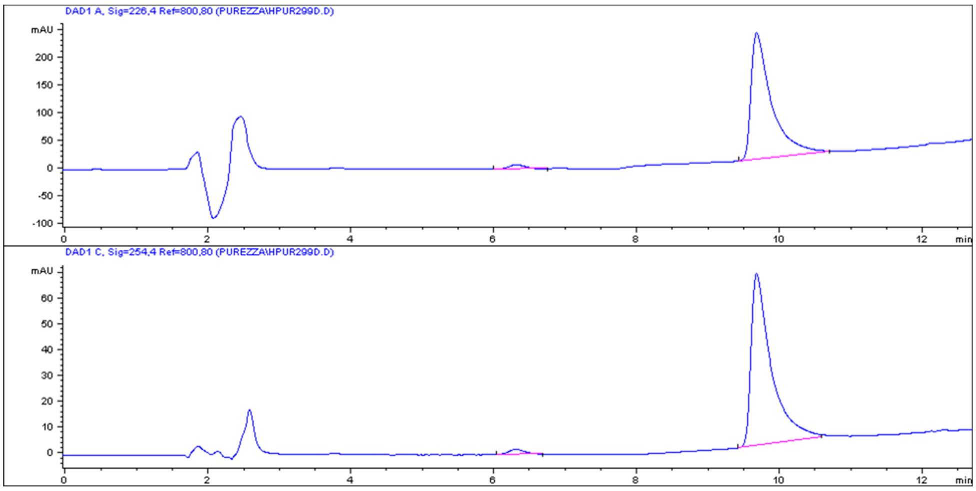

NMR spectra of final compounds **5-15**

Compound **5**

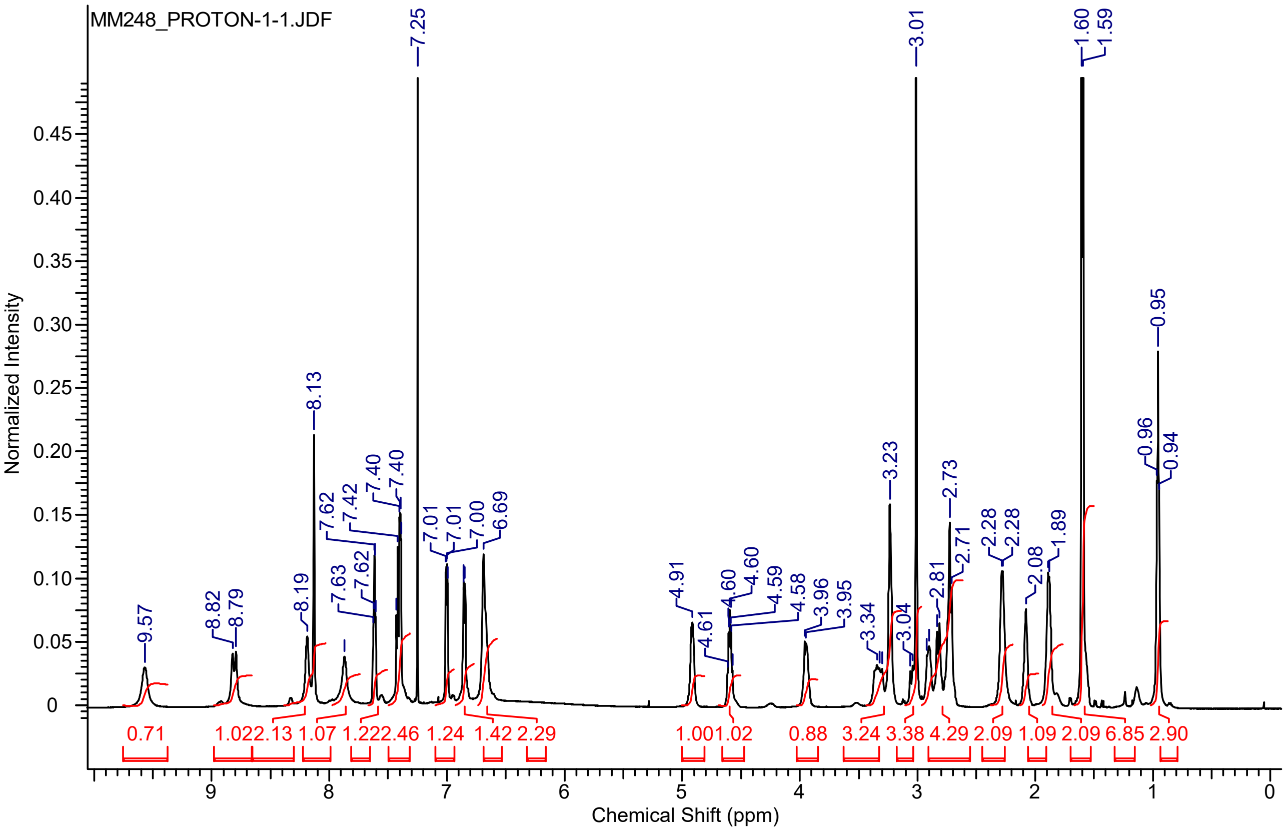

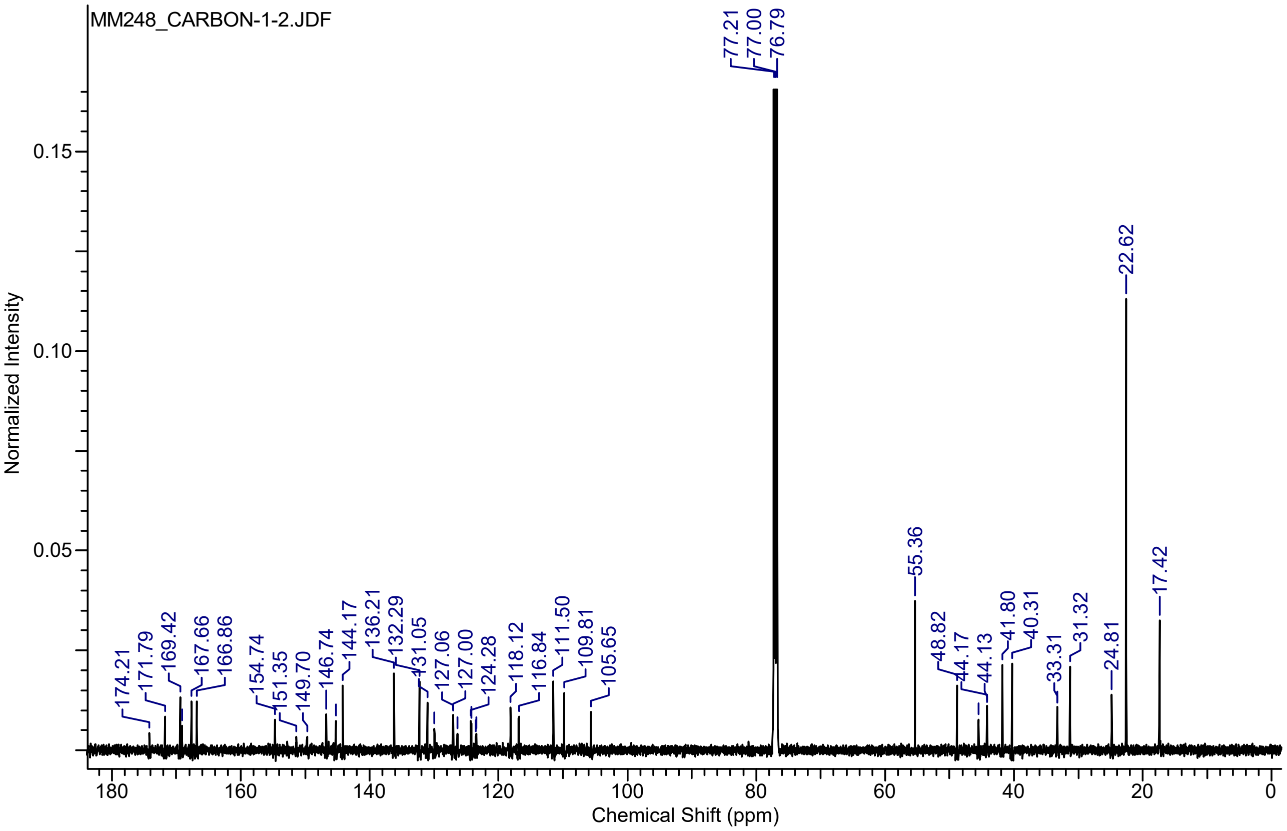

Compound **6**

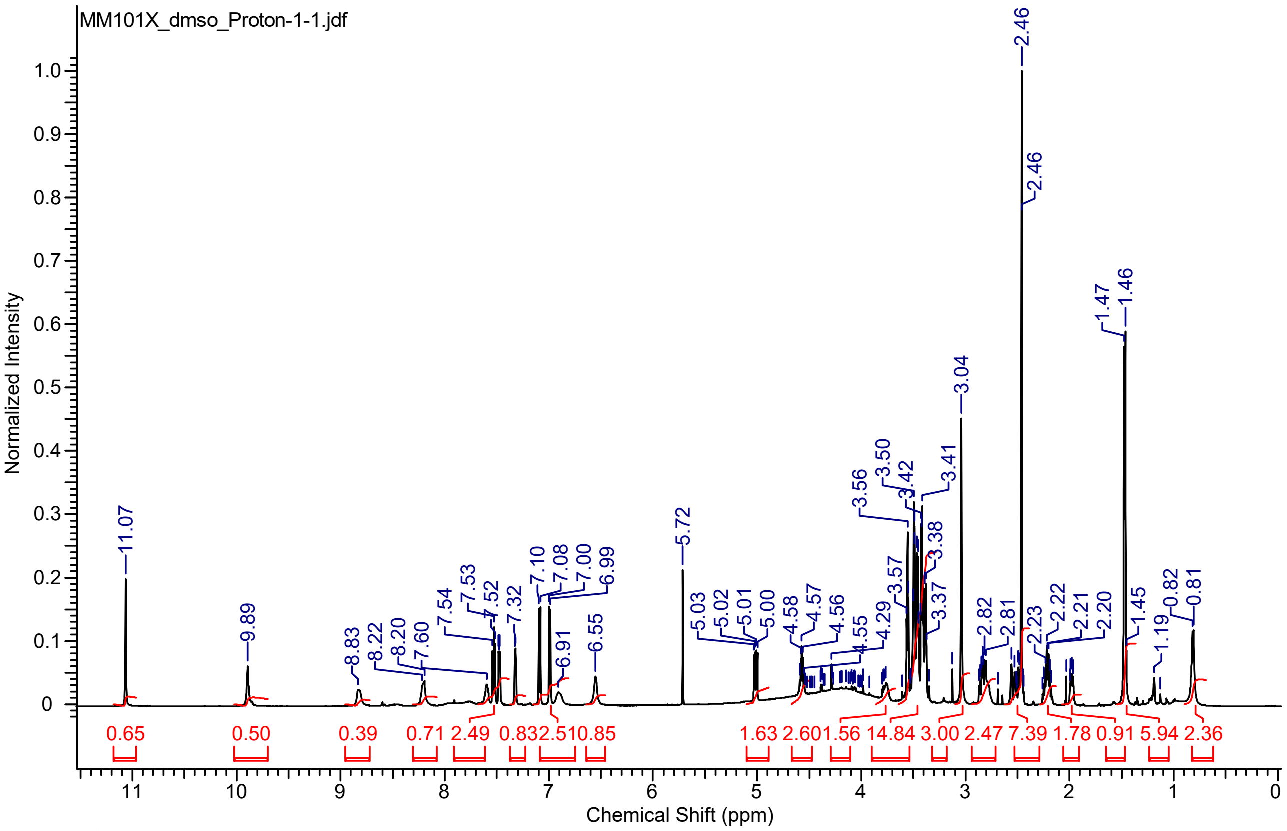
MM101X_dmso_Proton-1-1.jdf
Normalized Intensity
Chemical Shift (ppm)
0.65 0.50 0.39 0.71 2.49 0.83 2.51 0.85 1.63 2.60 1.56 14.84 3.00 2.47 7.39 1.78 0.91 5.94 2.36

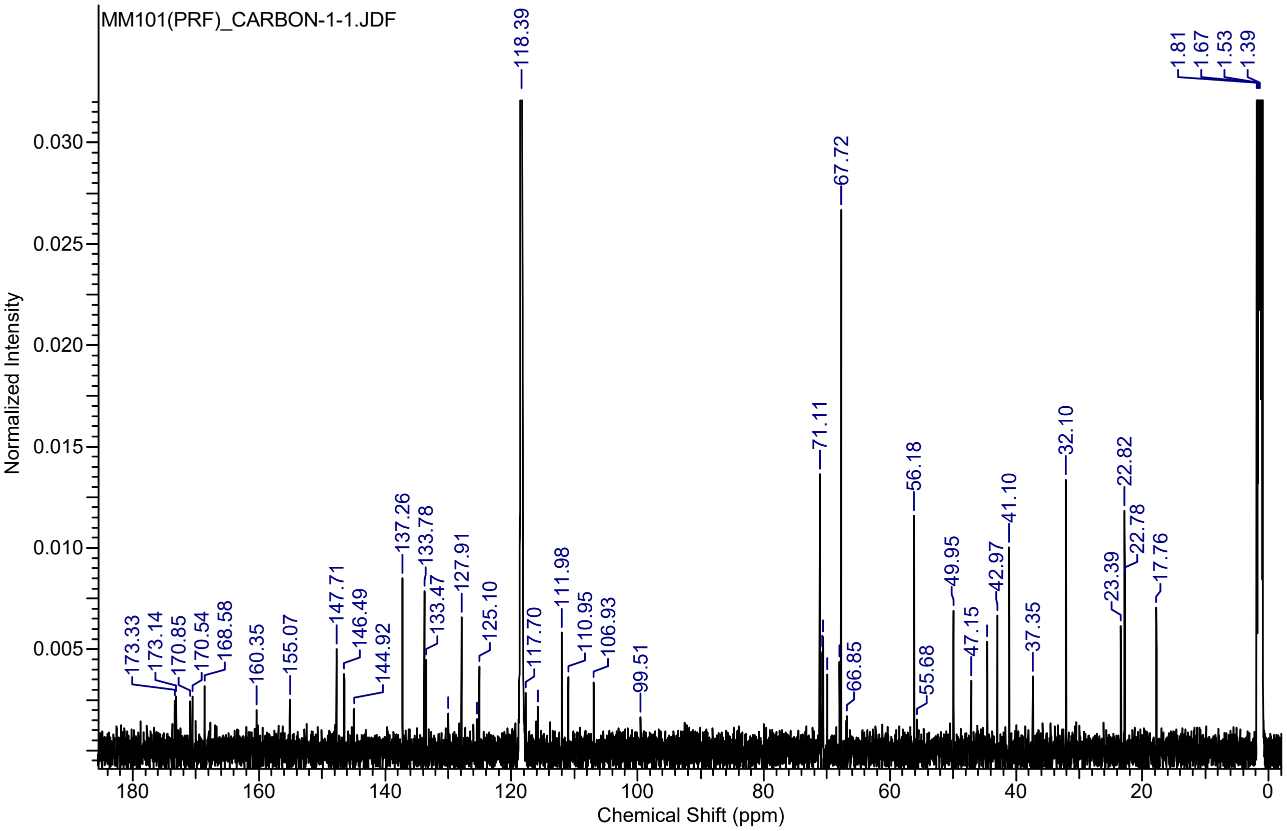
MM101(PRF)_CARBON-1-1.JDF
Normalized Intensity
Chemical Shift (ppm)

Compound **7**

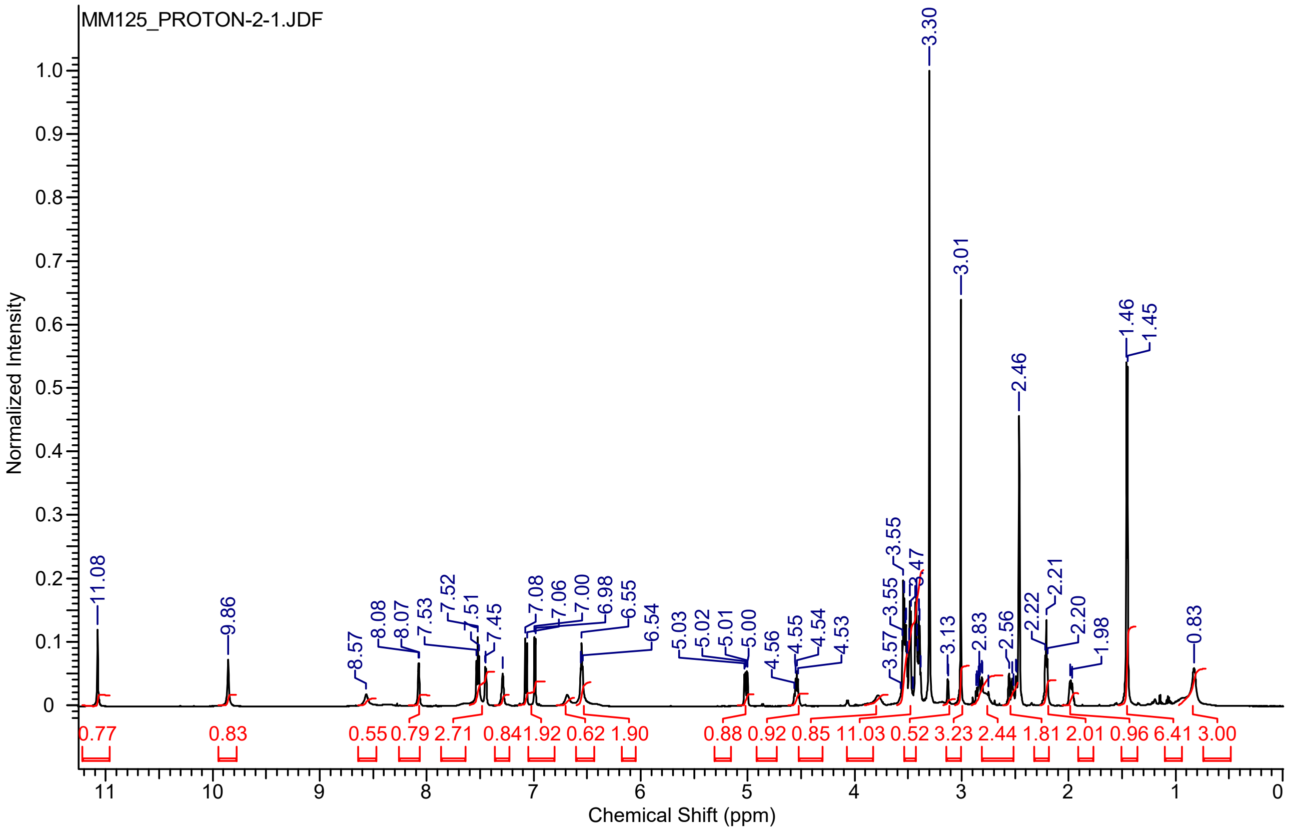
MM125_PROTON-2-1.JDF
Normalized Intensity
Chemical Shift (ppm)
11.08
9.86
8.57
8.08
8.07
7.53
7.52
7.51
7.45
7.08
7.06
7.00
6.98
6.55
6.54
5.03
5.02
5.01
5.00
4.56
4.55
4.54
4.53
3.57
3.55
3.55
3.47
3.30
3.13
3.01
2.83
2.56
2.46
2.22
2.21
2.20
1.98
1.46
1.45
0.83
0.77 0.83 0.55 0.79 2.71 0.84 1.92 0.62 1.90 0.88 0.92 0.85 11.03 0.52 3.23 2.44 1.81 2.01 0.96 6.41 3.00

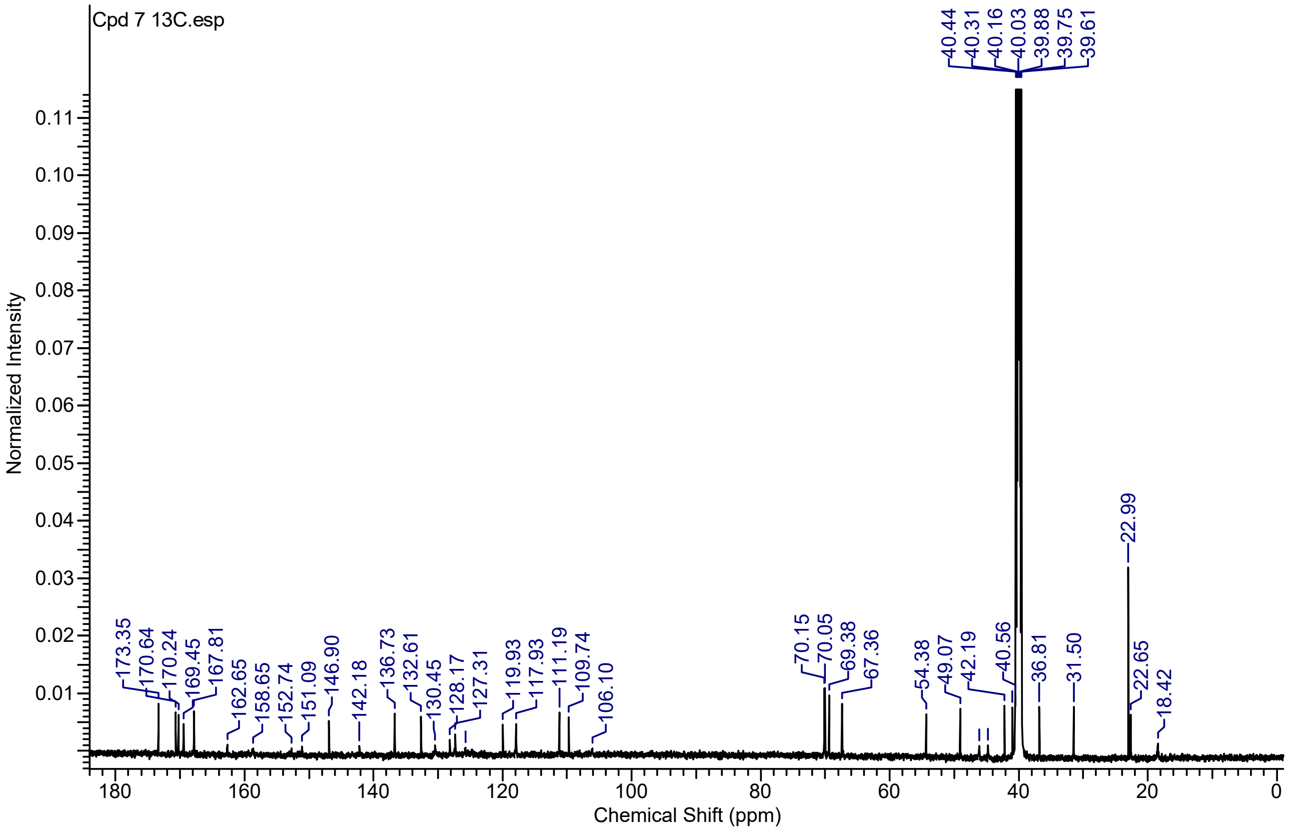
Cpd 7 13C.esp
Normalized Intensity
Chemical Shift (ppm)
173.35
170.64
170.24
169.45
167.81
162.65
158.65
152.74
151.09
146.90
142.18
136.73
132.61
130.45
128.17
127.31
119.93
117.93
111.19
109.74
106.10
70.15
70.05
69.38
67.36
54.38
49.07
42.19
40.56
40.44
40.31
40.16
40.03
39.88
39.75
39.61
36.81
31.50
22.99
22.65
18.42

Compound **8**

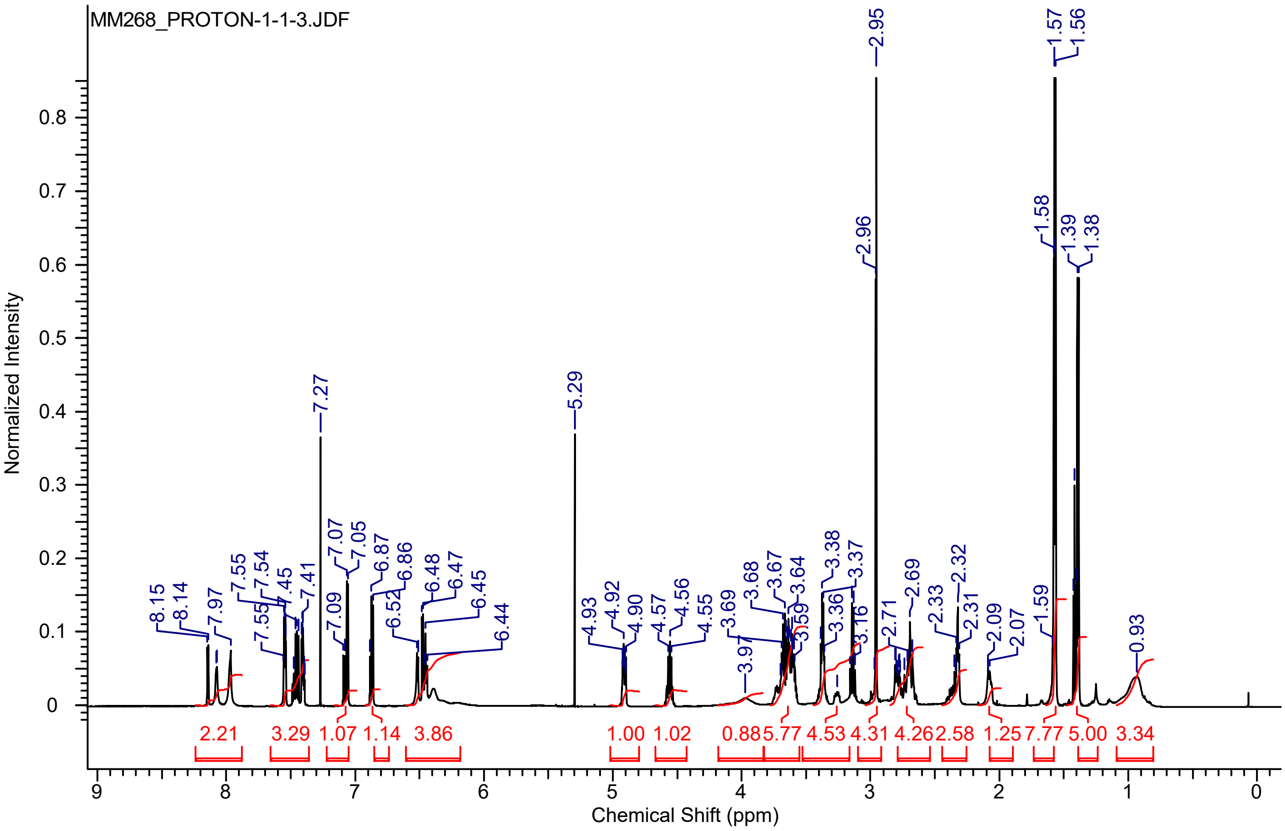
MM268_PROTON-1-1-3.JDF
Normalized Intensity
Chemical Shift (ppm)
8.15 8.14 7.97 7.55 7.54 7.55 7.45 7.41 7.27 7.09 7.07 7.05 6.87 6.86 6.52 6.48 6.47 6.45 6.44 5.29 4.93 4.92 4.90 4.57 4.56 4.55 3.97 3.69 3.68 3.67 3.64 3.59 3.38 3.37 3.36 3.16 2.96 2.95 2.71 2.69 2.33 2.32 2.31 2.09 2.07 1.59 1.58 1.57 1.56 1.39 1.38 0.93
2.21 3.29 1.07 1.14 3.86 1.00 1.02 0.88 5.77 4.53 4.31 4.26 2.58 1.25 7.77 5.00 3.34

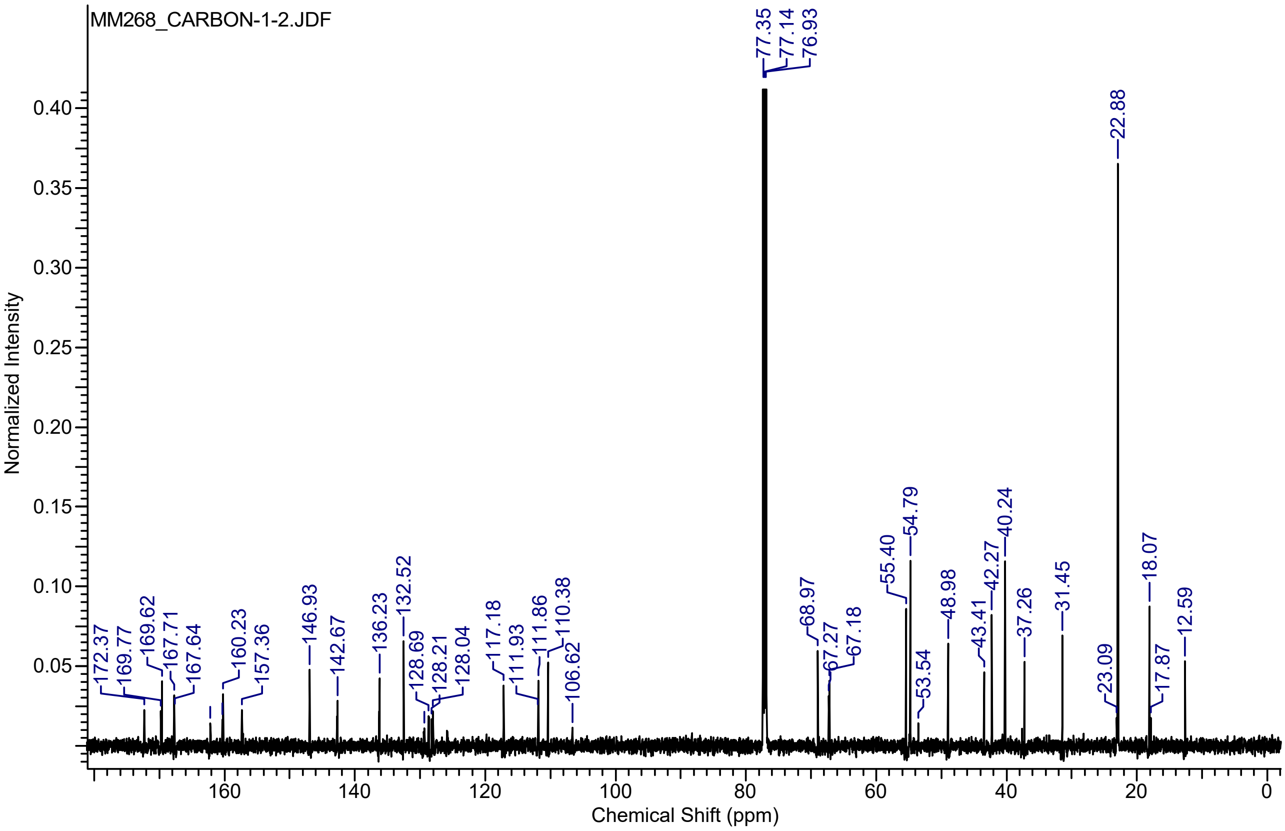
MM268_CARBON-1-2.JDF
Normalized Intensity
Chemical Shift (ppm)
172.37 169.77 169.62 167.71 167.64 160.23 157.36 146.93 142.67 136.23 132.52 128.69 128.21 128.04 117.18 111.93 111.86 110.38 106.62 77.35 77.14 76.93 68.97 67.27 67.18 55.40 54.79 53.54 48.98 43.41 42.27 40.24 37.26 31.45 23.09 22.88 18.07 17.87 12.59

Compound **9**

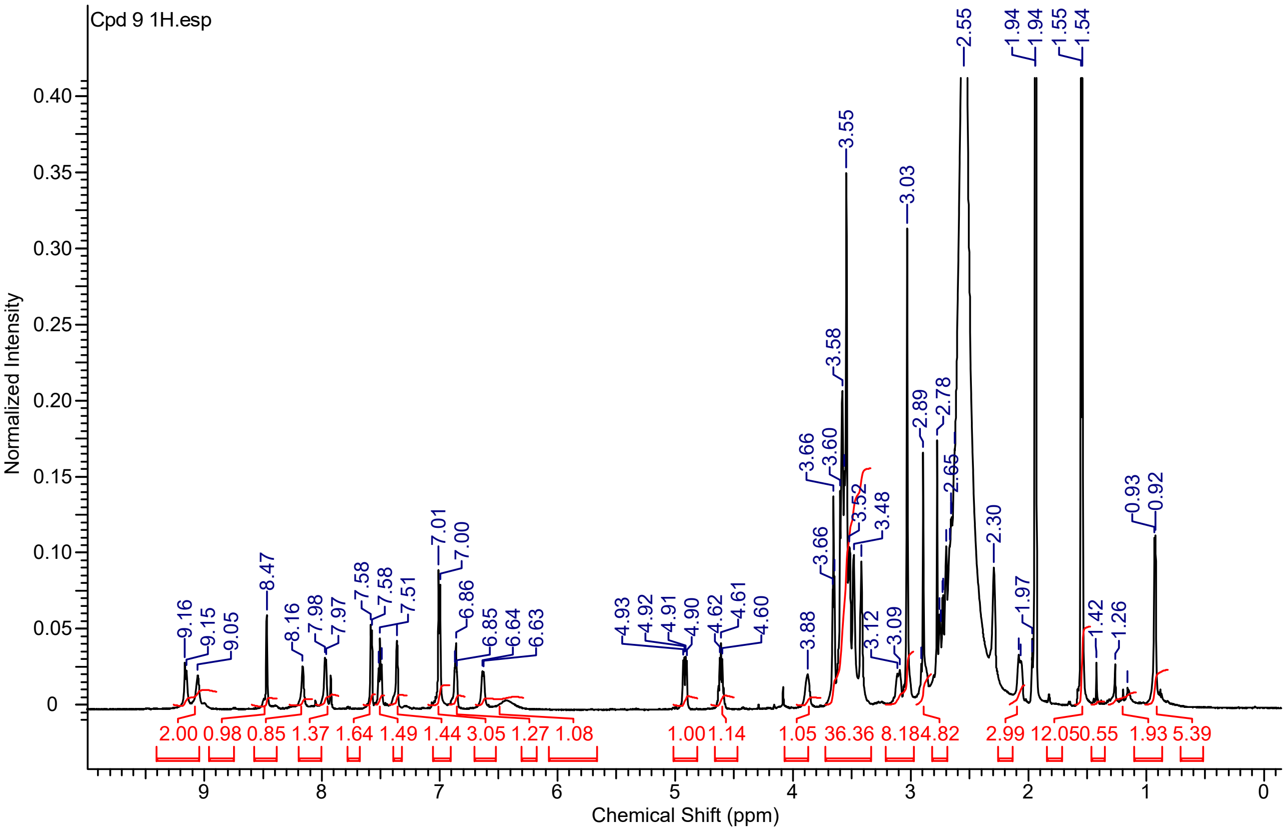
Cpd 9 1H.esp
Normalized Intensity
Chemical Shift (ppm)
0.40
0.35
0.30
0.25
0.20
0.15
0.10
0.05
0
9
8
7
6
5
4
3
2
1
0

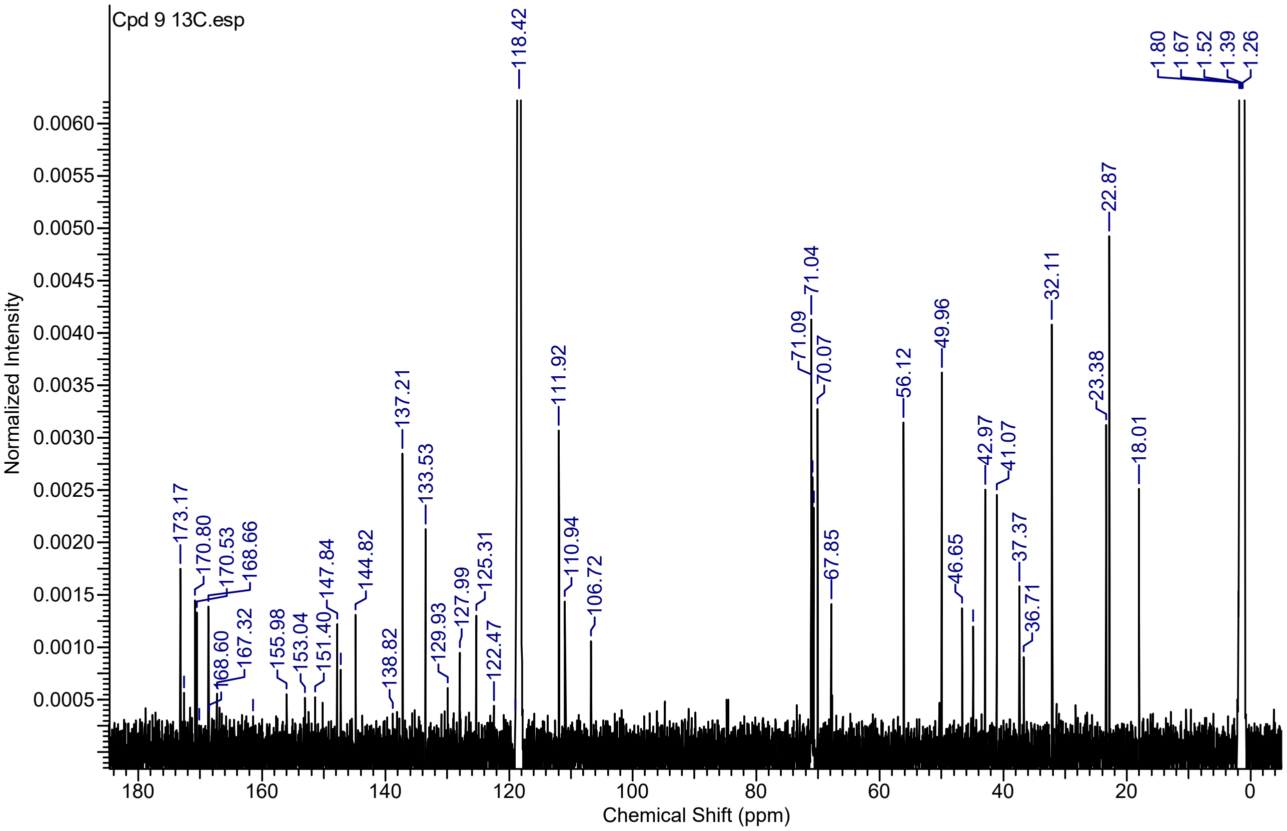
Cpd 9 13C.esp
Normalized Intensity
Chemical Shift (ppm)
0.0060
0.0055
0.0050
0.0045
0.0040
0.0035
0.0030
0.0025
0.0020
0.0015
0.0010
0.0005
180
160
140
120
100
80
60
40
20
0

Compound **10**

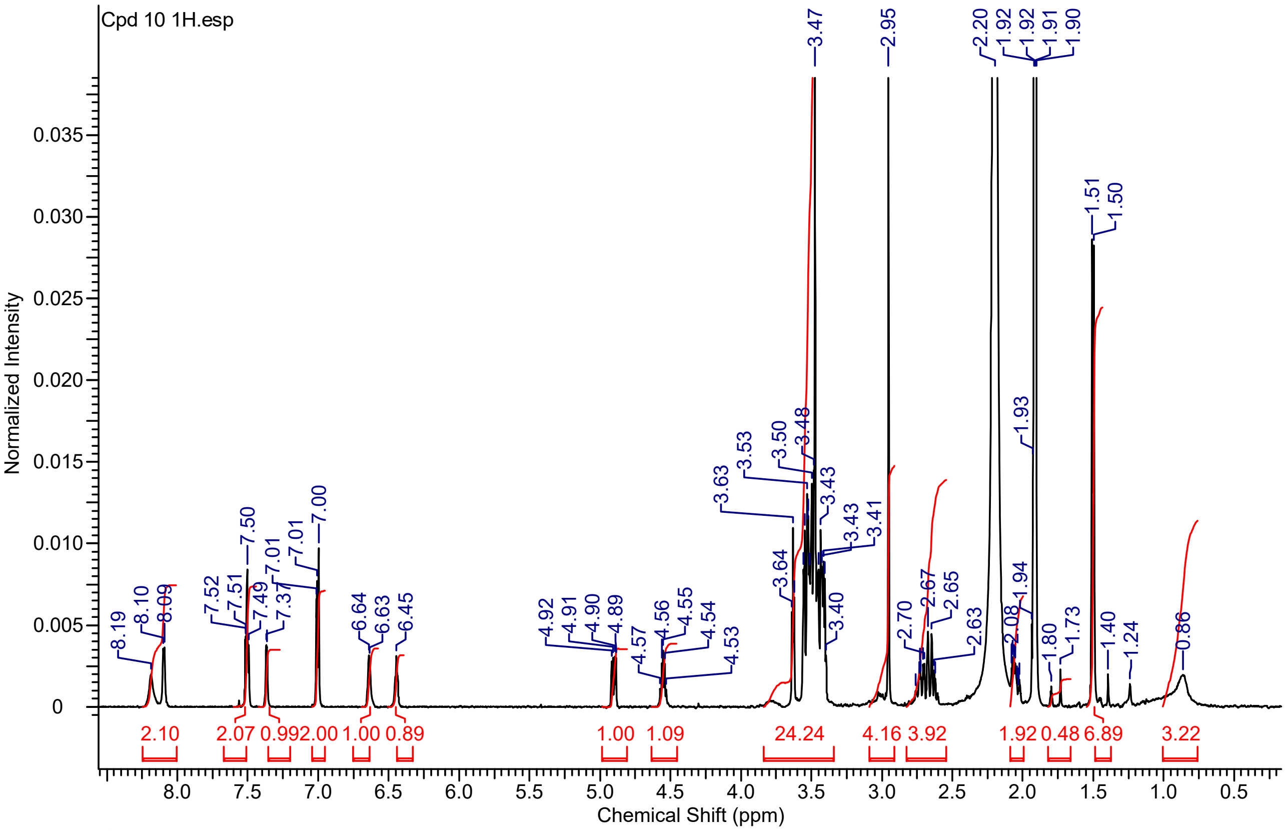
Cpd 10 1H.esp
Normalized Intensity
Chemical Shift (ppm)
8.19 8.10 8.09 7.52 7.51 7.50 7.49 7.37 7.01 7.01 7.00 6.64 6.63 6.45 4.92 4.91 4.90 4.89 4.57 4.56 4.55 4.54 4.53 3.64 3.63 3.53 3.50 3.48 3.47 3.43 3.43 3.41 3.40 2.95 2.70 2.67 2.65 2.63 2.20 2.08 1.94 1.93 1.92 1.92 1.91 1.90 1.80 1.73 1.51 1.50 1.40 1.24 0.86
2.10 2.07 0.99 2.00 1.00 0.89 1.00 1.09 24.24 4.16 3.92 1.92 0.48 6.89 3.22

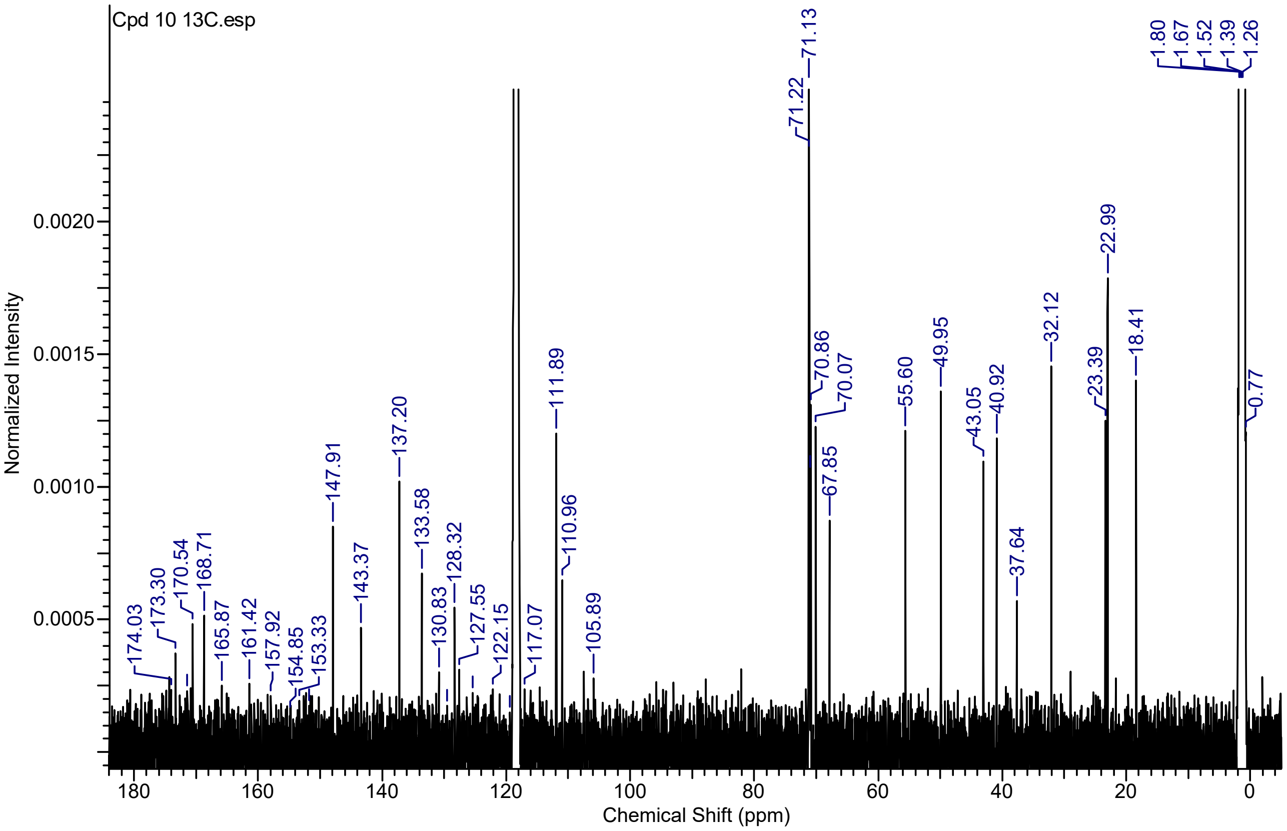
Cpd 10 13C.esp
Normalized Intensity
Chemical Shift (ppm)
174.03 173.30 170.54 168.71 165.87 161.42 157.92 154.85 153.33 147.91 143.37 137.20 133.58 130.83 128.32 127.55 122.15 117.07 111.89 110.96 105.89 71.22 71.13 70.86 70.07 67.85 55.60 49.95 43.05 40.92 37.64 32.12 23.39 22.99 18.41 1.80 1.67 1.52 1.39 1.26 0.77

Compound **11**

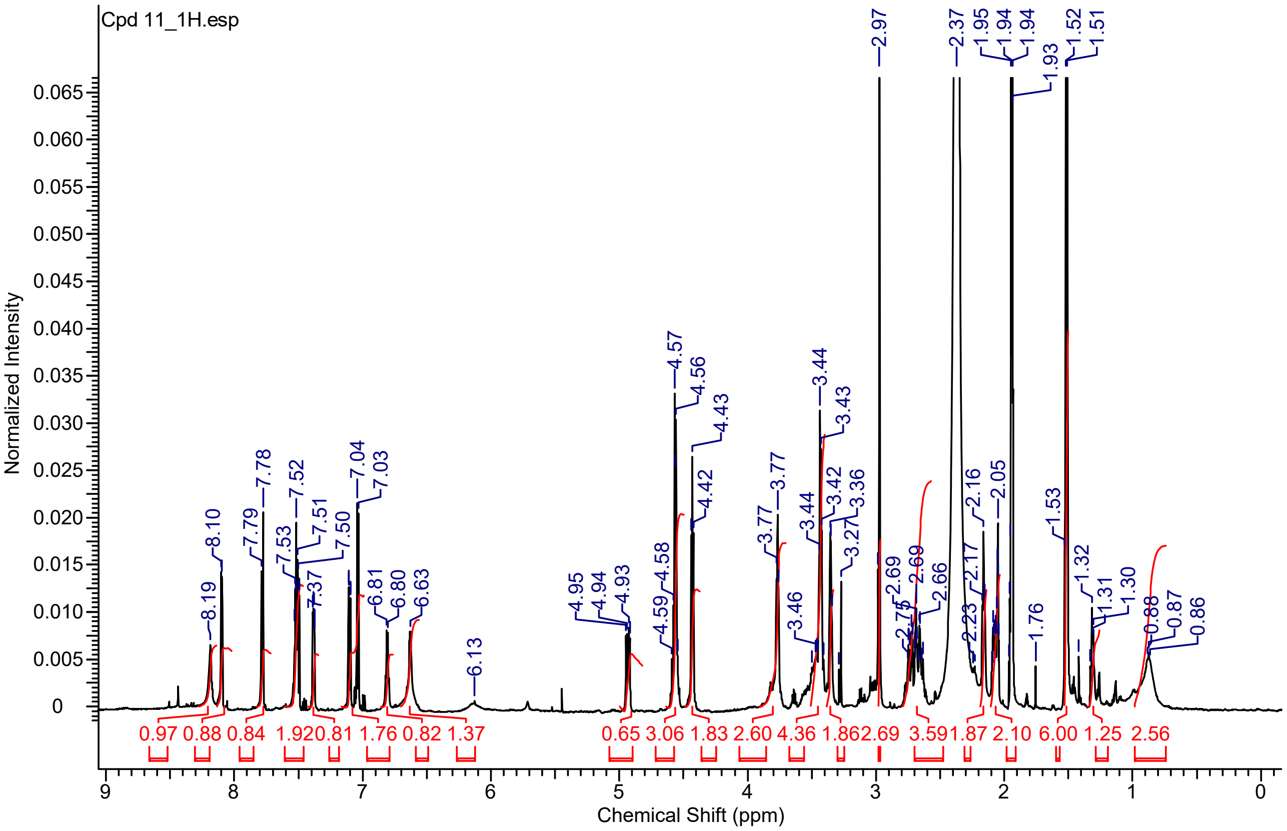

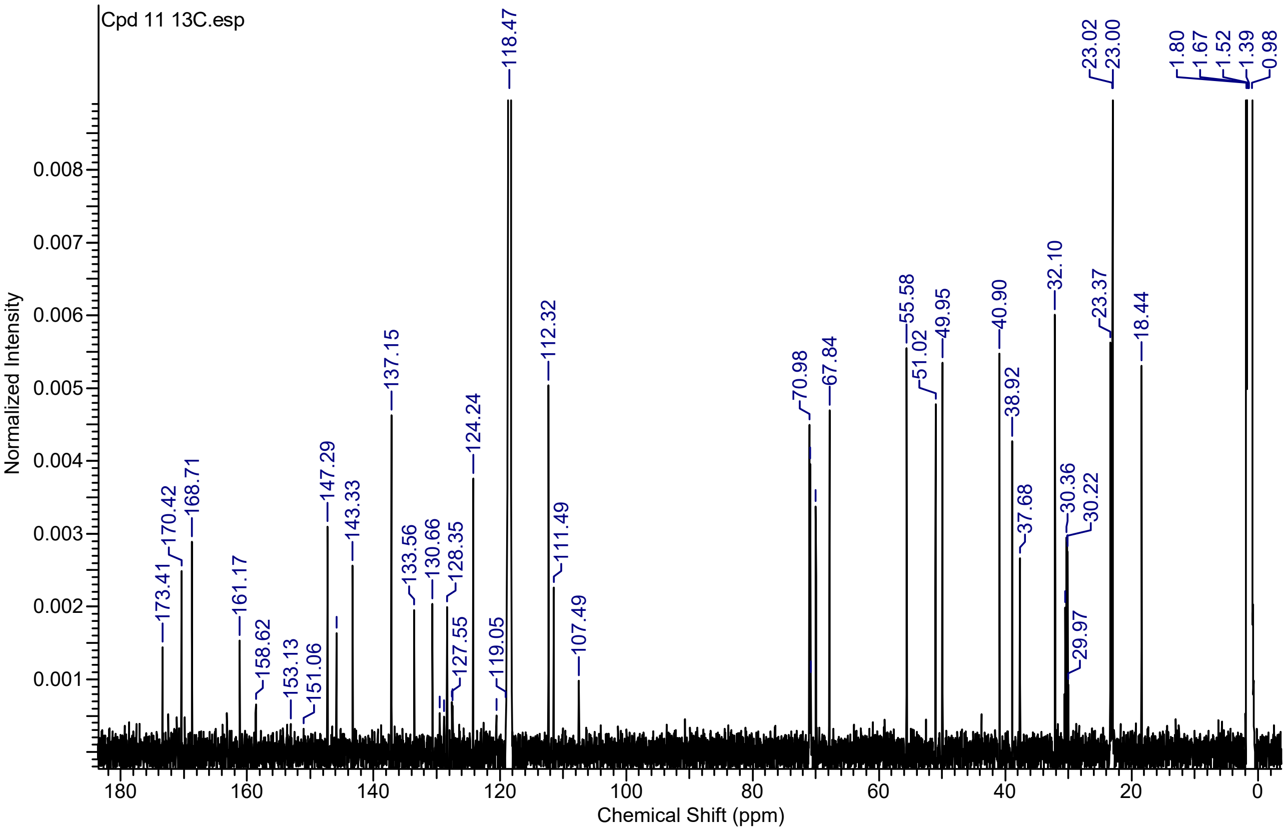

Compound **12**

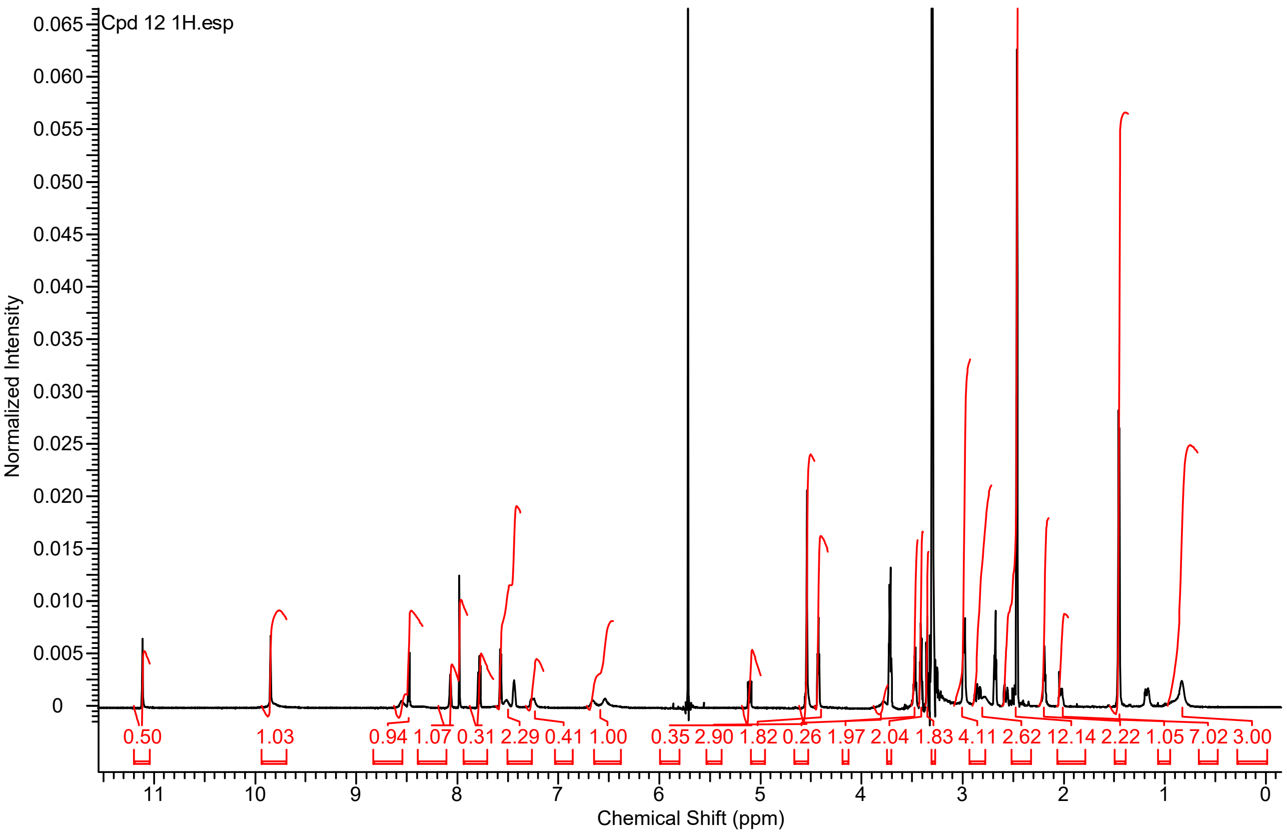
Cpd 12 1H.esp
Normalized Intensity
Chemical Shift (ppm)
0.50 1.03 0.94 1.07 0.31 2.29 0.41 1.00 0.35 2.90 1.82 0.26 1.97 2.04 1.83 4.11 2.62 12.14 2.22 1.05 7.02 3.00

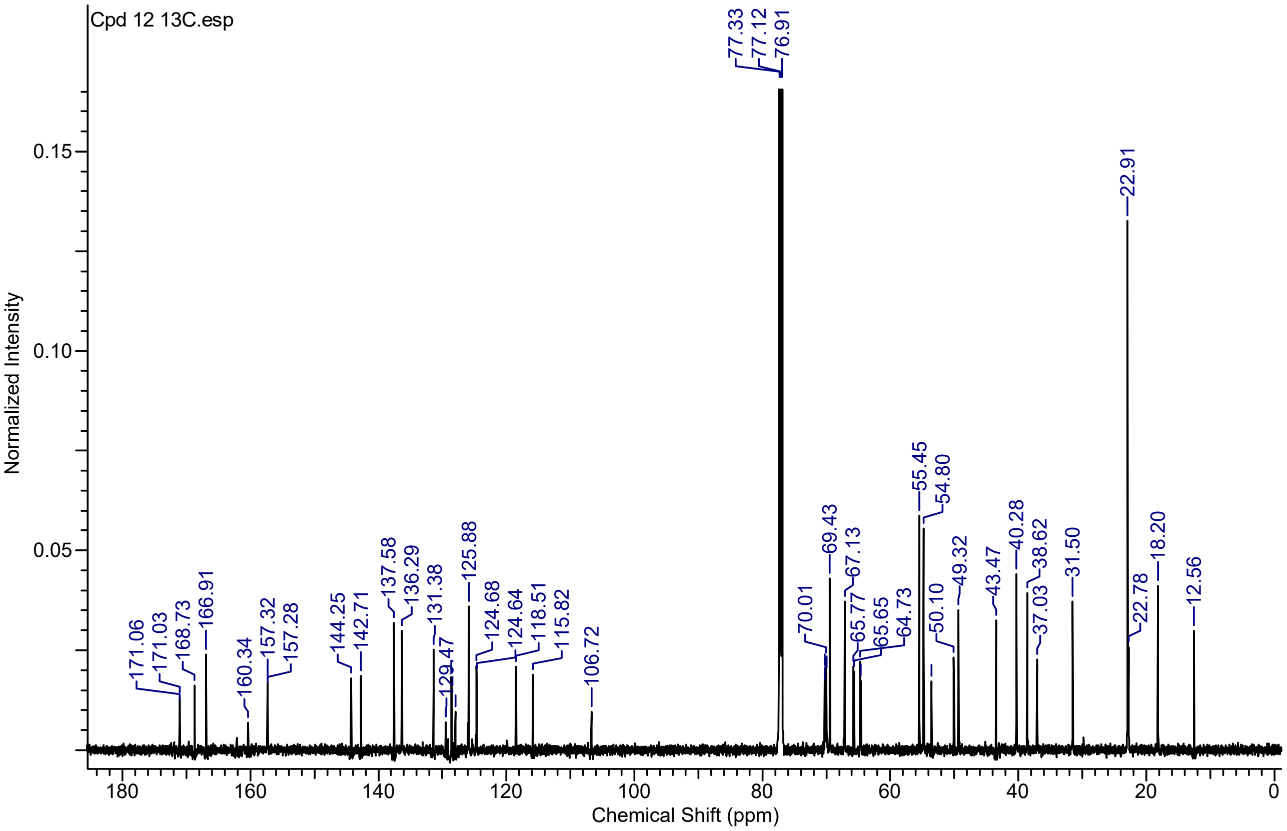
Cpd 12 13C.esp
Normalized Intensity
Chemical Shift (ppm)
77.33 77.12 76.91
22.91
171.06 171.03 168.73 166.91 160.34 157.32 157.28 144.25 142.71 137.58 136.29 131.38 129.47 125.88 124.68 124.64 118.51 115.82 106.72
70.01 69.43 67.13 65.77 65.65 64.73 55.45 54.80 50.10 49.32 43.47 40.28 38.62 37.03 31.50 22.78 18.20 12.56

Compound **13**

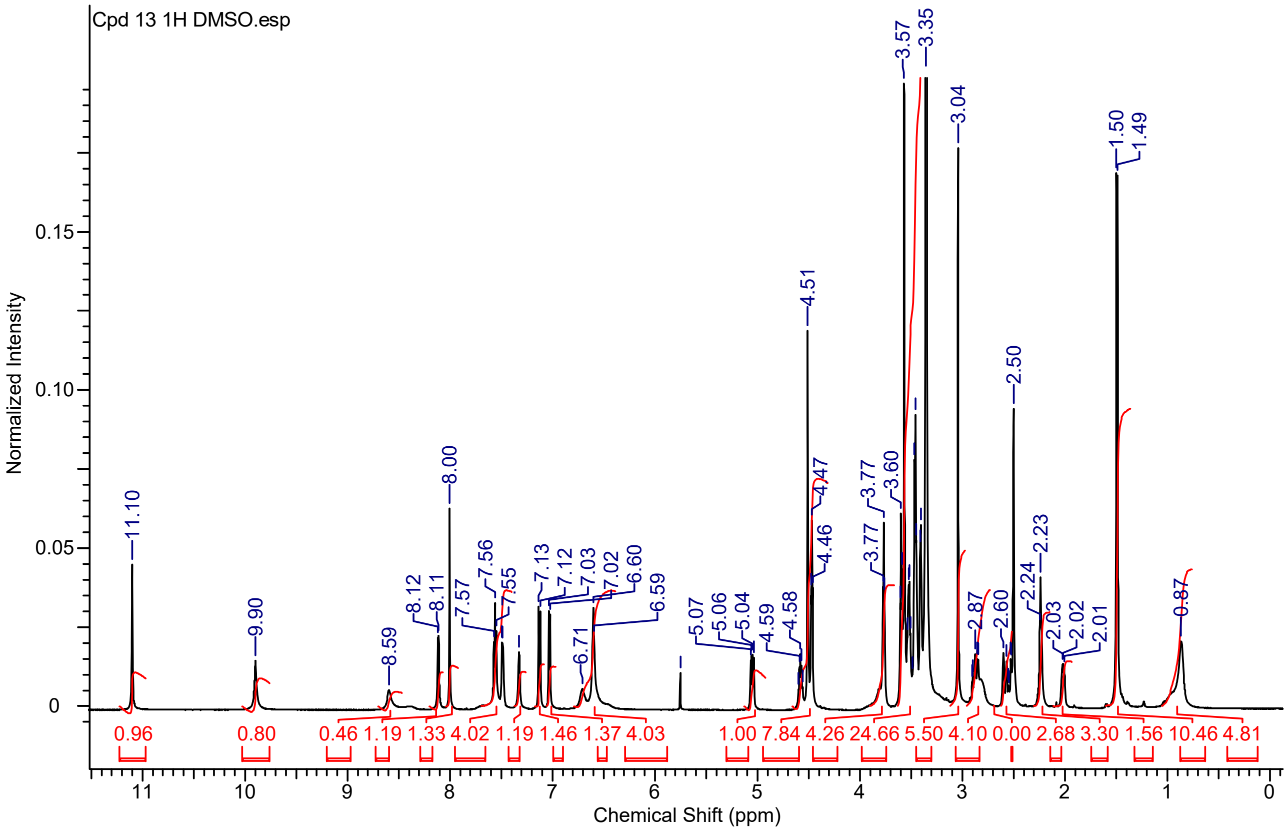
Cpd 13 1H DMSO.esp
Normalized Intensity
Chemical Shift (ppm)

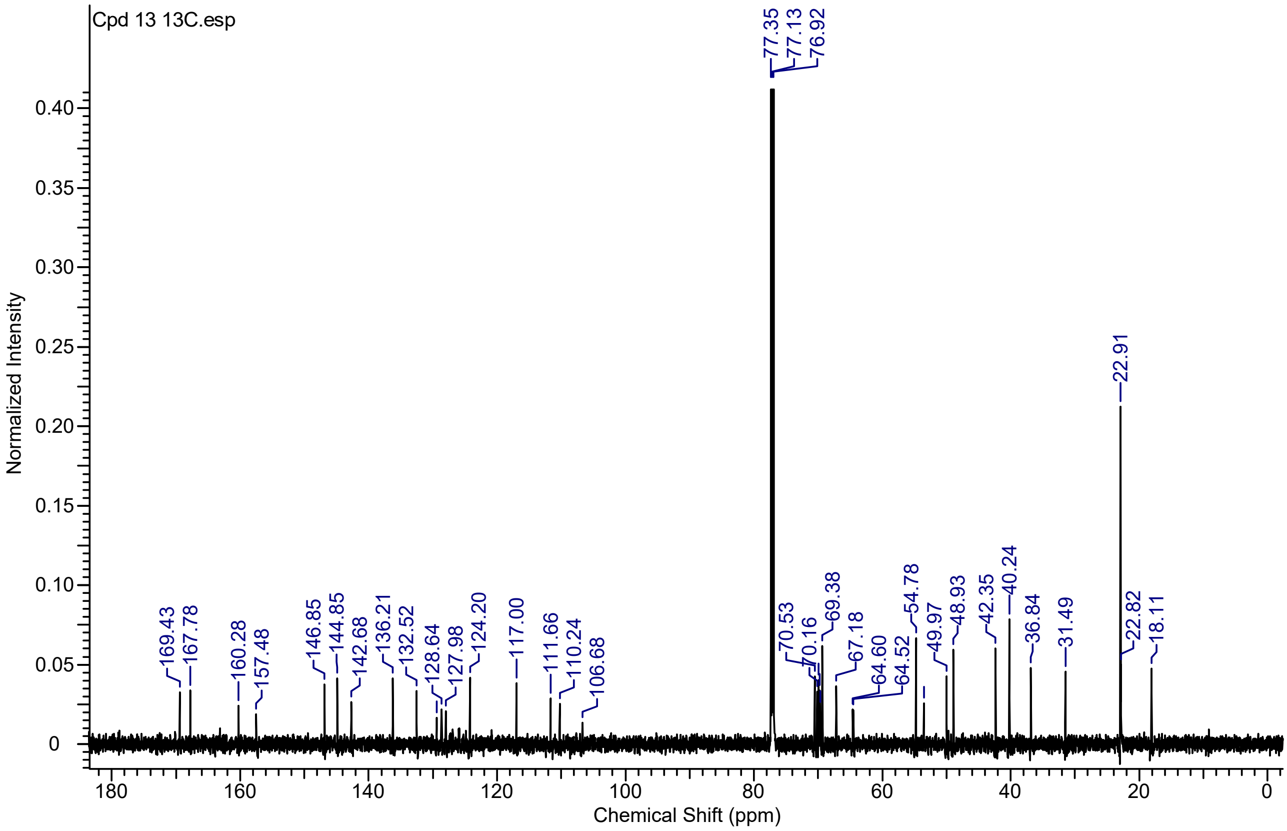
Cpd 13 13C.esp
Normalized Intensity
Chemical Shift (ppm)

Compound **14**

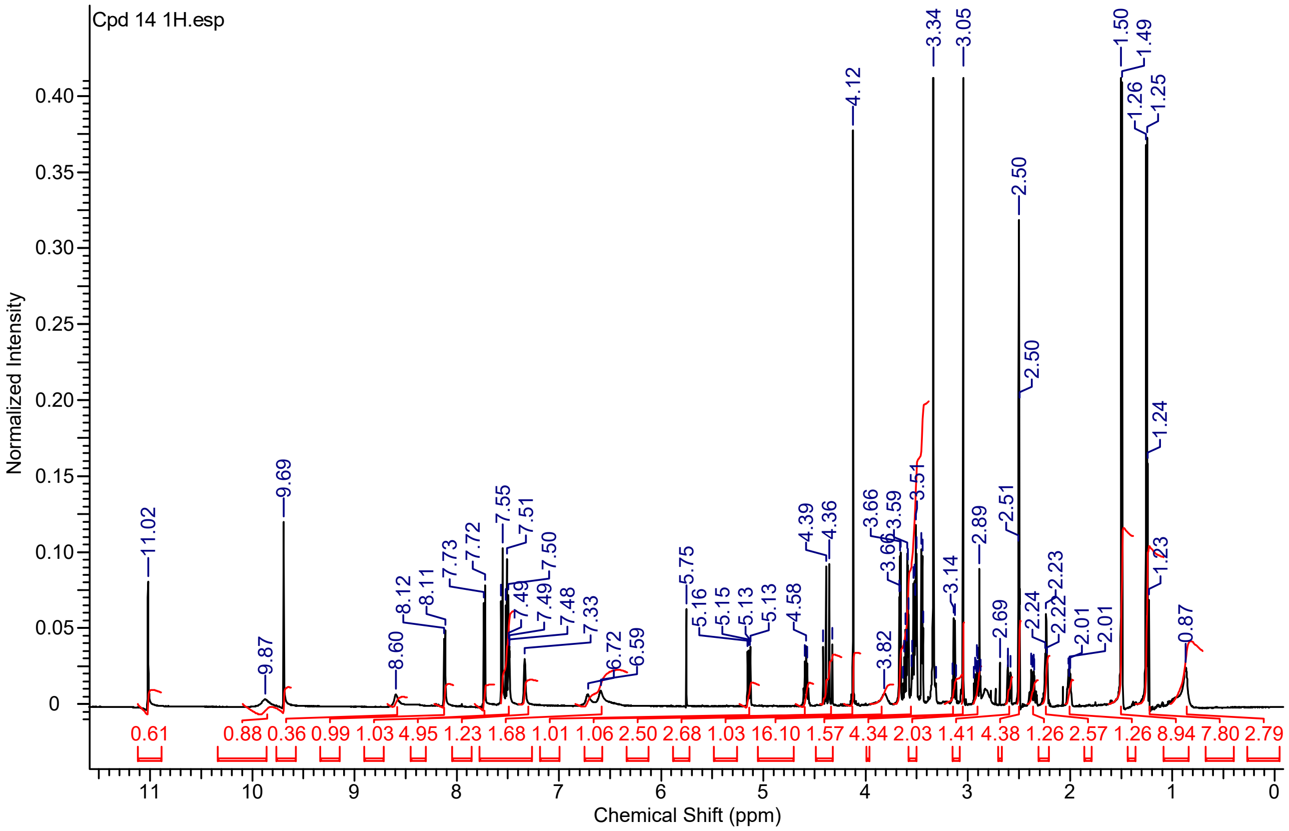
Cpd 14 1H.esp
Normalized Intensity
Chemical Shift (ppm)
11.02
9.87
9.69
8.60
8.12
8.11
7.73
7.72
7.55
7.51
7.50
7.49
7.49
7.48
7.33
6.72
6.59
5.75
5.16
5.15
5.13
5.13
4.58
4.39
4.36
4.12
3.82
3.66
3.60
3.59
3.51
3.34
3.14
3.05
2.89
2.69
2.51
2.50
2.50
2.24
2.23
2.22
2.01
2.01
1.50
1.49
1.26
1.25
1.24
1.23
0.87
0.61 0.88 0.36 0.99 1.03 4.95 1.23 1.68 1.01 1.06 2.50 2.68 1.03 16.10 1.57 4.34 2.03 1.41 4.38 1.26 2.57 1.26 8.94 7.80 2.79

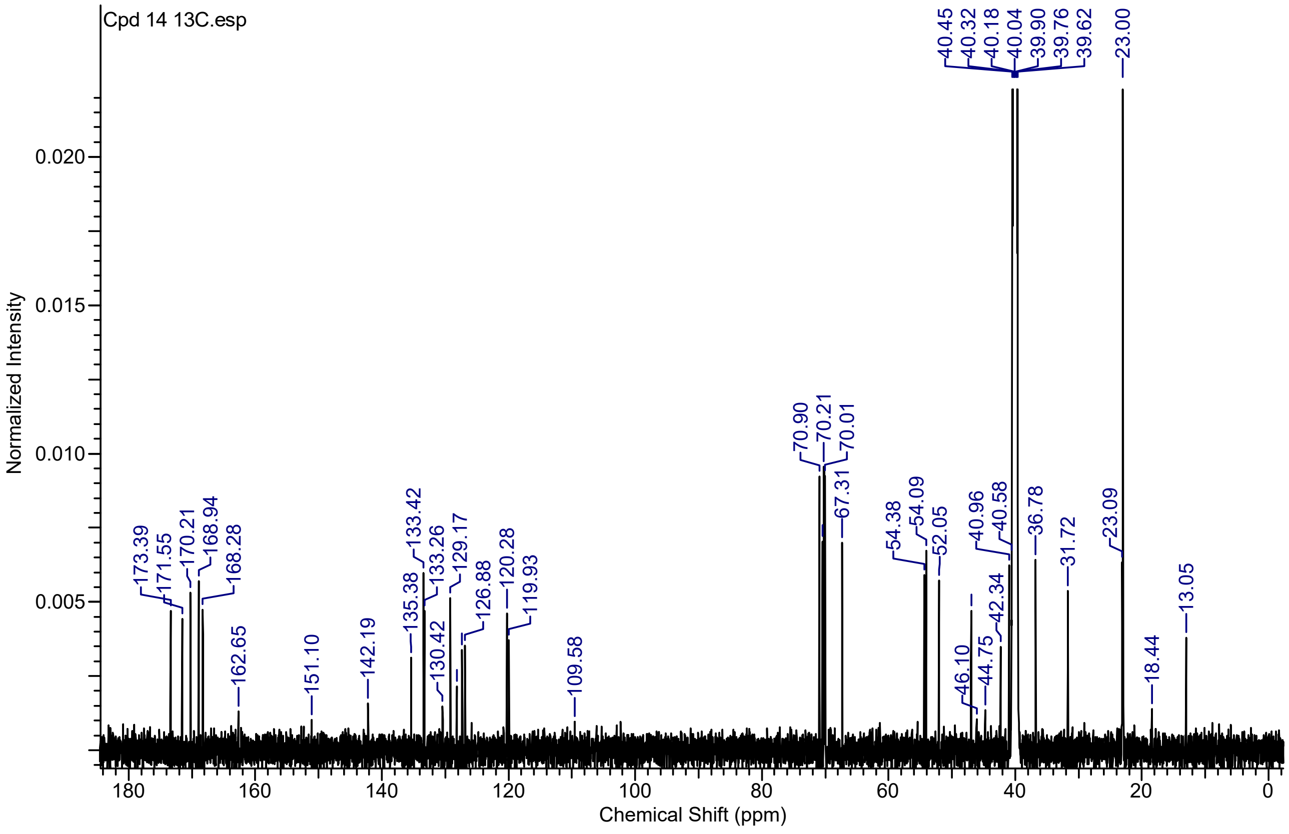
Cpd 14 13C.esp
Normalized Intensity
Chemical Shift (ppm)
173.39
171.55
170.21
168.94
168.28
162.65
151.10
142.19
135.38
133.42
133.26
130.42
129.17
126.88
120.28
119.93
109.58
70.90
70.21
70.01
67.31
54.38
54.09
52.05
46.10
44.75
42.34
40.96
40.58
40.45
40.32
40.18
40.04
39.90
39.76
39.62
36.78
31.72
23.09
23.00
18.44
13.05

Compound **15**

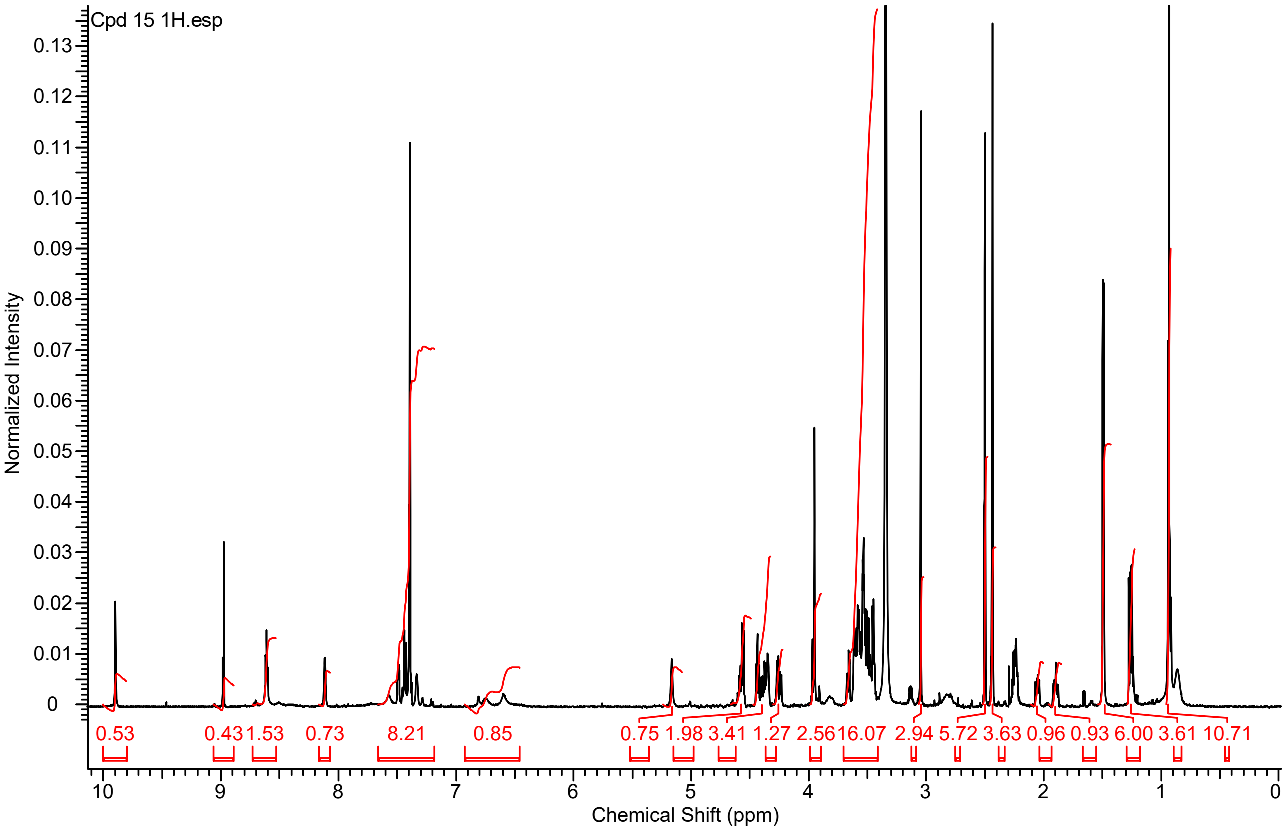
Cpd 15 1H.esp
Normalized Intensity
Chemical Shift (ppm)
0.53 0.43 1.53 0.73 8.21 0.85 0.75 1.98 3.41 1.27 2.56 16.07 2.94 5.72 3.63 0.96 0.93 6.00 3.61 10.71

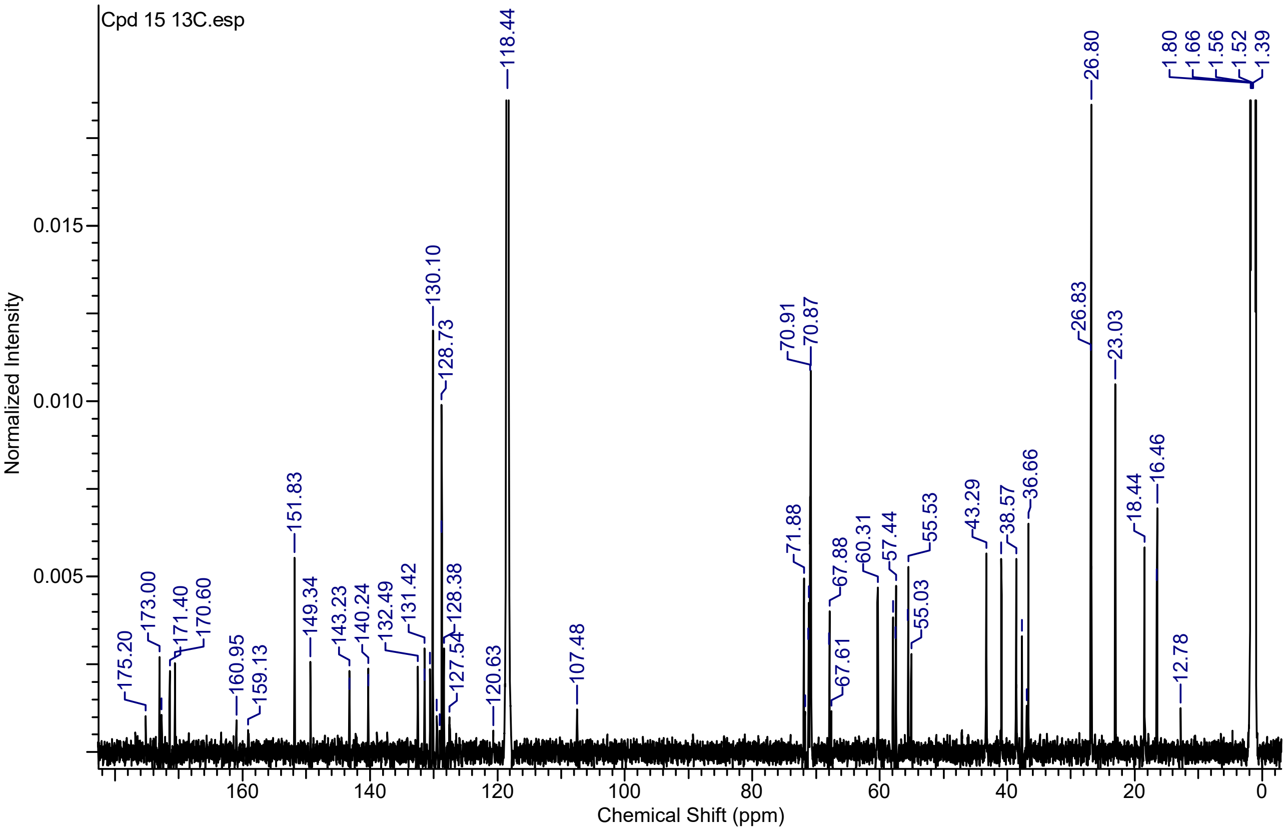
Cpd 15 13C.esp
Normalized Intensity
Chemical Shift (ppm)
175.20 173.00 171.40 170.60 160.95 159.13 151.83 149.34 143.23 140.24 132.49 131.42 130.10 128.73 128.38 127.54 120.63 118.44 107.48
71.88 70.91 70.87 67.88 67.61 60.31 57.44 55.53 55.03 43.29 38.57 36.66 26.83 26.80 23.03 18.44 16.46 12.78 1.80 1.66 1.56 1.52 1.39
